# Theoretical Studies on the Reaction Mechanism for the Cycloaddition of Zwitterionic π-Allenyl Palladium Species: Substrate-Controlled Isomerization

**DOI:** 10.3390/molecules30010103

**Published:** 2024-12-30

**Authors:** Yongjie Long, Jiahao Shen, Min Shi, Yin Wei

**Affiliations:** State Key Laboratory of Organometallic Chemistry, Center for Excellence in Molecular Synthesis, Shanghai Institute of Organic Chemistry, University of Chinese Academy of Sciences, Chinese Academy of Sciences, 345 Lingling Road, Shanghai 200032, China

**Keywords:** mechanistic studies, palladium catalyzed, zwitterionic π-allenyl palladium species, cycloaddition, substrate-controlled reaction, DFT calculation

## Abstract

Zwitterionic π-allenyl palladium species are newly developed intermediates. A substrate-controlled step existed in the cycloaddition of zwitterionic π-allenyl palladium species with tropsulfimides or tropones. With the assistance of previously experimental studies, zwitterionic allenyl/propargyl palladium species was provenly found by HRMS. Further DFT calculation studies show that zwitterionic π-allenyl palladium species are generated through the oxidative addition of Pd(0), which can be promoted by Lewis acid like Yb(OTf)_3_, and the cycloaddition more likely undergoes through an outer sphere nucleophilic attack. The isomerization is caused by the difference of dissociation energy between the cycloaddition intermediation of tropsulfimides and tropones, forming the substrate-controlled specificity.

## 1. Introduction

**Scheme 1:**
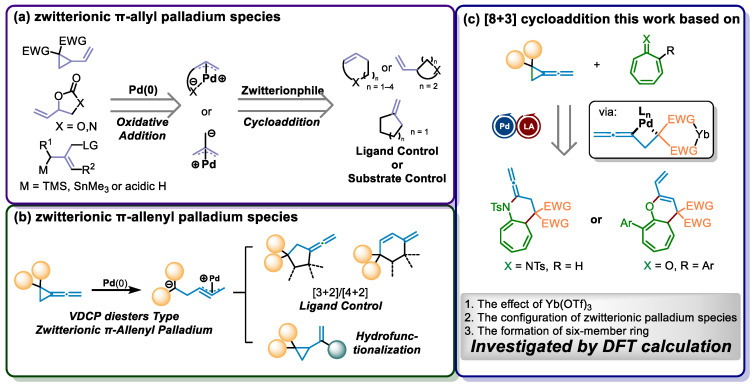
Previous works of zwitterionic palladium species chemistry and former substrate-control [8 + 3] cycloaddition reaction DFT research based on.

As an important type of intermediate, zwitterionic π-allyl palladium species play a constructive role in modern organic chemistry methodology [1,2,3,4,5,6]. During the long period of study in such a field, mature mechanisms of reactions in the field of zwitterionic π-allyl palladium species have already been deeply explored (Scheme 1a), and density functional theory (DFT) calculations were proved to be a powerful and effective tool for exploring those mechanistic insights [7,8,9,10,11,12,13,14]. Nevertheless, the research on zwitterionic π-allenyl palladium species, resembling zwitterionic π-allyl palladium species, is still in the ascendant.

In 2021, the first example of a cycloaddition reaction via the zwitterionic π-allenyl palladium species generated from vinylidenecyclopropane-diesters (VDCP-diesters) with electron-withdrawing groups (EWG) was reported by our group [15], which exhibited a ligand-controlled selectivity in these reactions: using DPEPhos as ligand (L_n_) preferred to induce [3 + 2] cycloaddition, while employing dppp as a ligand tended to induce the cycloaddition with a [4 + 2] mode. However, the underlying logic of the mechanism of such a cycloaddition reaction was still ambiguous. On the other hand, another type of reaction mode of zwitterionic π-allenyl palladium species, a hydroamination reaction, was found in 2022 by our group (Scheme 1b) [16]. Although preliminarily experimental studies on the reaction mechanism were shown in that work, it still lacked a coherent explanation about the mechanism. To their credit, some follow-up studies offset the lack of mechanism in the former type of reaction [17] and the latter type of reaction [18], whereas the systematic mechanism studies remain deficient.

Recently, our group reported a substrate-controlled [8 + 3] cycloaddition of tropone derivative via zwitterionic π-allenyl palladium species (Scheme 1c) [19]. As an extension of traditional type cycloaddition, the difference between tropsulimides and tropones led to different products: the former substrate afforded allene products at 70 °C in 12 h, at a relatively higher temperature with a shorter reaction time, and the latter afforded conjugated dienes at 50 °C in 24 h, which was at a much milder temperature with a longer time. Hence, a plausible mechanism based on an isomerization, which might occur after the formation of the allene part, was raised. However, no direct evidence of the allene intermediate of tropone has been discovered, while a series of problems, such as the effect of Lewis acid additive and the type of C–N or C–O bond formation, are still unclear. Detailed mechanistic studies on the reaction mechanism are still highly demanded. In this work, we attempt to provide a thorough understanding of the reaction mechanism as well as the nature of the chemoselectivity via theoretical investigation by DFT calculations. In this work, the effect of Lewis acid additives like Yb(OTf)_3_ is first expounded, which can highly promote the oxidative addition of C–C bonds, and the different types of cycloaddition, outer sphere nucleophilic attack or inner sphere reductive elimination, are equally compared. Combined with the calculation of isomerization, the mechanism of this substrate-controlled reaction can be unveiled in development.

## 2. Results and Discussion

### 2.1. Mechanistic Studies by Experiments

**Scheme 2:**
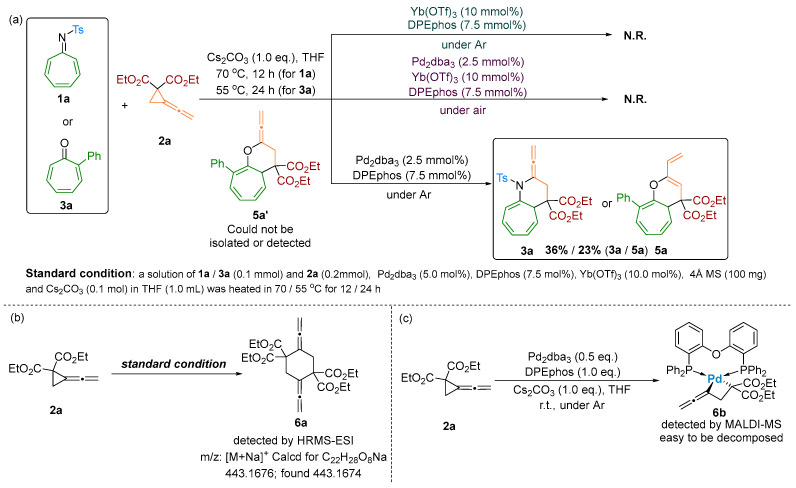
(**a**) Control experiments. (**b**) Detections of key zwitterionic palladium species through the dimer of intermediate. (**c**) Detections of key zwitterionic palladium species by MALDI-MS.

In a previous report, we performed a few control experiments to investigate the mechanism of this palladium-catalyzed reaction preliminarily [19]. Such experiments, which specifically filtrated 2.5 mol% palladium resource Pd_2_(dba)_3_ or 10 mol% Yb(OTf)_3_ in the reaction of 0.1 mmol **1a** or **4a** with 0.2 mmol **2a** with 7.5 mol% DPEphos, 100 mg 4Å MS, and 0.1 mmol Cs_2_CO_3_ in 1.0 mL THF heated in 70 °C (for **1a**) or 55 °C (for **4a**) for 12 (for **1a**) or 24 (for **4a**) h, were repeated and presented the same results as shown in the previous work (see part D in Supporting Information). The reaction afforded product **3a** in 36% yield under standard reaction conditions only without the Lewis acid (LA) additive Yb(OTf)_3_; however, the reaction did not occur when the palladium catalyst was removed. We replenished the control experiments of **3a**, which presented a similar pattern. The possibility of Lewis acid as the main catalyst was considered unreasonable, and the Lewis acid Yb(OTf)_3_ basically acted as an additive to promote this palladium-catalyzed reaction (Scheme 2a).

Based on such experiments, further experiments were performed with the help of mass spectra. After the reaction under standard conditions, only using VDCP diester **2a** as the reactant, a VDCP dimer **6a** was detected through high-resolution mass spectrum-electrospray ionization (HRMS-ESI), pointing to the existence of zwitterionic π-allenyl palladium species (Scheme 2b). Moreover, an allenyl palladium complex **6b**, prepared from Pd_2_dba_3_, DPEphos, and VDCP-diester **2a**, was observed by MALDI-MS, providing the first experimental evidence on the formation of a zwitterionic allenyl palladium species and suggesting that the zwitterionic palladium species might be derived from the oxidative addition of a palladium catalyst with the cyclopropane ring in VDCP-diester (Scheme 2c).

However, the exact structure of the zwitterionic palladium species, which had an allenyl or propargyl or any other group and malonic ester anion coordinated to the palladium center or not, was difficult to identify by the current experimental techniques because those zwitterionic palladium species made from VDCP diesters were all unstable in a series of solvents, which could only keep a weak chemical equilibrium between zwitterionic palladium species and VDCP diesters even with the help of base to restrain the oligomerization of VDCP diesters. Otherwise, a semi-solid mixture with solvent would be generated, which was totally unanalyzable in terms of molecular structure, let alone the formation of monocrystal. This means that the conformation of the exact structure of zwitterionic palladium species through experiments like X-ray diffraction (XRD) is almost impossible to confirm. On the other hand, the allenyl product of tropone **5a′** could not be isolated or detected, leaving a mystery in the rearrangement part. Thus, DFT studies are indispensable to be employed for investigations on the structures of zwitterionic palladium species and the mechanism for the rearrangement part of this reaction.

### 2.2. Proposed Cycloaddition and Rearrangement Reaction Pathways

**Scheme 3:**
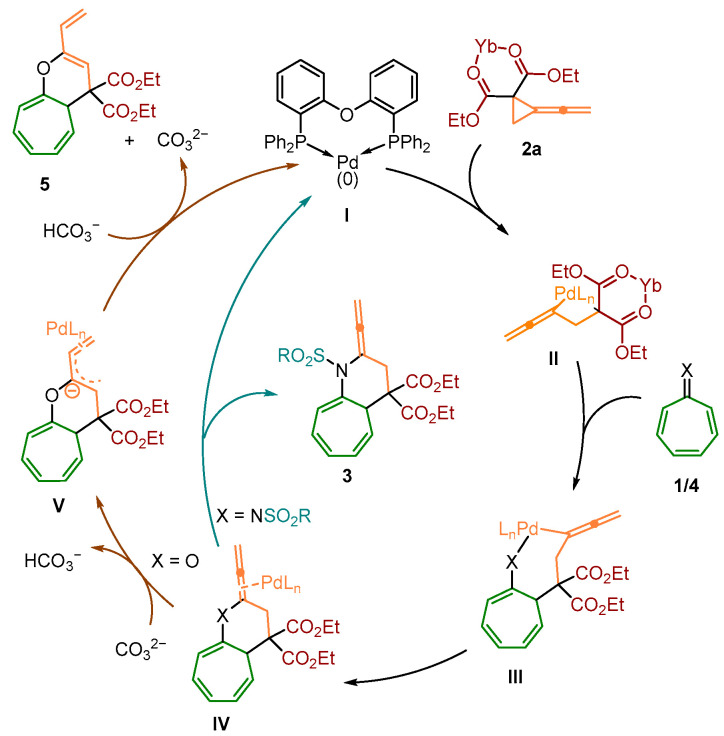
Proposed reaction pathways.

According to former experimental studies, we proposed a plausible reaction pathway (Scheme 3). On the [8 + 3] cycloaddition part, an oxidative addition between palladium(0) complex **I** and VDCP-diester **2a**, [1,12,15,16,17,18,19,20,21], activated by Lewis acid additive Yb(OTf)_3_, first took place at the electron-deficient C–C site of the cyclopropyl unit to form an intermediate **II**, which underwent the cycloaddition reaction with tropsulfimide **1** or tropone derivative **4** to afford intermediate **III**. Although we drew an allenyl moiety in the intermediates **II** and **III**, π-propargyl still might exist in them as well as in intermediate **IV**, which would be mentioned afterwards. Significantly, whether the X part coordinated to the palladium center or not depends on the type of palladium species, which means the X part should coordinate to palladium if the palladium center coordinated to allenyl but should not with palladium while the palladium center combined with π-propargyl [22,23].

Since then, an intermediate **IV** was generated via a chair-form reductive elimination or an X-leading nucleophilic attack. On the rearrangement part, when X = NSO_2_R, a simple dissociation generated the desired product **3** and regenerated the Pd(0) catalyst. When X = O, we supposed that an uncommon palladium-mediated proton transfer driven by carbonate anion would occur, producing the allyl anionic intermediate **V**. Through a proton-transfer shuttle, the protonation of **V** on another side of the allyl anion with the bicarbonate anion afforded product **5** and regenerated the Pd catalyst.

### 2.3. Theoretical Investigation on a Possible Reaction Pathway

**Scheme 4:**
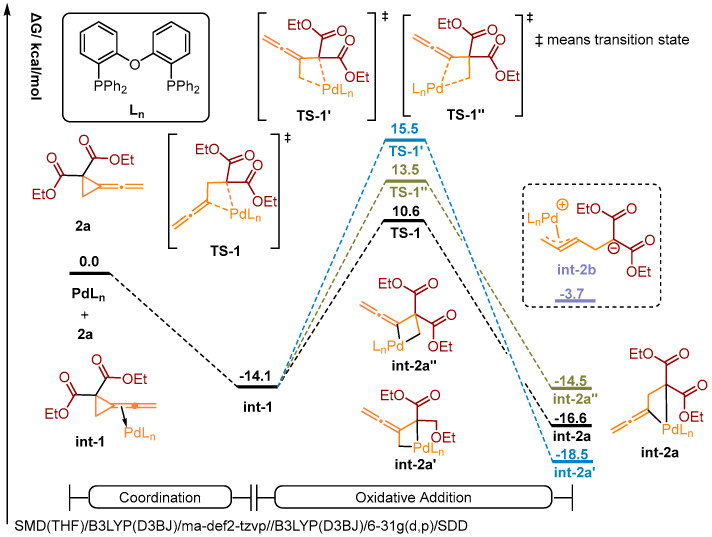
DFT calculation studies of the oxidative addition process.

First of all, which C–C bond on the cyclopropane ring of the VDCP diester the oxidative addition took place should be worked out. We investigated the reaction pathway starting from a stable palladium complex **int-1** as shown in Scheme 4, in which the allene unit of **2a** is coordinated to the DPEphos-Pd(0) catalyst. The palladium complex **int-1** undergoes an oxidative addition through **TS-1** to form a zwitterionic allenyl palladium intermediate **int-2a** with an energy barrier of 24.7 kcal/mol in an exothermic process (ΔG = −2.5 kcal/mol). Another possible zwitterionic π-propargyl palladium intermediate **int-2b** is also investigated. However, compared with the η^1^-coordinated intermediate **int-2a**, the energy of the η^3^-coordinated intermediate **int-2b** is much higher by 13.4 kcal/mol, probably due to the steric effect derived from DPEphos and the decentralization of the anion-cation part. We have also investigated two other ring-open pathways, which are exhibited as passing transition state **TS-1′** to give **int-2a′** and passing transition state **TS-1″** to give **int-2a″**. Both pathways show larger energy gaps than **TS-1** with **int-1**, which are 27.6 kcal/mol and 29.6 kcal/mol. Based on these calculation results, the oxidative addition at the electron-deficient C–C site of the cyclopropyl unit is more reasonable than other types of oxidative addition pathways.

**Scheme 5:**
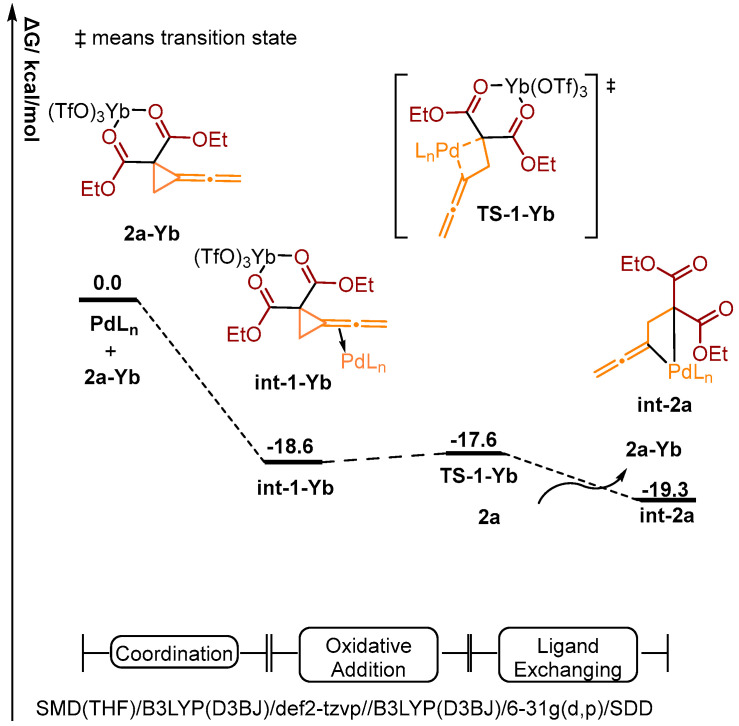
DFT calculation studies of the promotion effect of Yb(OTf)_3_.

As an additive that could raise the reaction efficiency to a large extent, the influence of Lewis acid additive Yb(OTf)_3_ should also be considered and studied (Scheme 5). Compared to the oxidative addition pathway without Yb(OTf)_3_, the additive presents a significant effect to lower the energy gap of ring opening from 24.7 kcal/mol between **int-1** and **TS-1** to 1.0 kcal/mol between **int-1-Yb** and **TS-1-Yb**. The calculation results show that Yb^3+^ coordinated to the carbonyl parts of malonic ester and demonstrate the activation of the Lewis acid additive Yb(OTf) on C–C cleavage of the electron-deficient part of the cyclopropyl unit.

**Scheme 6:**
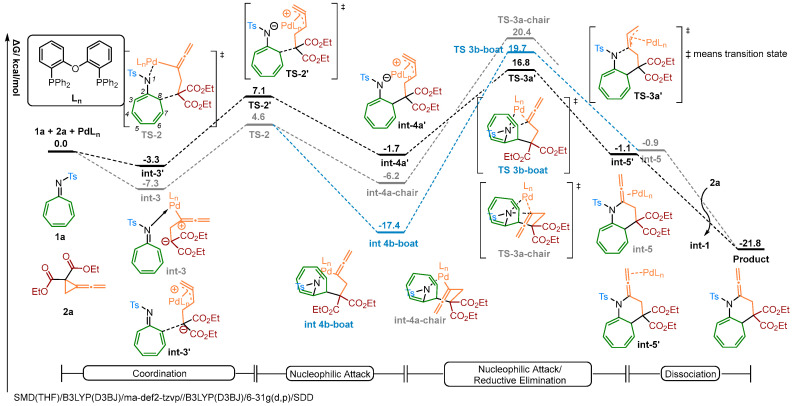
DTF calculation studies of the [8 + 3] cycloaddition mode of tropsulfimide derivative.

Beyond the oxidative addition step, the following ring-closing process was also investigated. As we mentioned above, the allenyl might maintain η^1^ type, which is the allenyl-coordinated type, or η^3^ type, which is the π-propargyl-coordinated type, in zwitterionic palladium species. Obviously, there is one more empty hapto in the former type species coordinated with malonic ester anion after oxidative addition, which could be shifted to a Lewis base point such as an N-atom in tropsulfimide or an O-atom in tropone. However, the latter type species could hardly directly coordinate to the palladium center due to the lack of empty hapto. Hence, the allenyl-type species tend to form the ring through a coordinated reductive elimination, while π-propargyl-type species prefer a Lewis-base-part-leading nucleophilic attack. We have tested those hypotheses through the cycloaddition of tropsulfimide **1a** (Scheme 6). Coordination of tropsulfimide **1a** with zwitterionic allenyl palladium intermediate **int-2a** generates an intermediate **int-3,** which overwhelms the transition state **TS-2** with an 11.9 kcal/mol barrier by nucleophilic attack to afford an intermediate **int-4a-chair**. Based on the conformation search, the boat conformer **int-4a-boat** is also located. Although the boat conformer **int-4a-boat** is thermodynamically favorable, it has to overcome a higher energy barrier (ΔG^‡^ = 37.1 kcal/mol) to generate product complex **int-5**; thus, we exclude this pathway. Subsequently, the intermediate **int-4a-chair** overcomes an energy barrier of 26.6 kcal/mol via **TS-3a-chair** to obtain product complex **int-5**. Finally, **int-1** is regenerated through ligand exchanging by **2a** and affords product **3a**, which is nearly irreversible to have the opportunity to rearrange the product. In addition, the π-propargyl-type reaction pathway has also been investigated, which, starting from the coordination of tropsulfimide **1a** with zwitterionic π-propargyl palladium intermediate **int-2b,** generates an intermediate **int-3′** that overwhelms the transition state **TS-2′** with a 10.4 kcal/mol barrier by another type of nucleophilic attack that palladium does not coordinate with NTs to afford **int-4a′**. Overcoming an energy barrier of 18.5 kcal/mol via **TS-3a′**, **int-4a′** can be transformed to **int-5′** through an NTs-leading nucleophilic attack. Finally, product **3a** is afforded by ligand exchanging. In terms of energy barrier, the latter pathway is more kinetically favorable.

**Scheme 7:**
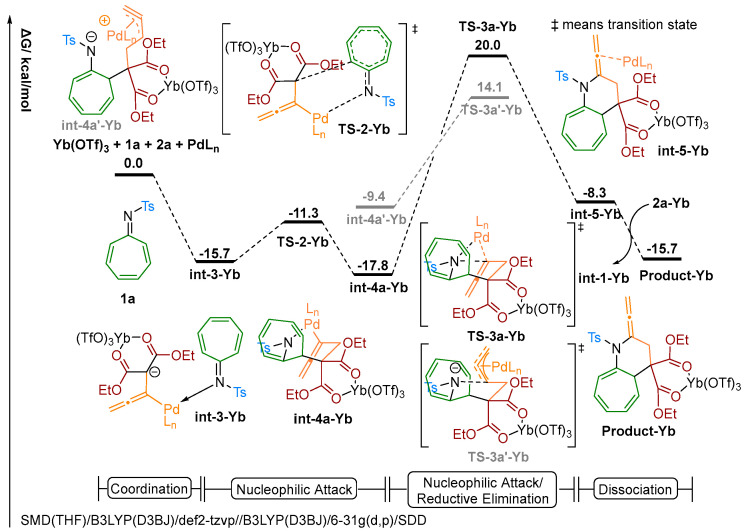
DFT calculation studies on the effect of Yb(OTf)_3_ on the cycloaddition process.

Subsequently, the influence of Yb(OTf)_3_ on the cycloaddition process has also been studied theoretically. The calculation results show that the addition of Yb(OTf)_3_ only has a slight effect on the cycloaddition process and does not significantly influence the reaction barriers and the stabilities of species along the reaction pathway (Scheme 7). Similar to the reaction pathway without Yb(OTf)_3_ (shown in Scheme 6), coordination of tropsulfimide **1a** with zwitterionic allenyl palladium intermediate **int-2a** with Yb^3+^ generates an intermediate **int-3-Yb**. Passing transition state **TS-2a-Yb** with an energy barrier of 4.4 kcal/mol, an intermediate **int-4a-Yb** is obtained in an exothermic process (ΔG = −2.1 kcal/mol). However, the energy barrier of reductive elimination in the allenyl-type pathway via transition state **TS-3a-Yb** is 37.8 kcal/mol, which is a high energy barrier and difficult to overcome under standard reaction conditions. This is similar to the pathway without Yb(OTf)_3_. The nucleophilic attack in the π-propargyl type pathway is not promoted with the addition of Yb(OTf)_3_ since the energy barrier via transition state **TS-3a′-Yb** is even increased to 23.5 kcal/mol. Hence, the additive Yb(OTf)_3_ plays an important role in promoting the cleavage of the C–C bond and does not influence the following cycloaddition process significantly.

**Scheme 8:**
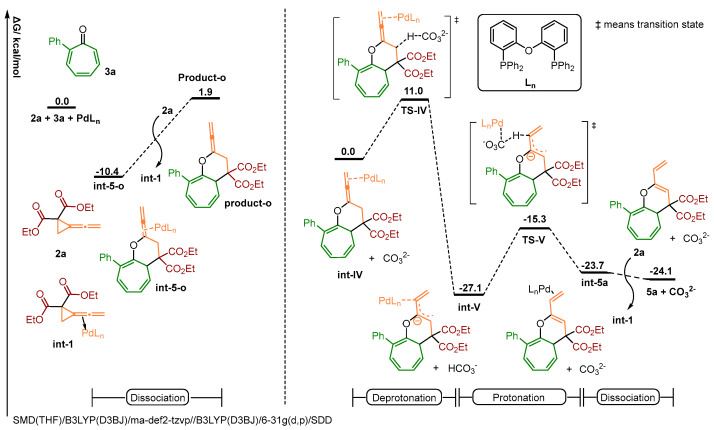
DFT calculation studies of the rearrangement of tropone derivative.

The exothermic ligand exchange step restrains the rearrangement of the tropsulfimide derivative in the cycloaddition part. We have also investigated the ligand exchange step of the tropone derivative. Hypothetically, an allene intermediate **int-IV** was first generated in a similar manner as **int-5** shown in Scheme 8, which is an endothermic process with an 11.9 kcal/mol barrier rather than **int-5** in ligand exchanging to actuate the rearrangement, which can also explain why the allene product of tropone was unable to be detected. Based on such a result, the intermediate **int-IV** was set as the starting point for our investigation. The acidic H is attracted by the carbonate anion through **TS-IV** with an energy barrier of 11.0 kcal/mol to form a formal allylic anion with the bicarbonate anion **int-V**. The bicarbonate part would twist to another side of allylic anion, which makes the hydrogen part close to conjugated diene part, and overwhelms the transition state **TS-V** with 11.8 kcal/mol barrier by proton transfer and ligand exchange to afford the product **5a**. Palladium catalyst coordinated with VDCP diester and carbonate anion as a proton shuttle was released to start a new rearrangement reaction.

**Scheme 9:**
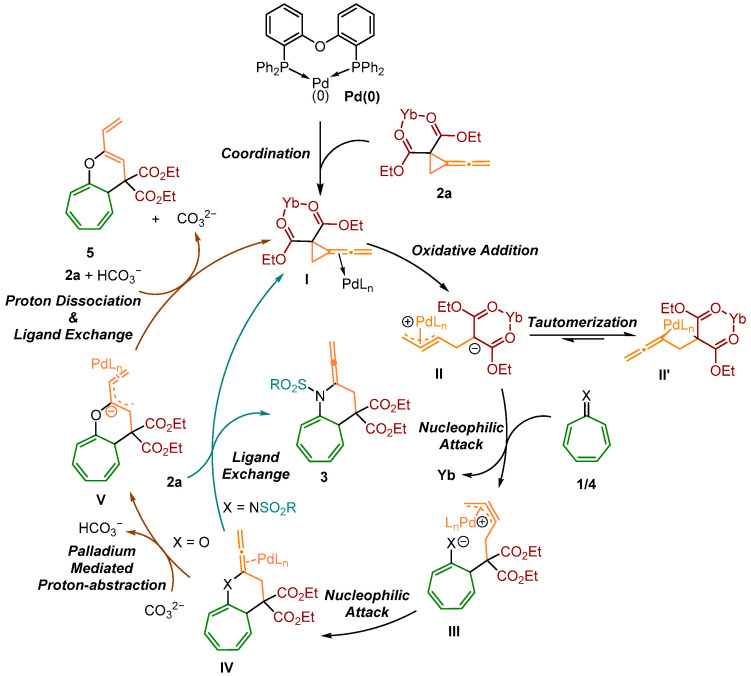
The most plausible reaction mechanism.

According to the former DFT studies, we have already amended the mechanism of the reaction. The most plausible pathway of cycloaddition is summarized in Scheme 9, which starts from π-propargyl-type zwitterionic palladium species **II/II’** generated form **I** and goes through an outer sphere nucleophilic attack to intermediate **III** to form certain allene products **IV**. The key point of rearrangement was found out as the dissociation energy of the ligand exchange step. The allene products of tropsulfimides could dissociate, while tropone derivatives should pass through a palladium-mediated proton transfer conducted by carbonate anion as a proton shuttle through intermediate **V** to form conjugated diene products and regenerate the VDCP-coordinated palladium catalyst.

## 3. Materials and Methods

The preparation of tropsulfimines **1** [24], vinylidenecyclopropane-diester **2** [25], and tropones **4** [26] and products **3** and **5** [19] followed the previous literature procedure. Compound **6a** was detected by HRMS-ESI, and compound **6b** was detected by MALDI.

All calculations were performed with the Gaussian 16 program [27]. The geometries of all minima and transition states have been optimized at the B3LYP(D3BJ)/6-31G(d,p) level of theory for all nonmetallic atoms (H, C, N, O, S, and P) and the SDD level of theory for the metallic atom Pd. The subsequent frequency calculations on the stationary points were carried out at the same level of theory to ascertain the nature of the stationary points as minima or first-order saddle points on the respective potential energy surfaces. All transition states were characterized by one and only one imaginary frequency pertaining to the desired reaction coordinate. Thermochemical corrections to 298.15 K have been calculated for all minima from unscaled vibrational frequencies obtained at this same level. The solvent effect was estimated by the SMD method in THF (ε = 46.826). Solution-phase single-point energy calculations were performed at the B3LYP(D3BJ)/def2-tzvp level for all nonmetallic atoms (H, C, N, O, S, and P) and the SDD level of theory for metallic atom Pd based on the gas-phase-optimized structures. The possible conformers for each species were searched manually, and the best conformer was used to calculate the reaction energy profile.

## 4. Conclusions

In summary, we conducted a series of DFT calculation studies based on our former control experiments, which located the [8 + 3] cycloaddition as a palladium-catalyzed reaction with zwitterionic palladium species. DFT calculation results confirm the position of C–C oxidative addition cleavage and the promotion of Lewis acid additive Yb(OTf)_3_ in oxidative addition. The most plausible mechanism of a [8 + 3] cycloaddition via zwitterionic π-propargyl palladium is proposed based on systematic mechanistic studies, which also demonstrate the dissociation energy as a unique point to regulate the selectivity of rearrangement. Although the experimental part of those studies still has certain flaws due to the instability of zwitterionic π-propargyl palladium species, this mechanistic insight of zwitterionic π-propargyl palladium will provide a deeper understanding of subsequent related studies.

## Data Availability

Data are contained within the article and Supplementary Materials.

## Supplementary Materials

The supporting information can be downloaded at: https://www.mdpi.com/article/10.3390/molecules30010103/s1. Scheme S1: DFT calculation on the reaction mechanism; Scheme S2: DFT calculation on the reaction mechanism with Yb(OTf)_3_; Scheme S3: DFT calculations on the formation of diene product.
