# Supplementary material for: Theoretical Studies on the Reaction Mechanism for the Cycloaddition of Zwitterionic π-Allenyl Palladium Species: Substrate-Controlled Isomerization
Source: Molecules. 2024 Dec 30;30(1):103. doi: 10.3390/molecules30010103 (PMC11722397; doi:10.3390/molecules30010103)
Supplement: Supplementary file 1 [file molecules-30-00103-s001.zip › molecules-3311799-supplementary.pdf]

Theoretical Studies on the Reaction Mechanism for the Cycloaddition of Zwitterionic π-Allenyl Palladium Species: Substrate-Controlled Isomerization

Yongjie Long, Jiahao Shen, Min Shi * and Yin Wei *

State Key Laboratory of Organometallic Chemistry, Center for Excellence in Molecular Synthesis, Shanghai Institute of Organic Chemistry, University of Chinese Academy of Sciences, Chinese Academy of Sciences,
345 Lingling Road, Shanghai 200032, China; longyj@sioc.ac.cn (Y.L.); m18332726812@outlook.com (J.S.)
* Correspondence: mshi@mail.sioc.ac.cn (M.S.); weiyin@sioc.ac.cn (Y.W.)

Table of Contents

(A) General Information S2

(B) General Procedure for the Products Derived from Tropsulfimines S3

(C) General Procedure for the Products Derived from Tropones S3

(D) Control Experiments S3

(E) Detection of VDCPs Dimer and the Zwitterionic π-Allenyl Palladium Species S5

(F) Characterization Data of Products S9

(G) Spectroscopic Data of Products S10

(H) Computational Details S12

(I) References S77

(A) General Information

Proton nuclear magnetic resonance (^{1}H NMR) spectra and carbon nuclear magnetic resonance (^{13}C NMR) spectra were recorded at 400 MHz, 100 MHz, respectively. ^{1}H NMR spectrum uses TMS (δ = 0.00 ppm) as internal standard, ^{13}C NMR spectrum uses $CDCl_3$ (δ = 77.00 ppm) as internal standard. High Resolution Mass Spectra (HRMS) were recorded by ESI and MALDI method. The employed solvents were dry up by standard methods when necessary. Commercially obtained reagents were used without further purification. Petroleum ether refers to the fraction with boiling point in the range 60-90 ºC. For thin-layer chromatography (TLC), silica gel plates (Huanghai GF254) were used. Column chromatography was carried out using 300-400 mesh silica gel at increased pressure.

The preparation of tropsulfimines **1** [1]. vinylidenecyclopropane-diester **2** [2] and tropones **4** [3] and product **3** and **5** [4] followed the previous literature procedure.

(B)General Procedure for the Products Derived from Tropsulfimines

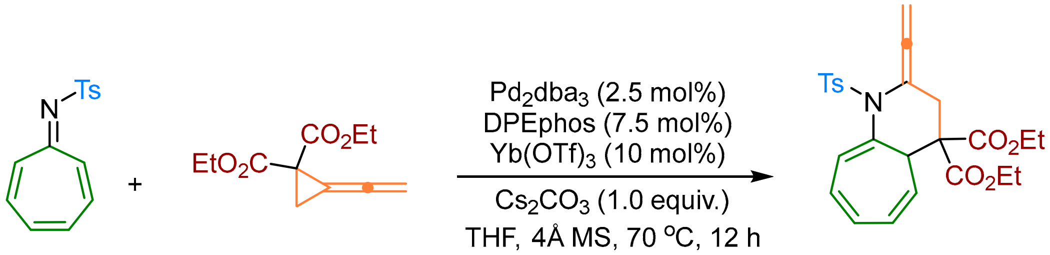

1a **2a** **3a**

Tris(dibenzylideneacetone)dipalladium (0.002 mmol, 2.5 mol%) and DPEPhos (0.007 mmol, 7.5 mol%) was added to an oven dried 10 mL sealed tube equipped with a magnetic stirring bar under argon atmosphere. The resulting catalyst system was stirred in THF (degassed) (1.0 mL) for 30 minutes. After that, **1a** (0.1 mmol), **2a** (0.2 mmol), $Yb(OTf)_3$ (0.01 mmol, 10 mol%) and Cs_2CO_3 (0.1 mmol, 1.0 equiv.) were added under argon atmosphere. The reaction mixture was stirred at 70 °C for 12 h. The reaction solution was filtered through a pad of celite to remove insoluble solids. Then, the solvent was evaporated under vacuum and the residue was purified by a column chromatography to afford the desired product **3a**.

(C) General Procedure for the Products Derived from Tropones

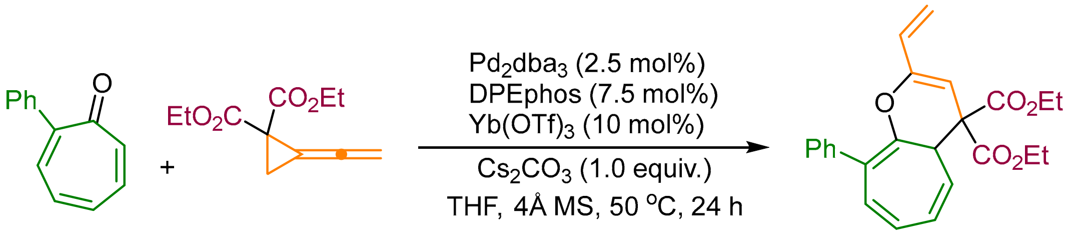

4a **2a** **5a**

Tris(dibenzylideneacetone)dipalladium (0.002 mmol, 2.5 mol %) and DPEPhos (0.007 mmol, 7.5 mol %) was added to an oven dried 10 mL sealed tube equipped with a magnetic stirring bar under argon atmosphere. The resulting catalyst system was stirred in THF (degassed) (1.0 mL) for 30 min. After that, **4a** (0.1 mmol), **2a** (0.2 mmol), $Yb(OTf)_3$ (0.01 mmol, 10 mol%) and Cs_2CO_3 (0.1 mmol, 1.0 equiv.) were added under argon atmosphere. The reaction mixture was stirred at 50 °C for 24 h. The reaction solution was filtered through a pad of celite to remove insoluble solids. Then, the solvent was evaporated under vacuum and the residue was purified by a column chromatography to afford the desired product **5a**.

(D) Control Experiments

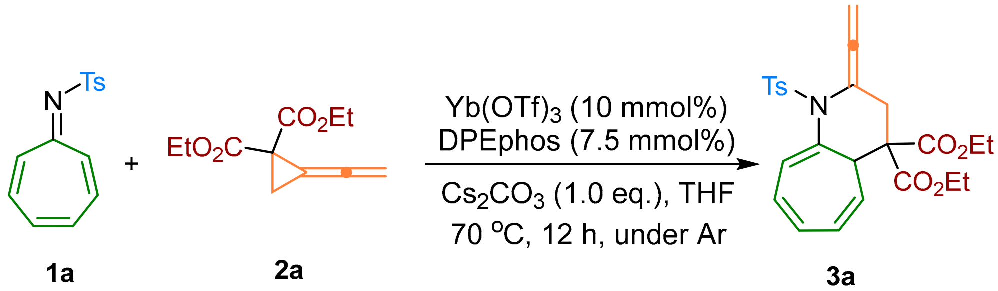

DPEPhos (0.004 g, 0.007 mmol, 7.5 mol%) was added to an oven dried 10 mL sealed tube equipped with a magnetic stirring bar under argon atmosphere. The resulting catalyst system was stirred in THF (degassed) (1.0 mL) for 30 min. After that, **1a** (0.026 g, 0.1 mmol), **2a** (35 µL, 0.2 mmol), $Yb(OTf)_3$ (0.006 g, 0.01 mmol, 10 mol %) and Cs_2CO_3 (0.033 g, 0.1 mmol, 1.0 equiv.) were added under argon atmosphere. The reaction mixture was stirred at 70 °C for 12 h. None of the desired product **3a** was generated through TLC detection.

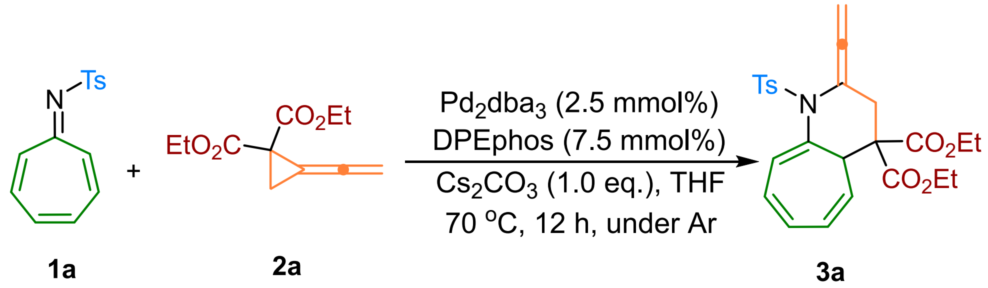

Tris(dibenzylideneacetone)dipalladium (0.002 g, 0.002 mmol, 2.5 mol%) and DPEPhos (0.004 g, 0.007 mmol, 7.5 mol%) was added to an oven dried 10 mL sealed tube equipped with a magnetic stirring bar under argon atmosphere. The resulting catalyst system was stirred in THF (degassed) (1.0 mL) for 30 min. After that, **1a** (0.026 g, 0.1 mmol), **2a** (35 µL, 0.2 mmol) and Cs_2CO_3 (0.033 g, 0.1 mmol, 1.0 equiv.) were added under argon atmosphere. The reaction mixture was stirred at 70 °C for 12 h. The reaction solution was filtered through a pad of celite to remove insoluble solids. Then, the solvent was evaporated under vacuum and **3a** was afforded by a column chromatography in the yield of 36%.

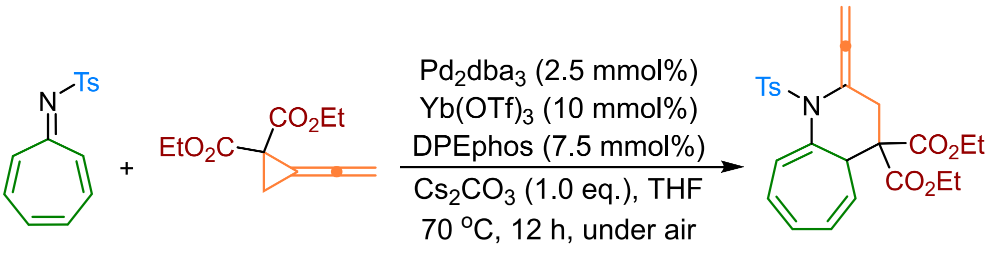

Tris(dibenzylideneacetone)dipalladium (0.002 g, 0.002 mmol, 2.5 mol%) and DPEPhos (0.004 g, 0.007 mmol, 7.5 mol%) was added to an oven dried 10 mL sealed tube equipped with a magnetic stirring bar under air. The resulting catalyst was stirred in THF (1.0 mL) for 30 min. After that, **1a** (0.026 g, 0.1 mmol), **2a** (35 μL, 0.2 mmol), $Yb(OTf)_3$ (0.006 g, 0.01 mmol, 10 mol%) and Cs_2CO_3 (0.033 g, 0.1 mmol, 1.0 equiv.) were added under air. The reaction mixture was stirred at 70 °C for 12 h. None of the product **3a** was generated through TLC detection.

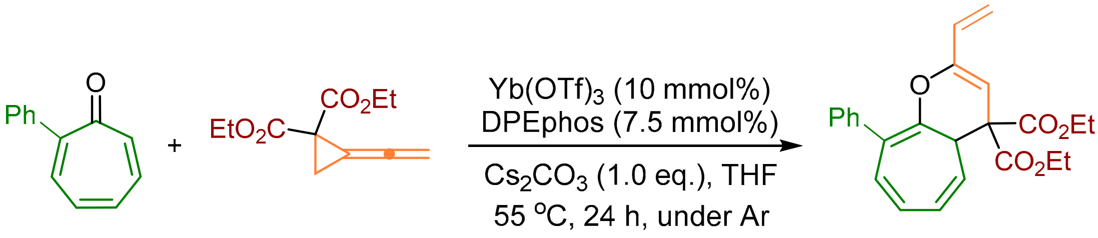

DPEPhos (0.004 g, 0.007 mmol, 7.5 mol%) was added to an oven dried 10 mL sealed tube equipped with a magnetic stirring bar under argon atmosphere. The resulting catalyst system was stirred in THF (degassed) (1.0 mL) for 30 min. After that, **4a** (0.018 g, 0.1 mmol), **2a** (35 μL, 0.2 mmol), $Yb(OTf)_3$ (0.006 g, 0.01 mmol, 10 mol %) and Cs_2CO_3 (0.033 g, 0.1 mmol, 1.0 equiv.) were added under argon atmosphere. The reaction mixture was stirred at 55 °C for 24 h. None of the desired product **5a** was generated through TLC detection.

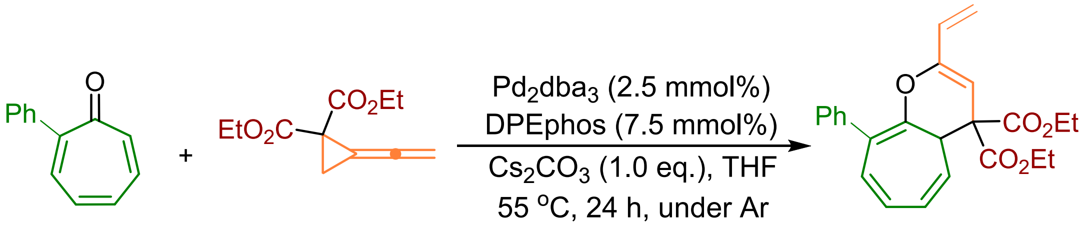

Tris(dibenzylideneacetone)dipalladium (0.002 g, 0.002 mmol, 2.5 mol%) and DPEPhos (0.004 g, 0.007 mmol, 7.5 mol%) was added to an oven dried 10 mL sealed tube equipped with a magnetic stirring bar under argon atmosphere. The resulting catalyst system was stirred in THF (degassed) (1.0

mL) for 30 min. After that, **4a** (0.018 g, 0.1 mmol), **2a** (35 μL, 0.2 mmol) and Cs_2CO_3 (0.033 g, 0.1 mmol, 1.0 equiv.) were added under argon atmosphere. The reaction mixture was stirred at 55 °C for 24 h. The reaction solution was filtered through a pad of celite to remove insoluble solids. Then, the solvent was evaporated under vacuum and **5a** was afforded by a column chromatography in the yield of 23%.

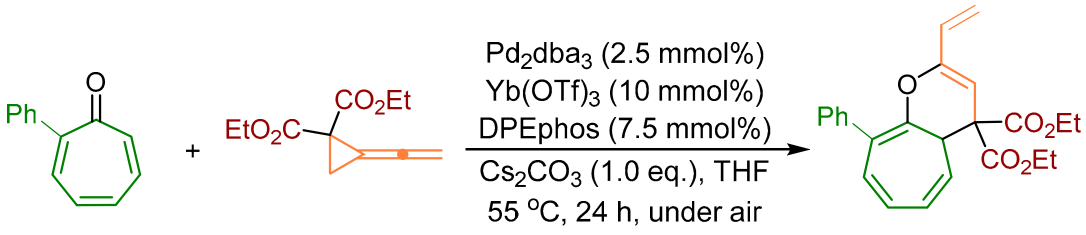

4a **2a** **5a**

Tris(dibenzylideneacetone)dipalladium (0.002 g, 0.002 mmol, 2.5 mol%) and DPEPhos (0.004 g, 0.007 mmol, 7.5 mol%) was added to an oven dried 10 mL sealed tube equipped with a magnetic stirring bar under air. The resulting catalyst was stirred in THF (1.0 mL) for 30 min. After that, **4a** (0.018 g, 0.1 mmol), **2a** (35 μL, 0.2 mmol), $Yb(OTf)_3$ (0.006 g, 0.01 mmol, 10 mol%) and Cs_2CO_3 (0.033 g, 0.1 mmol, 1.0 equiv.) were added under air. The reaction mixture was stirred at 55 °C for 24 h. None of the product **5a** was generated through TLC detection.

(E) Detection of VDCPs Dimer and the Zwitterionic π-Allenyl Palladium Species

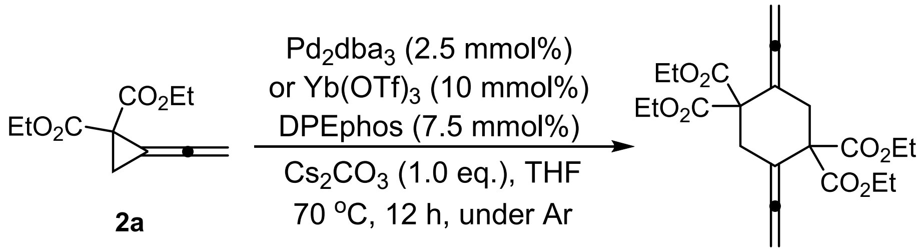

6a

Tris(dibenzylideneacetone)dipalladium (0.002 g, 0.002 mmol, 2.5 mol%) and DPEPhos (0.004 g, 0.007 mmol, 7.5 mol%) was added to an oven dried 10 mL sealed tube equipped with a magnetic stirring bar under argon atmosphere. The resulting catalyst system was stirred in THF (degassed) (1.0 mL) for 30 min. After that, **2a** (35 μL, 0.2 mmol), $Yb(OTf)_3$ (0.006 g, 0.01 mmol, 10 mol%) and Cs_2CO_3 (0.033 g, 0.1 mmol, 1.0 equiv.) were added under argon atmosphere. The reaction mixture was stirred at 70 °C for 4 h. The reaction solution was filtered through a pad of celite to remove insoluble solids. The intermediate **6a** was detected through HRMS (ESI-TOF) m/z: $[M+Na]^+$ Calcd for $C_{22}H_{28}O_8Na$ 443.1676; found 443.1674.

Spectrum

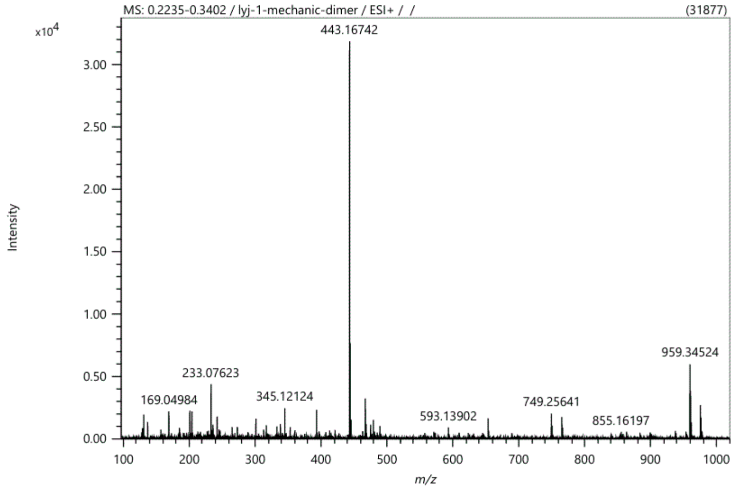

Elemental Composition

Parameters	
Tolerance:	±5.00 ppm
Electron:	Odd/Even
Charge:	+1
DBE:	-1.5 - 200.0

Elements Set 1:

Symbol	C	H	N	O	Na	S	Cl	Br
Min	0	0	0	0	1	0	0	0
Max	200	200	3	8	1	0	0	0

Symbol	Si	F	B
Min	0	0	0
Max	0	0	0

Results

Mass	Intensity	Intensity [%]	Formula	Calculated Mass	Mass Difference [mDa]	Mass Difference [ppm]	DBE
443.16742	31876.56	100.00	C22 H28 O8 Na	443.16764	-0.22	-0.49	8.5
			C20 H26 N3 O7 Na	443.16630	1.13	2.54	9.0

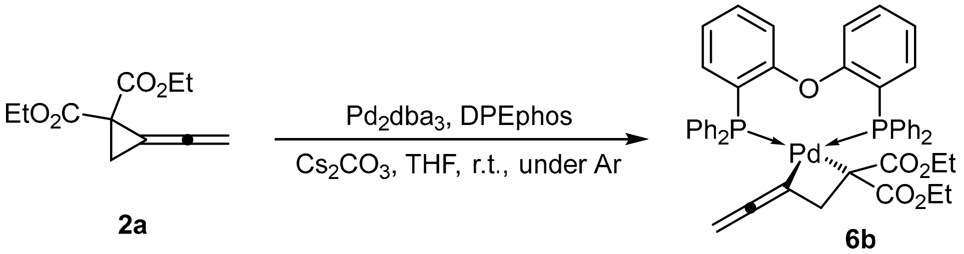

Tris(dibenzylideneacetone)dipalladium (0.046 g, 0.05 mmol) and DPEPhos (0.054 g, 0.1 mmol) was added to an oven dried 10 mL sealed tube equipped with a magnetic stirring bar in glovebox under argon atmosphere. The resulting catalyst system was stirred in THF (degassed) (2.0 mL) for 30 min in a glovebox. After that, **2a** (18 μL, 0.1 mmol) and Cs_2CO_3 (0.033 g, 0.1 mmol) were added in glovebox under argon atmosphere. The solution was moved to a sealed bottle for the detection, while its color turned from yellow to pale green. The intermediate **6b** was detected through MS (MALDI).

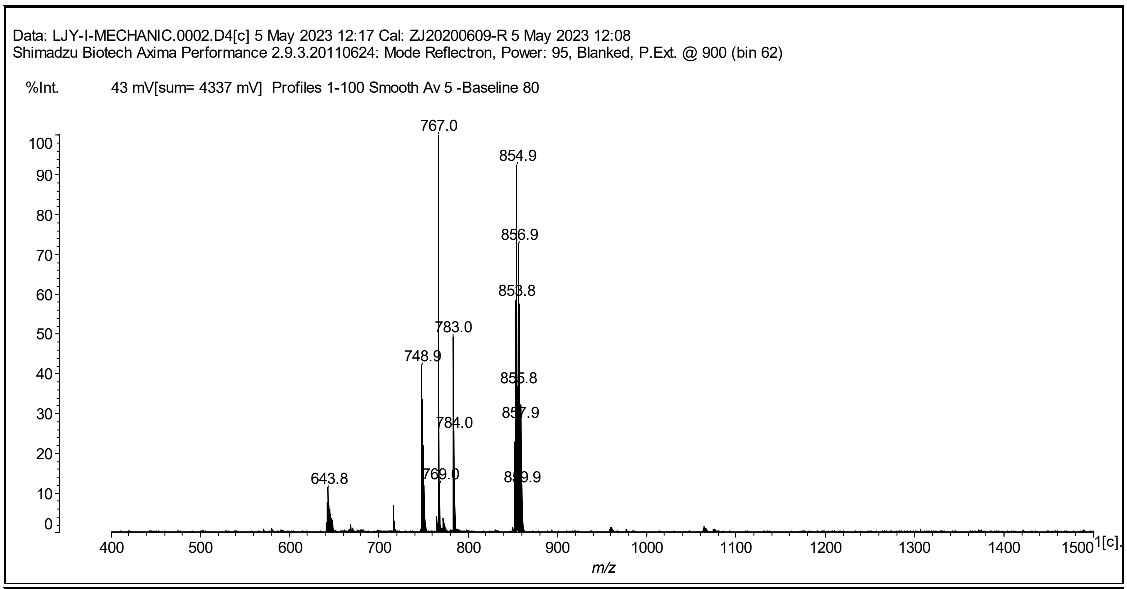

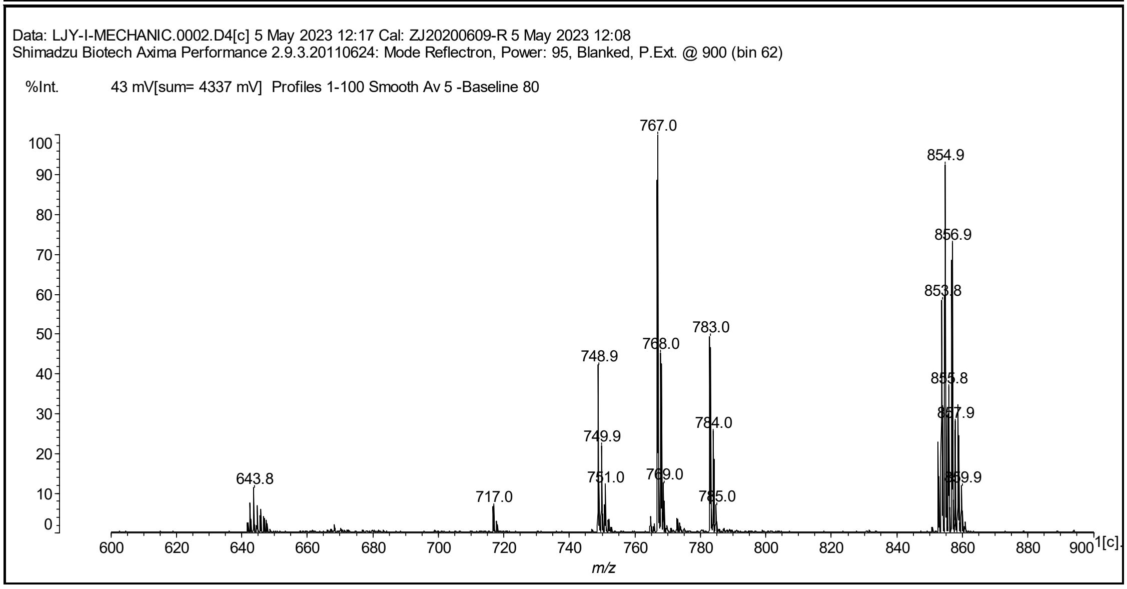

(F) Characterization Data of Products

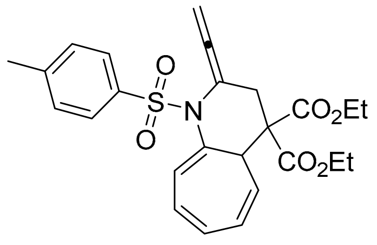

Compound 3a: a colorless solid; Mp: 123-127 °C; Eluent: PE/EA = 4:1. ^{1}H NMR (400 MHz, $CDCl_3$, TMS) δ 7.27 (d, *J* = 8.0 Hz, 2H), 7.15 (d, *J* = 8.0 Hz, 2H), 6.84 (dd, *J* = 10.7, 6.0 Hz, 1H), 6.71 (d, *J* = 6.5 Hz, 1H), 6.63 (dd, *J* = 10.8, 5.8 Hz, 1H), 6.08 (dd, *J* = 8.8, 5.8 Hz, 1H), 5.28 (dd, *J* = 11.5, 4.2 Hz, 1H), 5.14 (dd, *J* = 11.5, 4.0 Hz, 1H), 4.23 – 4.06 (m, 5H), 2.75 (d, *J* = 13.9 Hz, 1H), 2.39 (s, 3H), 2.29 (dt, *J* = 13.8, 4.2 Hz, 1H), 1.71 (d, *J* = 7.6 Hz, 1H), 1.22 (t, *J* = 7.1 Hz, 3H), 1.12 (t, *J* = 7.1 Hz, 3H); ^{13}C NMR (100 MHz, $CDCl_3$, TMS) δ 207.6, 169.3, 168.8, 144.1, 134.2, 129.4, 129.1, 128.2, 127.2, 126.5, 117.0, 114.3, 113.9, 102.1, 81.2, 61.9, 61.7, 55.9, 40.4, 30.5, 21.6, 14.1, 14.0; IR (neat): ν 2983, 2923, 1968, 1728, 1587, 1493, 1443, 1365, 1253, 1164, 1133, 1090, 872, 713, 657 cm^{-1}; HRMS (ESI-TOF) m/z: $[M+Na]^+$ Calcd for $C_{25}H_{27}NO_6NaS$ 492.1451; found 492.1444.

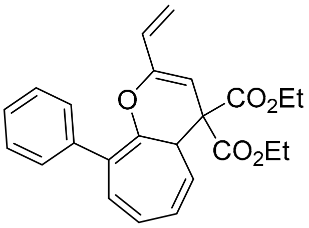

Compound 5a: a colorless solid; Mp: 104-106 °C; Eluent: PE/EA = 10:1. ^{1}H NMR (400 MHz, $CDCl_3$, TMS) δ 7.47 (d, *J* = 7.6 Hz, 2H), 7.35 (t, *J* = 7.6 Hz, 2H), 7.25 (t, *J* = 7.2 Hz, 1H), 6.89 (d, *J* = 11.0 Hz, 1H), 6.81 (dd, *J* = 11.0, 5.2 Hz, 1H), 6.35 (dd, *J* = 8.8, 5.4 Hz, 1H), 6.13 (dd, *J* = 17.2, 11.0 Hz, 1H), 5.65 (s, 1H), 5.31 (d, *J* = 17.2 Hz, 1H), 5.18 (dd, *J* = 8.8, 6.8 Hz, 1H), 5.08 (d, *J* = 11.0 Hz, 1H), 4.30 (q, *J* = 7.1 Hz, 2H), 4.26 – 4.07 (m, 2H), 2.33 (d, *J* = 6.8 Hz, 1H), 1.28 – 1.22 (m, 6H); ^{13}C NMR (100 MHz, $CDCl_3$, TMS) δ 168.7, 168.5, 150.4, 137.3, 134.8, 131.5, 130.4, 129.4, 128.2, 127.7, 126.6, 126.3, 116.9, 116.8, 116.7, 98.7, 62.1, 61.9, 53.6, 39.7, 14.1, 14.0; IR (neat): ν 2979, 2926, 1733, 1659, 1597, 1488, 1364, 1334, 1250, 1206, 1092, 830 cm^{-1}; HRMS (ESI-TOF) m/z: $[M+Na]^+$ Calcd for $C_{24}H_{24}O_5Na$ 415.1516; found 415.1514.

(G) Spectroscopic Data of Substrates and Products

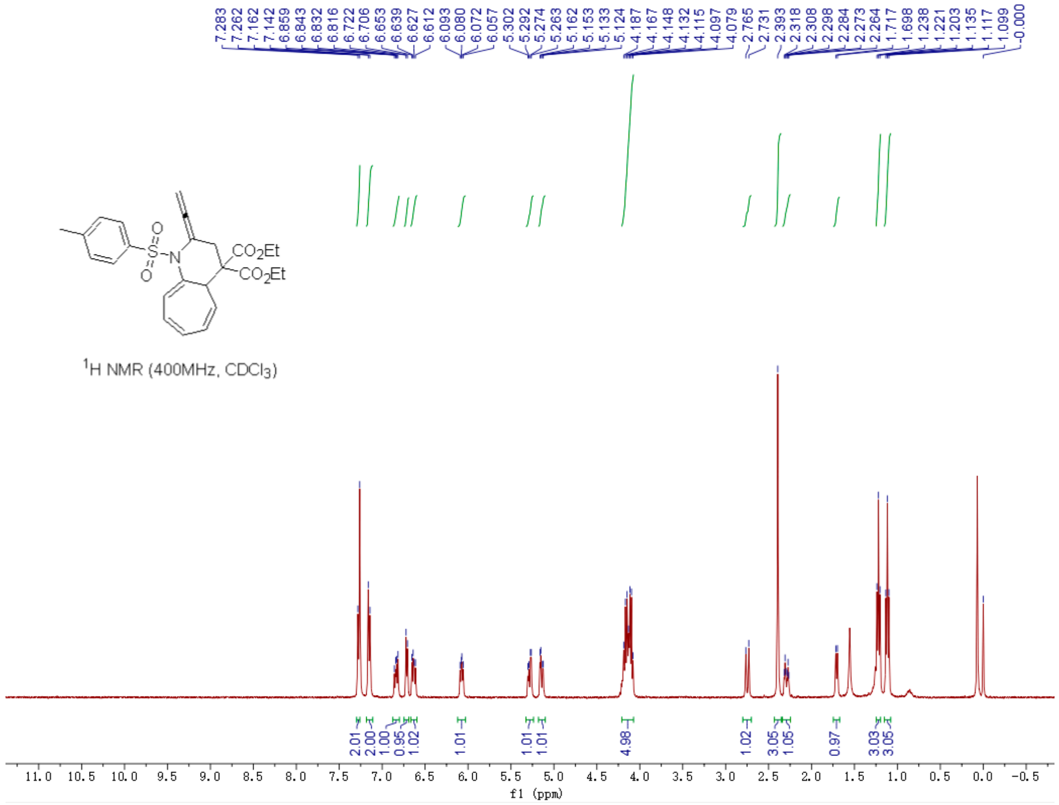

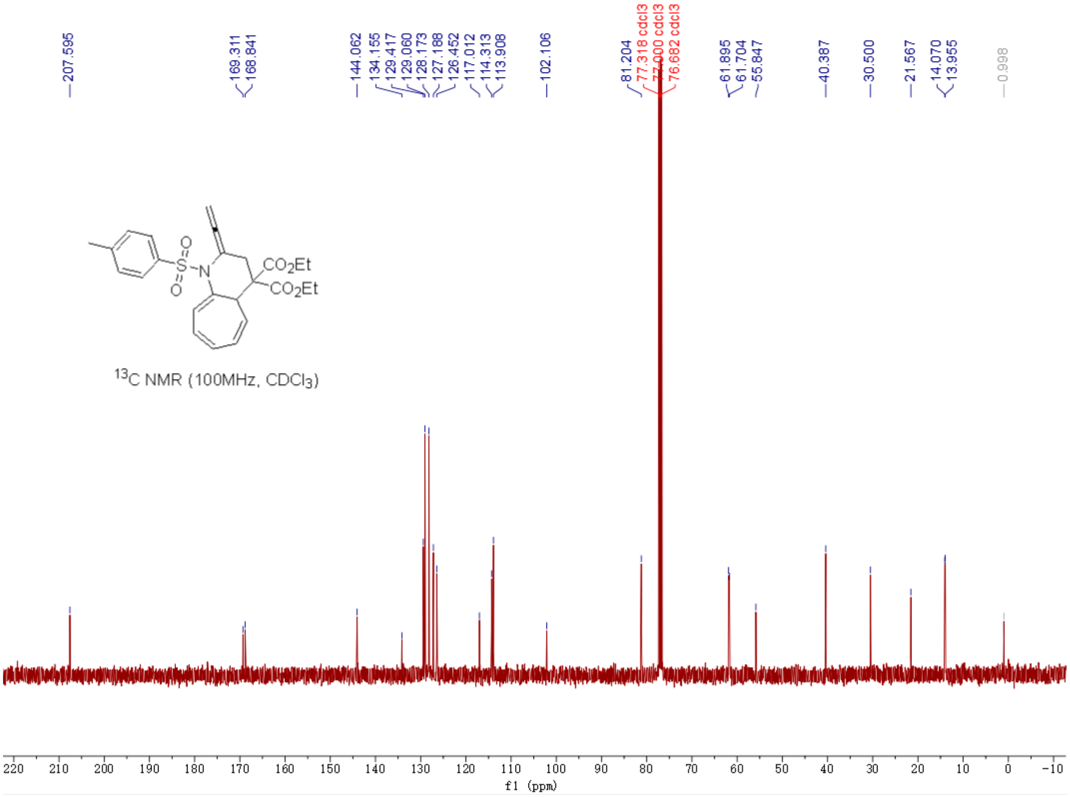

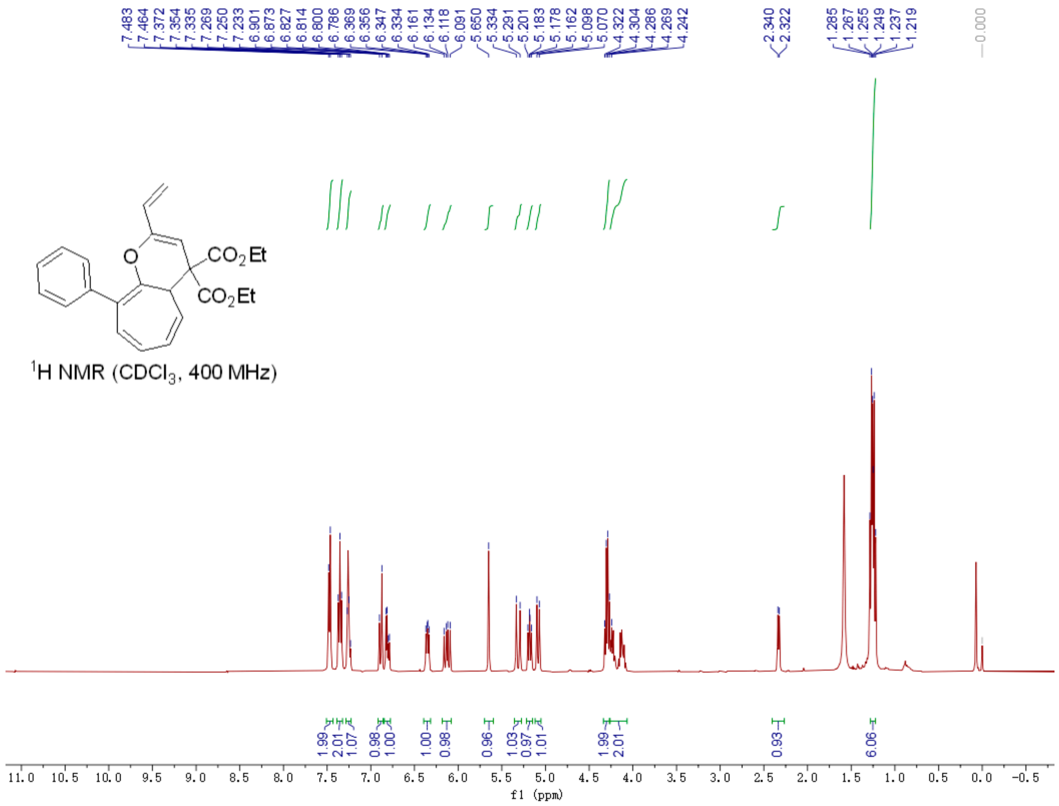
CO2Et
CO2Et
O
1H NMR (CDCl3, 400 MHz)
7.483
7.464
7.372
7.354
7.335
7.269
7.250
7.233
6.901
6.873
6.827
6.814
6.800
6.786
6.369
6.356
6.347
6.334
6.161
6.134
6.118
6.091
5.650
5.334
5.291
5.201
5.183
5.178
5.162
5.098
5.070
4.322
4.304
4.286
4.269
4.242
2.340
2.322
1.285
1.267
1.255
1.249
1.237
1.219
0.000
1.99
2.01
1.07
0.98
1.00
1.00
0.98
0.96
1.03
0.97
1.01
1.99
2.01
0.93
6.06
f1 (ppm)

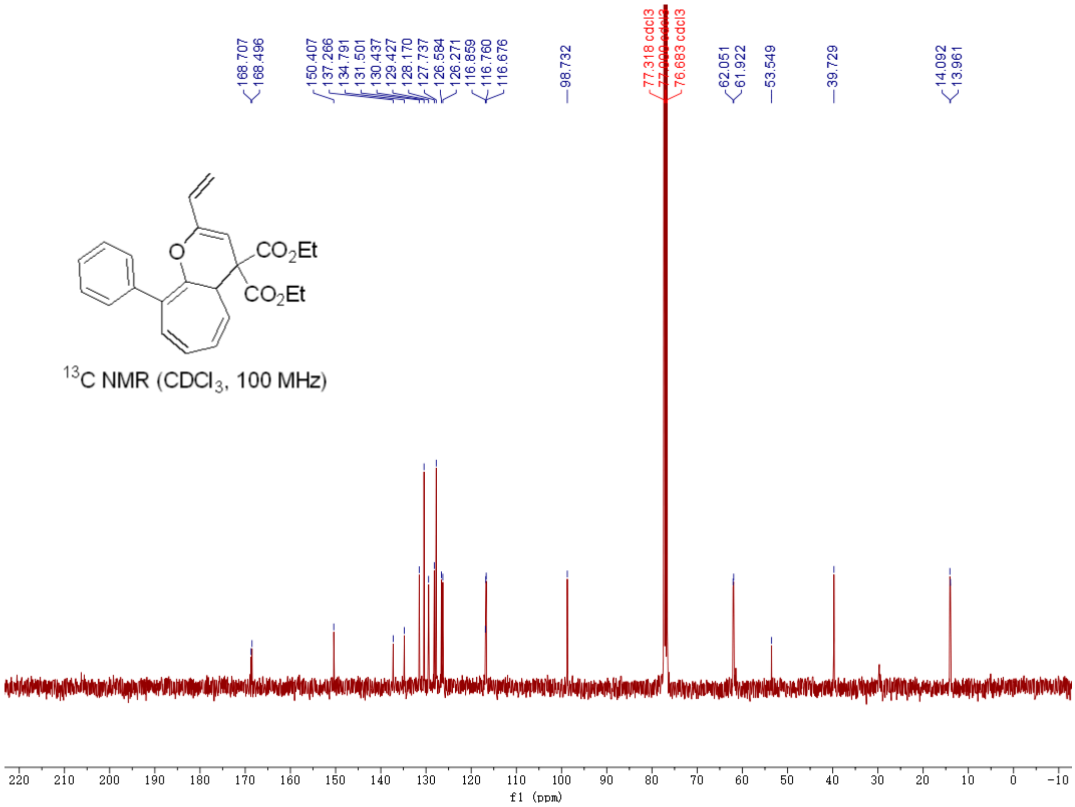
CO2Et
CO2Et
O
13C NMR (CDCl3, 100 MHz)
168.707
168.496
150.407
137.266
134.791
131.501
130.437
129.427
128.170
127.737
126.584
126.271
116.859
116.760
116.676
98.732
77.318 cdcl3
76.683 cdcl3
62.051
61.922
53.549
39.729
14.092
13.961
f1 (ppm)

(H) Computational Details

Computational methods

All DFT calculations were performed with Gaussian 16 program [5]. The geometries of all minima and transition states have been optimized at B3LYP-D3BJ/6-31G(d,p) level of theory for all nonmetallic atom (H, C, N, O, S and P), SDD level of theory for metallic atom Pd, and SDD level of theory using 59 electrons in the effective core for metallic atom Yb. The subsequent frequency calculations on the stationary points were carried out at the same level of theory to ascertain the nature of the stationary points as minima or first-order saddle points on the respective potential energy surfaces. All transition states were characterized by one and only one imaginary frequency pertaining to the desired reaction coordinate. Thermochemical corrections to 298.15 K have been calculated for all minima from unscaled vibrational frequencies obtained at this same level. The solvent effect was estimated by the SMD method in THF (ε = 46.826). Solution-phase single point energy calculations were performed at B3LYP-D3BJ/ma-def2tzvp level for all nonmetallic atom (H, C, N, O, S and P) and SDD level of theory for metallic atom Pd based on the gas phase optimized structures based on the gas phase optimized structures. The solution-phase single point energy calculations with Yb were performed at B3LYP-D3BJ/ def2tzvp level for all nonmetallic atom (H, C, N, O, S and P), SDD level of theory for metallic atom Pd, and SDD level of theory using 59 electrons in the effective core for metallic atom Yb based on the gas phase optimized structures. The possible conformers for each species were searched manually, and the best conformer was used to calculate the reaction energy profile.

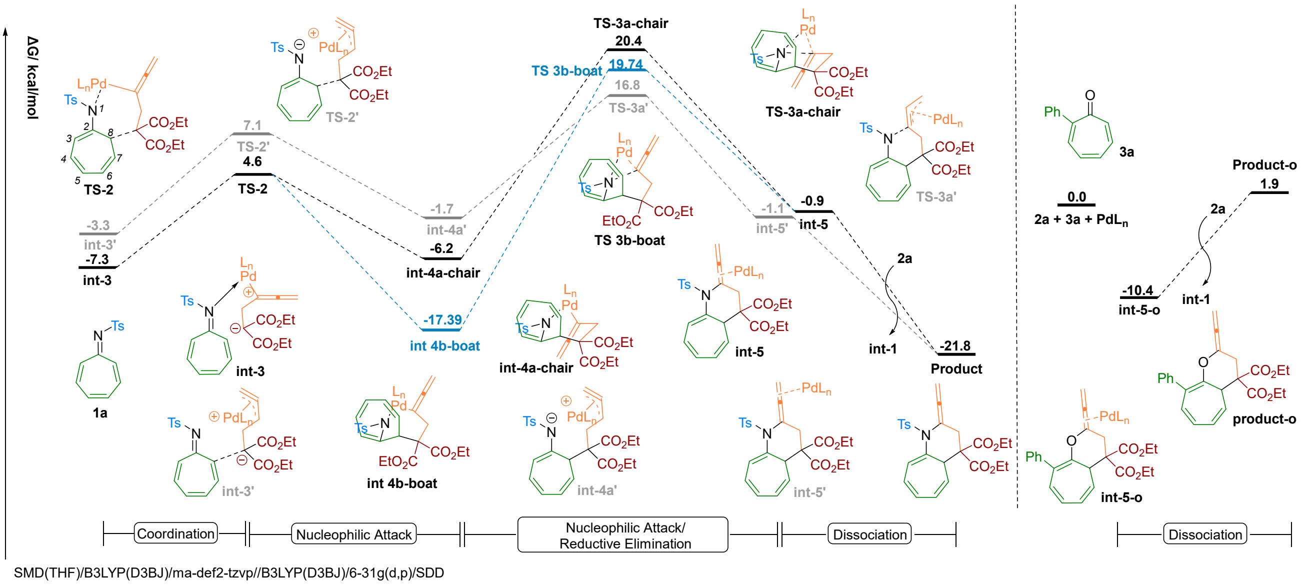

Scheme S1. DFT calculation on the reaction mechanism

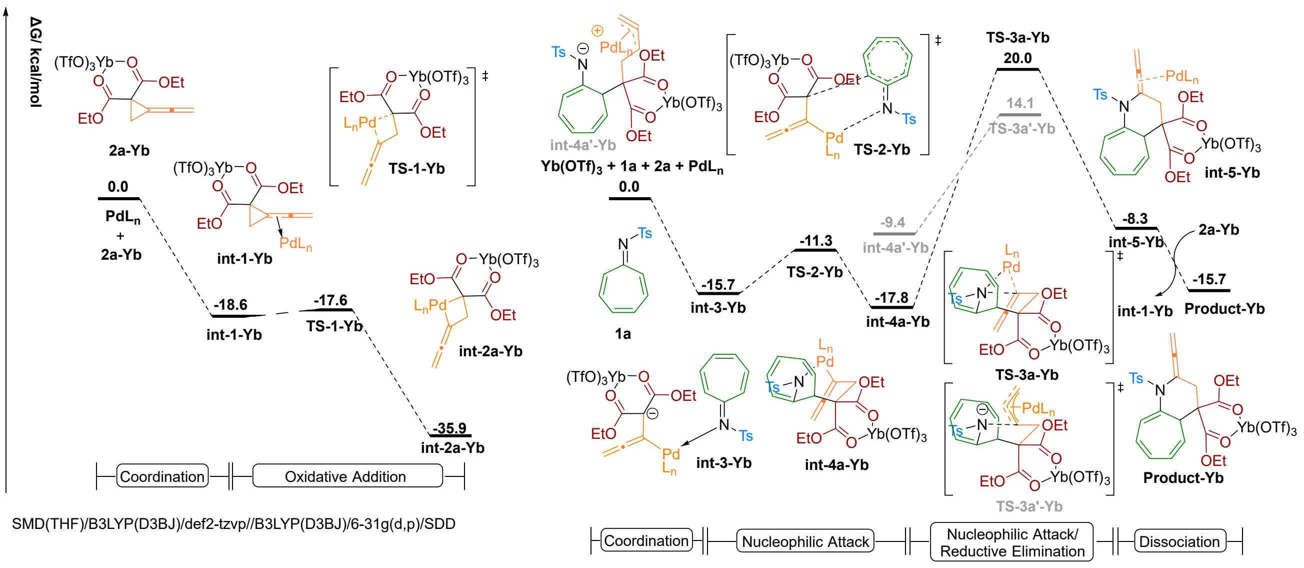

Scheme S2. DFT calculation on the reaction mechanism with $Yb(OTf)_3$

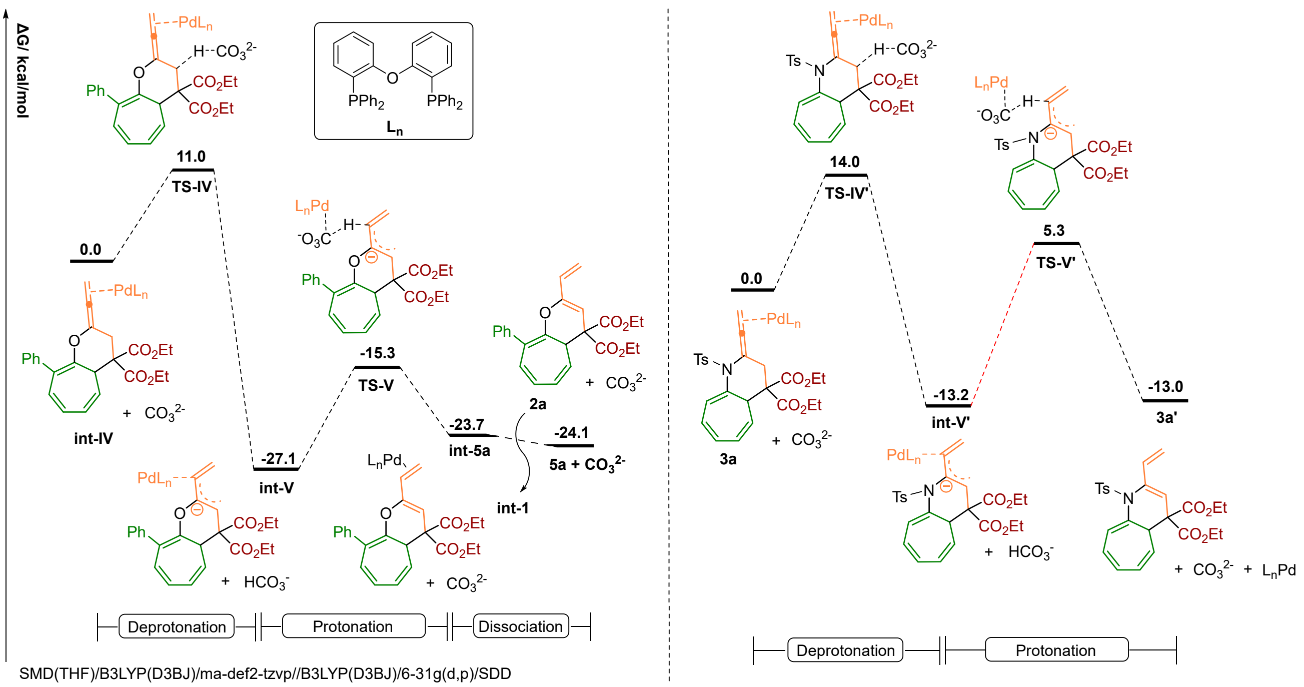

Scheme S3. DFT calculations on the formation of diene product

Table S1.[a]

	G_{298}	E_{tot} (E_h)	$E_{tot, THF}$ (E_h)
**1a**	-728.293025	-728.478677	-728.772168
**PdDPE**	-2274.356111	-2274.820264	-2275.464017
**1b**	-1144.494555	-1144.687642	-1145.053822
**int-1**	-3002.68327	-3003.363411	-3004.289035
**TS-1**	-3002.645706	-3003.325504	-3004.249285
**int-2a**	-3002.688406	-3003.371298	-3004.277618
**int-2b**	-3002.656652	-3003.342003	-3004.295787
**TS-1'**	-3002.635623	-3003.313631	-3004.239696
**int-2a'**	-3002.68517	-3003.36567	-3004.296413
**TS-1"**	-3002.634204	-3003.311544	-3004.242182
**int-2a"**	-3002.676667	-3003.354129	-3004.28688
**int-3**	-4147.172785	-4148.082785	-4149.368675
**TS-2**	-4147.14466	-4148.05273	-4149.347797
**int-4b-boat**	-4147.181344	-4148.091745	-4149.384369
**TS-3b-boat**	-4147.128571	-4148.035648	-4149.321155
**int-5**	-4147.164497	-4148.074494	-4149.355631
**product-boat**	-1872.820317	-1873.231592	-1873.869196
**int-4a-chair**	-4147.165878	-4148.075456	-4149.366535
**TS-3a-chair**	-4147.125708	-4148.03202	-4149.320966
**product-chair**	-1872.820136	-1873.234682	-1873.869155
**int-3'**	-4147.151971	-4148.055623	-4149.355207
**TS-2'**	-4147.138289	-4148.045869	-4149.342403
**int-4a'**	-4147.152089	-4148.056578	-4149.353043
**TS-3a'**	-4147.132462	-4148.040752	-4149.328666
**int-5'**	-4147.164402	-4148.074518	-4149.356832
**3a**	-576.504669	-576.658659	-576.876367
**int-5-o**	-3579.190012	-3580.054165	-3581.189512
**product-o**	-1304.811034	-1305.182132	-1305.671001
**2a-Yb**[b]	-3652.302786	-3652.540323	-3653.872199
**int-1-Yb**[b]	-5926.717489	-5927.454265	-5929.366992
**TS-1-Yb**[b]	-5926.716699	-5927.453315	-5929.36522
**int-2a-Yb**[b]	-5926.73881	-5927.474784	-5929.393707
**int-3-Yb**[b]	-7071.212292	-7072.171738	-7074.443707
**TS-2-Yb**[b]	-7071.208468	-7072.172382	-7074.441183
**int-4a-Yb**[b]	-7071.221193	-7072.185415	-7074.451843

**TS-3a-Yb**[b]	-7071.160771	-7072.122898	-7074.389501
**int-5-Yb**[b]	-7071.20951	-7072.171723	-7074.434668
**int-4a'-Yb**[b]	-7071.200164	-7072.160194	-7074.434141
**TS-3a'-Yb**[b]	-7071.171255	-7072.134391	-7074.399926
**product-Yb**[b]	-4796.812904	-4797.276141	-4798.965079
**int-IV+CO_3^{2-}**	-3842.955239	-3843.825578	-3845.267592
**TS-IV**	-3842.940361	-3843.805882	-3845.245204
**int-V**	-3842.979351	-3843.848777	-3845.309807
**TS-V**	-3842.963917	-3843.830004	-3845.287758
**5a+PdL_n+CO_3^{2-}**	-3842.973894	-3843.845525	-3845.306621
**5a**	-1304.842779	-1305.215697	-1305.707224
**CO_3^{2-}**	-263.600824	-263.589856	-264.105823
**3a+CO_3^{2-}**	-4410.962898	-4411.873616	-4413.46274
**TS-IV'**	-4410.940875	-4411.845892	-4413.434744
**int-V'**	-4410.991569	-4411.908317	-4413.489784
**TS-V'**	-4410.942457	-4411.852879	-4413.454061
**3a'+PdL_n+CO_3^{2-}**	-4410.960499	-4411.871928	-4413.484134
**int-IV-inner**	-3842.982245	-3843.859228	-3845.294166
**int-V-inner**	-3842.966853	-3843.835202	-3845.278358

a. Calculated at SMD(THF)/B3LYP-D3BJ/ma-def2TZVP/SDD//B3LYP-D3BJ/6-31G(d,p)/SDD

b. Calculated at SMD(THF)/B3LYP-D3BJ/def2TZVP/SDD//B3LYP-D3BJ/6-31G(d,p)/SDD

Archive Entries

1a

\0,1\C,0,-0.03472,-0.099831,0.209265\C,0,-0.07114,0.57296,1.5995\C,0,0.128408,1.3
7827,0.399287\H,0,0.790051,0.387998,2.236042\H,0,-1.036485,0.598571,2.094628\C,0,
0.286663,2.501191,-0.213613\C,0,0.453916,3.635283,-0.84344\H,0,1.447093,4.029149,
-1.044181\H,0,-0.392547,4.22229,-1.19079\C,0,1.16053,-0.961147,-0.097948\C,0,-1.3
13408,-0.523569,-0.465532\O,0,-2.385636,-0.339096,0.332617\O,0,2.312022,-0.312241,
0.171093\O,0,1.098908,-2.107904,-0.476641\O,0,-1.375658,-0.922526,-1.604714\C,0,-
3.667307,-0.612037,-0.279685\C,0,-4.733197,-0.303626,0.75125\H,0,-3.686776,-1.658
701,-0.59677\H,0,-3.765562,0.006171,-1.177049\H,0,-4.607772,-0.928129,1.640116\H,
0,-5.724209,-0.498192,0.33059\H,0,-4.688267,0.745548,1.056594\C,0,3.51874,-1.0892
1,-0.005445\C,0,4.685995,-0.189151,0.343325\H,0,3.560186,-1.439779,-1.04067\H,0,3.
467835,-1.972029,0.639318\H,0,4.708435,0.687752,-0.30932\H,0,5.625971,-0.735519,0.
221447\H,0,4.618093,0.154202,1.379408\\Version=ES64L-G16RevA.03\State=1-A\HF=-728.
7721679\RMSD=7.822e-09\Dipole=0.0839864,1.1571883,1.2355092\Quadrupole=13.6449944,
-6.7168117,-6.9281828,-0.366582,-0.8695644,-5.639014\PG=C01 [X(C11H14O4)]\

PdDPE

\0,1\O,0,-0.141125,-1.383225,1.194383\C,0,1.147438,-1.85066,1.388634\C,0,-0.58102
2,-0.230082,1.809191\C,0,2.228959,-1.344216,0.640386\C,0,1.310866,-2.90663,2.2823
3\C,0,2.568318,-3.481403,2.461712\C,0,3.482635,-1.942249,0.839764\C,0,3.656364,-2.
995198,1.738386\H,0,4.333775,-1.578807,0.275125\H,0,4.640517,-3.435096,1.867075\H,
0,2.692464,-4.304407,3.158584\H,0,0.438862,-3.266801,2.817854\C,0,-1.629495,0.449
646,1.159606\C,0,-0.041875,0.231066,3.005706\C,0,-0.561049,1.388822,3.586175\C,0,
-2.146507,1.594065,1.777159\C,0,-1.61931,2.064152,2.981378\H,0,-2.95392,2.134576,
1.296047\H,0,-2.026834,2.961828,3.435558\H,0,0.784,-0.30137,3.462859\H,0,-0.1299,
1.759983,4.51047\P,0,1.984841,0.054597,-0.554658\P,0,-2.082865,-0.076355,-0.55164
6\C,0,2.056829,1.481653,0.611419\C,0,3.620462,0.095615,-1.403449\C,0,-2.957113,-1.
668385,-0.278477\C,0,-3.45506,1.084925,-0.948119\C,0,-3.495845,-2.035299,0.961748
\C,0,-3.083404,-2.544212,-1.364817\C,0,-3.753141,-3.758401,-1.21947\C,0,-4.159268,
-3.253779,1.108566\C,0,-4.291701,-4.115578,0.018317\H,0,-3.389071,-1.367865,1.811
194\H,0,-2.643159,-2.270886,-2.319924\H,0,-3.845717,-4.42983,-2.068069\H,0,-4.571
518,-3.530869,2.074635\H,0,-4.806236,-5.064892,0.134562\C,0,2.920649,1.50323,1.71
6457\C,0,1.180452,2.55494,0.410735\C,0,1.170332,3.633846,1.294525\C,0,2.037904,3.
65142,2.386507\C,0,2.914504,2.584445,2.595789\H,0,0.487683,2.513567,-0.42489\H,0,
0.473772,4.451824,1.138054\H,0,2.025087,4.488137,3.078857\H,0,3.586209,2.590255,3.
449445\H,0,3.586053,0.664976,1.897338\C,0,4.644771,1.004306,-1.109066\C,0,3.81066
2,-0.828639,-2.444195\C,0,5.008332,-0.863127,-3.15368\C,0,5.83857,0.980516,-1.833
979\C,0,6.026543,0.04512,-2.851002\H,0,4.511817,1.731781,-0.316415\H,0,3.008099,-
1.51768,-2.695224\H,0,5.14345,-1.588376,-3.950716\H,0,6.622973,1.694405,-1.599434
\H,0,6.956563,0.027845,-3.411336\C,0,-3.139821,2.212098,-1.720174\C,0,-4.773053,0.
907653,-0.505119\C,0,-5.754462,1.846121,-0.824082\C,0,-4.119193,3.155759,-2.02941
5\C,0,-5.429011,2.972906,-1.582337\H,0,-2.120728,2.333936,-2.078949\H,0,-5.030507,
0.034466,0.085416\H,0,-6.774184,1.698138,-0.48036\H,0,-3.863166,4.025724,-2.62709
\H,0,-6.195399,3.701597,-1.829609\Pd,0,-0.046678,-0.05458,-1.580309\\Version=ES64
L-G16RevA.03\State=1-A\HF=-2275.4640174\RMSD=3.475e-09\Dipole=0.4138489,0.154263,
1.1815798\Quadrupole=6.3674621,-0.2352116,-6.1322504,-3.3168707,1.154053,0.744926
\PG=C01 [X(C36H28O1P2Pd1)]\

1b

\0,1\C,0,-5.191152,-0.027642,-0.009244\C,0,-5.075654,1.3307,-0.013937\C,0,-3.8736
35,2.101283,-0.081261\C,0,-4.140104,-0.994664,-0.079463\C,0,-2.573687,1.697533,-0.
105082\C,0,-2.78653,-0.843882,-0.110774\C,0,-1.982461,0.370102,-0.059044\H,0,-6.1
97082,-0.436191,0.036408\H,0,-5.997834,1.904241,0.028367\H,0,-4.023877,3.177913,-
0.108121\H,0,-4.482394,-2.026446,-0.112998\H,0,-1.815796,2.474581,-0.145102\H,0,-
2.217164,-1.762427,-0.185917\N,0,-0.667925,0.394888,0.006982\S,0,0.20866,-1.03610
6,0.114095\O,0,0.008474,-1.638967,1.442934\O,0,0.004103,-1.867795,-1.087207\C,0,3.
817116,0.199083,-1.224897\C,0,2.52269,-0.313072,-1.180765\C,0,1.862455,-0.372202,
0.043863\C,0,2.476666,0.056608,1.219076\C,0,3.76991,0.56641,1.15627\C,0,4.456101,
0.65066,-0.063226\H,0,4.342884,0.243196,-2.174514\H,0,2.028419,-0.67894,-2.073237
\H,0,1.949339,-0.027205,2.162379\H,0,4.259479,0.897728,2.067771\C,0,5.845814,1.23

2815,-0.123987\H,0,6.408339,1.019963,0.789425\H,0,5.808009,2.323127,-0.236952\H,0,
6.408826,0.835654,-0.972907\\Version=ES64L-G16RevA.03\State=1-A\HF=-1145.0538221\
RMSD=4.186e-09\Dipole=-1.5534272,2.1054999,-0.2729102\Quadrupole=30.6370046,-14.3
45885,-16.2911196,0.3221568,-0.1795551,1.2555919\PG=C01 [X(C14H13N1O2S1)]\

int-1

\0,1\O,0,-3.085203,-0.710279,-0.805896\C,0,-2.97857,-1.99397,-1.309214\C,0,-3.514
787,-0.48072,0.48611\C,0,-1.76156,-2.689094,-1.190024\C,0,-4.064909,-2.518759,-2.
009722\C,0,-3.970005,-3.782791,-2.589361\C,0,-1.696943,-3.961157,-1.78528\C,0,-2.
784219,-4.506959,-2.469172\H,0,-0.776296,-4.52969,-1.721212\H,0,-2.696714,-5.4928
59,-2.915835\H,0,-4.814572,-4.193304,-3.134723\H,0,-4.965662,-1.919939,-2.097566\
C,0,-3.033934,0.709303,1.059687\C,0,-4.390593,-1.324387,1.166124\C,0,-4.811227,-0.
969499,2.449544\C,0,-3.479935,1.04387,2.344506\C,0,-4.36428,0.213293,3.037281\H,0,
-3.124084,1.956381,2.810501\H,0,-4.69651,0.489471,4.033316\H,0,-4.737208,-2.23966
6,0.699554\H,0,-5.493768,-1.622719,2.985501\P,0,-0.293061,-1.917421,-0.326024\P,0,
-1.740963,1.630177,0.113349\C,0,-0.55335,-2.426162,1.434183\C,0,1.044634,-3.05178
9,-0.915263\C,0,-2.694668,2.376907,-1.284258\C,0,-1.403706,3.06517,1.23353\C,0,-4.
079007,2.600277,-1.225461\C,0,-1.99852,2.729653,-2.450628\C,0,-2.671835,3.308011,
-3.528004\C,0,-4.75045,3.174352,-2.306268\C,0,-4.047969,3.531399,-3.45896\H,0,-4.
6359,2.318395,-0.336755\H,0,-0.930066,2.545767,-2.511481\H,0,-2.119815,3.576165,-
4.424502\H,0,-5.822805,3.339882,-2.247737\H,0,-4.571929,3.976033,-4.300465\C,0,-1.
290756,-3.557873,1.814893\C,0,0.030676,-1.630371,2.432837\C,0,-0.113841,-1.96396,
3.780811\C,0,-0.847827,-3.09304,4.148605\C,0,-1.435847,-3.888482,3.163242\H,0,0.6
0063,-0.750973,2.14516\H,0,0.340388,-1.336229,4.542425\H,0,-0.96545,-3.349402,5.1
97721\H,0,-2.011108,-4.766668,3.443248\H,0,-1.756659,-4.181709,1.058459\C,0,1.590
531,-4.084865,-0.141881\C,0,1.524112,-2.857684,-2.221924\C,0,2.514931,-3.686143,-
2.745336\C,0,2.595698,-4.903719,-0.663634\C,0,3.057203,-4.709645,-1.964448\H,0,1.
233252,-4.255014,0.868258\H,0,1.119319,-2.051073,-2.827736\H,0,2.873555,-3.52435,
-3.757932\H,0,3.016236,-5.694594,-0.048828\H,0,3.840506,-5.345452,-2.366432\C,0,-
0.299525,2.979206,2.096346\C,0,-2.200417,4.220002,1.268356\C,0,-1.90327,5.259425,
2.152096\C,0,-0.009857,4.013728,2.986735\C,0,-0.810932,5.15681,3.015637\H,0,0.346
329,2.107235,2.049136\H,0,-3.050131,4.313719,0.599753\H,0,-2.525249,6.150238,2.16
3111\H,0,0.850405,3.932053,3.644982\H,0,-0.580236,5.967511,3.701098\Pd,0,0.252361,
0.458734,-0.532826\C,0,3.646505,0.461722,-0.576784\C,0,3.284463,0.314782,-2.05450
7\C,0,2.265936,0.833571,-1.113087\H,0,3.22128,-0.712821,-2.400145\H,0,3.752645,1.
011703,-2.745545\C,0,1.518251,1.998919,-0.987642\C,0,1.493368,3.322694,-1.105477\
H,0,2.383617,3.864556,-1.421944\H,0,0.610273,3.913432,-0.882167\C,0,3.857175,-0.8
06103,0.220081\C,0,4.601529,1.536917,-0.1523\O,0,4.634625,2.581646,-1.01204\O,0,3.
387989,-0.687724,1.480688\O,0,4.403953,-1.799235,-0.210562\O,0,5.298255,1.478555,
0.843043\C,0,5.555447,3.640145,-0.667986\C,0,5.58003,4.623945,-1.822796\H,0,6.541
094,3.204195,-0.480184\H,0,5.221396,4.109513,0.263247\H,0,5.902086,4.133536,-2.74
6072\H,0,6.280526,5.435799,-1.602131\H,0,4.591928,5.061445,-1.992516\C,0,3.737621,
-1.75518,2.393375\C,0,5.115364,-1.521514,2.995493\H,0,3.691492,-2.706295,1.858259
\H,0,2.955382,-1.732637,3.155153\H,0,5.884761,-1.563534,2.221141\H,0,5.331151,-2.

294286,3.741703\H,0,5.166271,-0.542018,3.47812\\Version=ES64L-G16RevA.03\State=1-A\HF=-3004.2890348\RMSD=4.745e-09\Dipole=-2.9736872,-0.7869548,0.5584789\Quadrupole=-13.1042405,11.0617575,2.042483,7.74204,-2.278171,-1.3253574\PG=C01 [X(C47H42O5P2Pd1)]\

TS-1

\0,1\O,0,-1.467646,-0.046498,1.877647\C,0,-2.577824,-0.798209,1.558682\C,0,-1.575446,1.290124,2.224785\C,0,-2.318586,-1.897536,0.722868\C,0,-3.859011,-0.523719,2.024996\C,0,-4.906367,-1.370952,1.659383\C,0,-3.382423,-2.737195,0.379725\C,0,-4.672287,-2.478939,0.846203\H,0,-3.199479,-3.587492,-0.267986\H,0,-5.489891,-3.136079,0.568115\H,0,-5.909852,-1.157327,2.014493\H,0,-4.034803,0.344742,2.648341\C,0,-1.328259,2.257853,1.240042\C,0,-1.819357,1.631481,3.551926\C,0,-1.851964,2.976939,3.917246\C,0,-1.377577,3.602212,1.627942\C,0,-1.638343,3.959832,2.950663\H,0,-1.199904,4.375365,0.891045\H,0,-1.665758,5.009474,3.225712\H,0,-1.966406,0.839739,4.278733\H,0,-2.040234,3.252309,4.950053\P,0,-0.634229,-2.029205,0.020384\P,0,-0.945364,1.6895,-0.47111\C,0,0.414605,-2.437599,1.466558\C,0,-0.722213,-3.573336,-0.96582\C,0,-2.640494,1.196702,-1.024808\C,0,-0.698462,3.234124,-1.448335\C,0,-3.792361,1.8085,-0.512302\C,0,-2.781221,0.203086,-2.002455\C,0,-4.044553,-0.187914,-2.444013\C,0,-5.055716,1.41783,-0.952277\C,0,-5.185592,0.414617,-1.914008\H,0,-3.701072,2.584117,0.240304\H,0,-1.889038,-0.269056,-2.402839\H,0,-4.136227,-0.968773,-3.193121\H,0,-5.940255,1.893103,-0.538508\H,0,-6.171142,0.10399,-2.247708\C,0,-0.094305,-3.086022,2.601321\C,0,1.756494,-2.034058,1.449506\C,0,2.585287,-2.277721,2.544393\C,0,2.074922,-2.933442,3.665288\C,0,0.736498,-3.33737,3.692486\H,0,2.159739,-1.499935,0.597496\H,0,3.606656,-1.915983,2.504064\H,0,2.713562,-3.120835,4.523786\H,0,0.336895,-3.840069,4.568696\H,0,-1.140408,-3.375416,2.635825\C,0,-0.608503,-4.848978,-0.397481\C,0,-0.89899,-3.454894,-2.352109\C,0,-0.977049,-4.592004,-3.155826\C,0,-0.678763,-5.9856,-1.203274\C,0,-0.866348,-5.859712,-2.581304\H,0,-0.453179,-4.950123,0.671495\H,0,-0.957781,-2.465104,-2.79629\H,0,-1.112239,-4.488502,-4.228358\H,0,-0.583669,-6.970238,-0.755229\H,0,-0.917445,-6.746325,-3.206094\C,0,0.291576,4.171573,-1.103936\C,0,-1.450398,3.446591,-2.61492\C,0,-1.222153,4.567115,-3.413714\C,0,0.503434,5.297127,-1.899642\C,0,-0.248398,5.499606,-3.057615\H,0,0.898726,3.997307,-0.224987\H,0,-2.219201,2.740146,-2.903328\H,0,-1.814283,4.711434,-4.312671\H,0,1.267939,6.013778,-1.613718\H,0,-0.075782,6.374615,-3.677164\Pd,0,0.40791,-0.176317,-1.016512\C,0,3.317668,1.253027,-0.201612\C,0,2.913939,1.842625,-1.544143\C,0,2.078688,0.67043,-1.868091\H,0,3.751588,1.977742,-2.228059\H,0,2.345187,2.761889,-1.431937\C,0,2.12481,-0.547797,-2.33511\C,0,2.431969,-1.848497,-2.434582\H,0,1.759977,-2.572358,-2.8868\H,0,3.39063,-2.225736,-2.07404\C,0,4.361445,0.246434,-0.164569\C,0,2.509893,1.591423,0.940896\O,0,2.858464,0.980567,2.098788\O,0,5.006051,0.163812,-1.382856\O,0,4.682174,-0.490893,0.760873\O,0,1.552301,2.379935,0.902293\C,0,1.955907,1.152745,3.205239\C,0,2.136761,2.489174,3.908631\H,0,2.190841,0.316729,3.86818\H,0,0.931248,1.038937,2.849851\H,0,3.17455,2.620565,4.230135\H,0,1.491459,2.532115,4.793101\H,0,1.862179,3.305835,3.23948\C,0,6.02275,-0.842109,-1.484223\C,0,7.352329,-0.350977,-0.930052\H,0,6.092885,-1.060274,-2.553856\H,0,5.69536,-1.739241,-0.950414\H,0,7.660036,0.572396,-1.429955\H,0,8.13

1233,-1.105337,-1.086032\H,0,7.26055,-0.160868,0.141283\\Version=ES64L-G16RevA.03
\State=1-A\HF=-3004.2492851\RMSD=8.826e-09\Dipole=-3.7385249,-1.1681703,-0.373177
\Quadrupole=-12.4554045,8.5728986,3.8825059,-2.1100386,-8.0539929,7.0210509\PG=C0
1 [X(C47H42O5P2Pd1)]\

int-2a

\0,1\O,0,0.870045,2.023631,1.640291\C,0,0.900192,2.778914,0.483969\C,0,2.049196,1.
545446,2.174728\C,0,-0.204049,2.602405,-0.35837\C,0,1.923352,3.67203,0.187235\C,0,
1.838879,4.418226,-0.988833\C,0,-0.281737,3.381844,-1.516612\C,0,0.736717,4.28253
7,-1.83289\H,0,-1.127913,3.2629,-2.183284\H,0,0.674434,4.868163,-2.744378\H,0,2.6
40892,5.103274,-1.243728\H,0,2.773392,3.763693,0.853765\C,0,2.745553,0.495219,1.5
54227\C,0,2.4674,2.095692,3.383424\C,0,3.61843,1.611637,4.001981\C,0,3.904112,0.0
29064,2.197782\C,0,4.340536,0.58093,3.401988\H,0,4.463055,-0.785837,1.755139\H,0,
5.238866,0.194888,3.872991\H,0,1.874027,2.891902,3.819623\H,0,3.945693,2.037557,4.
945193\P,0,-1.335874,1.220611,0.065587\P,0,2.156395,-0.272478,-0.027771\C,0,-2.30
3368,1.862283,1.487828\C,0,-2.510813,1.287614,-1.343951\C,0,2.886215,0.810294,-1.
311781\C,0,3.181749,-1.795049,-0.036648\C,0,4.115632,1.461091,-1.137232\C,0,2.181
398,0.978415,-2.509211\C,0,2.701412,1.783334,-3.521878\C,0,4.635359,2.261415,-2.1
52617\C,0,3.928317,2.42356,-3.346247\H,0,4.657509,1.349656,-0.203432\H,0,1.227023,
0.479205,-2.635821\H,0,2.143828,1.915558,-4.44398\H,0,5.588165,2.76288,-2.010742\
H,0,4.330881,3.052868,-4.134412\C,0,-2.058678,3.12624,2.041442\C,0,-3.316069,1.05
4238,2.028622\C,0,-4.062377,1.513084,3.114232\C,0,-3.806943,2.766874,3.672933\C,0,
-2.805212,3.573199,3.131642\H,0,-3.517567,0.079959,1.597924\H,0,-4.846952,0.88303,
3.523264\H,0,-4.388061,3.116008,4.521632\H,0,-2.605456,4.554106,3.553279\H,0,-1.2
92281,3.766905,1.620839\C,0,-3.705343,2.015926,-1.300525\C,0,-2.147668,0.635176,-
2.532438\C,0,-2.951598,0.735121,-3.666326\C,0,-4.517642,2.099669,-2.432079\C,0,-4.
138971,1.468203,-3.618233\H,0,-3.998924,2.513456,-0.382887\H,0,-1.241369,0.036188,
-2.558557\H,0,-2.659894,0.224349,-4.579063\H,0,-5.445353,2.66234,-2.387141\H,0,-4.
771316,1.539042,-4.498276\C,0,2.725705,-2.881688,0.727002\C,0,4.387306,-1.896959,
-0.736767\C,0,5.139913,-3.072633,-0.66756\C,0,3.485602,-4.045639,0.800266\C,0,4.6
93095,-4.145157,0.102688\H,0,1.765718,-2.83453,1.234587\H,0,4.737167,-1.068264,-1.
341617\H,0,6.071137,-3.147538,-1.22141\H,0,3.123053,-4.882515,1.389141\H,0,5.2773
54,-5.059213,0.153253\Pd,0,-0.114788,-0.848694,-0.246822\C,0,-1.65107,-2.405443,0.
021551\C,0,-0.8368,-3.405827,-0.826475\C,0,0.28692,-2.545386,-1.305277\H,0,-1.423
9,-3.833098,-1.64103\H,0,-0.468011,-4.210741,-0.183234\C,0,1.120385,-2.586002,-2.
289303\C,0,2.030973,-2.588781,-3.243268\H,0,3.013374,-3.025466,-3.079801\H,0,1.83
6485,-2.164349,-4.225993\C,0,-2.973642,-1.983236,-0.465939\C,0,-1.376357,-2.51907
6,1.474992\O,0,-2.169678,-1.757429,2.263188\O,0,-3.159156,-2.343387,-1.77146\O,0,
-3.841529,-1.375282,0.146035\O,0,-0.47667,-3.214544,1.944946\C,0,-1.84903,-1.7298
99,3.667037\C,0,-2.473827,-2.906051,4.400519\H,0,-2.25693,-0.777374,4.013067\H,0,
-0.762442,-1.725142,3.7847\H,0,-3.554712,-2.931436,4.232879\H,0,-2.292429,-2.8180
59,5.477247\H,0,-2.038569,-3.844276,4.049941\C,0,-4.432365,-1.990922,-2.344753\C,
0,-5.494917,-3.021387,-1.995717\H,0,-4.246603,-1.954928,-3.420505\H,0,-4.717884,-
0.996561,-1.998185\H,0,-5.181347,-4.022668,-2.306222\H,0,-6.434623,-2.780435,-2.5

04464\H,0,-5.6764,-3.02475,-0.918683\\Version=ES64L-G16RevA.03\State=1-A\HF=-3004.
2957872\RMSD=6.316e-09\Dipole=2.0389451,3.2335997,-0.3446288\Quadrupole=9.5362846,
-14.8165882,5.2803035,2.2551776,7.8643551,-1.0645385\PG=C01 [X(C47H42O5P2Pd1)]\

int-2b

\0,1\O,0,1.433408,-2.533992,-1.061993\C,0,2.796805,-2.333634,-1.194614\C,0,1.0151
94,-3.02499,0.17672\C,0,3.396417,-1.204472,-0.608492\C,0,3.541569,-3.257559,-1.92
1367\C,0,4.923661,-3.097429,-2.026657\C,0,4.790413,-1.080328,-0.702312\C,0,5.5501
59,-2.021809,-1.396536\H,0,5.279403,-0.226623,-0.247864\H,0,6.627318,-1.904918,-1.
456601\H,0,5.508076,-3.819803,-2.587576\H,0,3.028494,-4.096886,-2.378283\C,0,-0.1
38568,-2.474473,0.752202\C,0,1.732241,-4.049189,0.793943\C,0,1.321432,-4.526914,2.
035076\C,0,-0.516385,-2.964652,2.012695\C,0,0.201037,-3.973716,2.650568\H,0,-1.37
7259,-2.536671,2.507457\H,0,-0.117173,-4.320516,3.628191\H,0,2.612267,-4.453448,0.
307905\H,0,1.882888,-5.320648,2.518081\P,0,2.324979,0.11753,0.076035\P,0,-1.05911
2,-1.080327,-0.049543\C,0,2.010116,-0.263645,1.829395\C,0,3.420325,1.590175,0.124
845\C,0,-1.946587,-1.845352,-1.465787\C,0,-2.410618,-0.820126,1.152692\C,0,-1.494
11,-2.985103,-2.139335\C,0,-3.108709,-1.194022,-1.911303\C,0,-3.800327,-1.679866,
-3.017983\C,0,-2.191837,-3.46415,-3.250218\C,0,-3.342561,-2.813493,-3.694189\H,0,
-0.601461,-3.500035,-1.809505\H,0,-3.486005,-0.323649,-1.384232\H,0,-4.702664,-1.
172163,-3.345052\H,0,-1.834785,-4.351883,-3.764248\H,0,-3.883451,-3.19047,-4.5571
01\C,0,2.717119,-1.248497,2.526298\C,0,0.998422,0.467318,2.472837\C,0,0.69378,0.1
98124,3.80568\C,0,1.3875,-0.798088,4.495661\C,0,2.398565,-1.517733,3.856744\H,0,0.
448235,1.241288,1.942503\H,0,-0.098506,0.757586,4.291895\H,0,1.136772,-1.017348,5.
529152\H,0,2.933252,-2.29786,4.389678\H,0,3.492241,-1.818476,2.025858\C,0,3.52650
6,2.37299,1.280947\C,0,4.077619,2.009505,-1.044166\C,0,4.8179,3.188492,-1.055269\
C,0,4.260518,3.560455,1.263039\C,0,4.903215,3.973666,0.097617\H,0,3.025919,2.0679
65,2.191775\H,0,4.019566,1.406177,-1.943449\H,0,5.32257,3.497922,-1.965394\H,0,4.
323687,4.161949,2.16436\H,0,5.4693,4.899755,0.084972\C,0,-2.399081,0.272162,2.020
558\C,0,-3.417842,-1.798149,1.271839\C,0,-4.378445,-1.69175,2.27148\C,0,-3.37173,
0.373783,3.019448\C,0,-4.347709,-0.608338,3.15767\H,0,-1.703599,1.092961,1.886821
\H,0,-3.439122,-2.640978,0.589253\H,0,-5.152327,-2.448833,2.3582\H,0,-3.381869,1.
256308,3.648046\H,0,-5.10389,-0.520667,3.932382\Pd,0,0.393306,0.471868,-1.106856\
C,0,-2.481538,2.507441,-0.545053\C,0,-2.426661,2.281215,-2.045944\C,0,-1.159159,1.
6605,-2.462374\H,0,-2.481793,3.220227,-2.621247\H,0,-3.27953,1.674281,-2.347848\C,
0,-0.019583,1.580395,-2.960062\C,0,1.361683,1.429328,-2.79009\H,0,1.92151,0.74299
4,-3.423255\H,0,1.907287,2.315331,-2.474173\C,0,-1.363207,3.069847,0.136015\C,0,-
3.691789,2.250188,0.166843\O,0,-4.589757,1.47452,-0.587378\O,0,-0.485894,3.707435,
-0.742559\O,0,-1.069736,3.013368,1.340221\O,0,-4.012293,2.620289,1.291539\C,0,-5.
834463,1.183503,0.05112\C,0,-6.455902,-0.001398,-0.667858\H,0,-6.487466,2.064893,
0.002478\H,0,-5.659527,0.96585,1.106746\H,0,-6.58919,0.210658,-1.733956\H,0,-7.43
74,-0.232544,-0.240356\H,0,-5.821141,-0.886759,-0.569043\C,0,0.764796,4.12146,-0.
189443\C,0,0.671419,5.482816,0.483107\H,0,1.460649,4.149609,-1.03468\H,0,1.112528,
3.374241,0.526014\H,0,0.301301,6.237339,-0.217926\H,0,1.659889,5.795297,0.839811\
H,0,-0.010515,5.424708,1.333627\\Version=ES64L-G16RevA.03\State=1-A\HF=-3004.2776

176\RMSD=4.811e-09\Dipole=5.2778149,-4.2466143,-0.7552586\Quadrupole=10.6342213,-10.3376646,-0.2965568,16.4247022,-0.627796,-7.5685467\PG=C01 [X(C47H42O5P2Pd1)]\

TS-1’

\0,1\O,0,1.385824,-1.639844,1.551225\C,0,0.33106,-2.251028,2.211093\C,0,2.035963,-2.282862,0.517754\C,0,-0.974081,-2.180753,1.688571\C,0,0.600526,-2.843808,3.441275\C,0,-0.438788,-3.403884,4.183322\C,0,-1.998977,-2.758102,2.452885\C,0,-1.739469,-3.362885,3.683268\H,0,-3.016521,-2.720284,2.082842\H,0,-2.557346,-3.796472,4.250336\H,0,-0.231126,-3.864668,5.143969\H,0,1.623963,-2.846628,3.801112\C,0,2.602273,-1.439348,-0.452822\C,0,2.152008,-3.666554,0.442294\C,0,2.862898,-4.229111,-0.618621\C,0,3.329033,-2.027398,-1.493328\C,0,3.460441,-3.413992,-1.57909\H,0,3.772312,-1.39383,-2.253604\H,0,4.015604,-3.854987,-2.400873\H,0,1.678562,-4.289007,1.19278\H,0,2.944841,-5.309118,-0.690571\P,0,-1.281086,-1.2975,0.088913\P,0,2.154718,0.337962,-0.373175\C,0,-0.754041,-2.58804,-1.11827\C,0,-3.116618,-1.398724,-0.065222\C,0,3.231177,0.955159,0.988646\C,0,2.994843,1.016363,-1.863439\C,0,4.293739,0.20818,1.513765\C,0,2.96717,2.225522,1.521527\C,0,3.757297,2.743683,2.546006\C,0,5.07695,0.722306,2.548575\C,0,4.813517,1.991582,3.064752\H,0,4.504945,-0.777971,1.113509\H,0,2.116367,2.792796,1.162535\H,0,3.539903,3.729283,2.947451\H,0,5.894278,0.130167,2.950118\H,0,5.423488,2.390296,3.869867\C,0,-0.991895,-3.952714,-0.901187\C,0,-0.131321,-2.186114,-2.304414\C,0,0.251051,-3.129228,-3.258898\C,0,0.010583,-4.484541,-3.035417\C,0,-0.612817,-4.895081,-1.854379\H,0,0.065269,-1.129343,-2.457819\H,0,0.74744,-2.80524,-4.168752\H,0,0.313321,-5.220331,-3.77468\H,0,-0.799214,-5.950145,-1.675414\H,0,-1.472164,-4.274172,0.017681\C,0,-3.755425,-2.1145,-1.089474\C,0,-3.898229,-0.640691,0.82265\C,0,-5.286719,-0.625216,0.703506\C,0,-5.145309,-2.084122,-1.212771\C,0,-5.916156,-1.346714,-0.312938\H,0,-3.171091,-2.698478,-1.7905\H,0,-3.420592,-0.03454,1.584791\H,0,-5.870837,-0.02267,1.391574\H,0,-5.62447,-2.64274,-2.011974\H,0,-6.997735,-1.325107,-0.409611\C,0,2.207829,1.224836,-3.006249\C,0,4.359314,1.335136,-1.910896\C,0,4.924421,1.842329,-3.081035\C,0,2.774659,1.723628,-4.178968\C,0,4.135099,2.034289,-4.217247\H,0,1.145304,0.999769,-2.959979\H,0,4.976535,1.186174,-1.030807\H,0,5.982092,2.088197,-3.106521\H,0,2.154286,1.879508,-5.056724\H,0,4.57759,2.431246,-5.126031\Pd,0,-0.143389,0.722523,-0.202254\C,0,-1.573515,2.590047,0.231253\C,0,-0.187674,3.045337,-0.806533\C,0,-1.177521,3.94907,-0.240174\H,0,0.820474,3.150583,-0.42748\H,0,-0.289031,2.790597,-1.857233\C,0,-1.582799,5.172433,-0.141059\C,0,-1.998999,6.408516,-0.03241\H,0,-1.632008,7.063881,0.754046\H,0,-2.731611,6.82375,-0.720489\C,0,-2.878396,2.182028,-0.403488\C,0,-1.407084,2.268354,1.690074\O,0,-0.245345,2.768868,2.172804\O,0,-2.717901,1.747349,-1.671747\O,0,-3.950492,2.306063,0.1438\O,0,-2.208644,1.662957,2.37508\C,0,0.076495,2.405967,3.539077\C,0,0.459318,0.940613,3.67752\H,0,-0.778404,2.654406,4.173386\H,0,0.914612,3.060251,3.786325\H,0,-0.395351,0.295735,3.473326\H,0,0.798315,0.753003,4.70224\H,0,1.267245,0.680832,2.992316\C,0,-3.933933,1.389605,-2.373834\C,0,-4.604559,2.617754,-2.965487\H,0,-4.593289,0.864294,-1.68201\H,0,-3.598463,0.695193,-3.146364\H,0,-4.935116,3.289568,-2.170211\H,0,-5.480433,2.318329,-3.550577\H,0,-3.918101,3.157813,-3.624614\\Version=ES64L-G16RevA.03\State=1-A\HF=-3004.2396963\RMSD=2.641e-09\Dipole=1.8746702,-1.2196125,-1.008194\Quadrupole=-8.720568

7,1.0609157,7.659653,7.2838934,9.4840256,-3.1112505\PG=C01 [X(C47H42O5P2Pd1)]\

int-2a’

\0,1\O,0,-1.280782,1.25078,1.983367\C,0,-0.16566,1.856337,2.551596\C,0,-2.098271,
2.040017,1.186218\C,0,0.996374,2.052845,1.785411\C,0,-0.235929,2.219813,3.892283\
C,0,0.859587,2.835896,4.497222\C,0,2.07348,2.69616,2.411703\C,0,2.007182,3.089453,
3.748236\H,0,2.985113,2.864942,1.852497\H,0,2.861521,3.578163,4.205349\H,0,0.8103
65,3.119823,5.543646\H,0,-1.151065,2.019267,4.439366\C,0,-2.609566,1.448379,0.021
667\C,0,-2.411513,3.348972,1.544096\C,0,-3.231529,4.105572,0.710411\C,0,-3.410481,
2.239907,-0.811085\C,0,-3.72093,3.55616,-0.473569\H,0,-3.772773,1.832711,-1.74623
\H,0,-4.336417,4.151022,-1.140412\H,0,-1.99749,3.768609,2.453473\H,0,-3.473347,5.
128531,0.981712\P,0,1.125662,1.325223,0.093494\P,0,-2.084522,-0.27245,-0.37236\C,
0,0.30266,2.534063,-1.015405\C,0,2.896848,1.580571,-0.331682\C,0,-3.19989,-1.3167
37,0.652495\C,0,-2.682345,-0.582802,-2.074402\C,0,-3.46704,-0.992286,1.990775\C,0,
-3.691306,-2.525804,0.137249\C,0,-4.439278,-3.386232,0.940244\C,0,-4.217241,-1.85
4997,2.788881\C,0,-4.705368,-3.054477,2.268326\H,0,-3.074616,-0.079448,2.418628\H,
0,-3.49025,-2.798251,-0.892029\H,0,-4.810407,-4.318194,0.524717\H,0,-4.416888,-1.
588519,3.822555\H,0,-5.286035,-3.725615,2.893707\C,0,0.075293,3.869444,-0.668885\
C,0,-0.074126,2.083011,-2.288277\C,0,-0.67584,2.949159,-3.198133\C,0,-0.91217,4.2
77863,-2.840572\C,0,-0.536852,4.734239,-1.576312\H,0,0.102008,1.047853,-2.557356\
H,0,-0.96986,2.582296,-4.176909\H,0,-1.39158,4.953952,-3.542168\H,0,-0.725052,5.7
6532,-1.29252\H,0,0.36467,4.227626,0.313383\C,0,3.284674,2.365116,-1.42806\C,0,3.
880373,0.941372,0.439063\C,0,5.228235,1.096314,0.118162\C,0,4.635334,2.508664,-1.
746249\C,0,5.610687,1.879058,-0.971974\H,0,2.539392,2.867352,-2.032842\H,0,3.5924
08,0.304182,1.269589\H,0,5.973799,0.578825,0.712801\H,0,4.922696,3.118218,-2.5981
07\H,0,6.661639,1.991559,-1.222009\C,0,-1.740344,-0.938686,-3.049205\C,0,-4.04039
3,-0.522816,-2.425569\C,0,-4.440371,-0.77411,-3.736683\C,0,-2.142347,-1.192011,-4.
361316\C,0,-3.490598,-1.101763,-4.707916\H,0,-0.697545,-1.041241,-2.762288\H,0,-4.
784267,-0.29645,-1.668389\H,0,-5.492705,-0.722758,-3.99926\H,0,-1.403678,-1.47077
9,-5.106552\H,0,-3.804713,-1.300785,-5.728105\Pd,0,0.136723,-0.851408,-0.072599\C,
0,1.827933,-2.14447,0.356287\C,0,-0.405914,-2.873801,-0.155582\C,0,1.021772,-3.30
1203,-0.20711\H,0,-0.906642,-3.087914,0.788965\H,0,-1.011081,-3.165032,-1.015642\
C,0,1.536296,-4.317589,-0.857925\C,0,2.063234,-5.284076,-1.565738\H,0,2.298756,-6.
254747,-1.131416\H,0,2.297704,-5.158207,-2.622934\C,0,3.095609,-1.879668,-0.37770
2\C,0,1.876156,-1.953201,1.821362\O,0,0.998196,-2.762008,2.475654\O,0,2.849103,-1.
453121,-1.650945\O,0,4.220674,-2.040445,0.05278\O,0,2.537809,-1.112679,2.419132\C,
0,0.691783,-2.396011,3.831067\C,0,-0.609792,-3.085132,4.197439\H,0,0.608756,-1.30
6507,3.895608\H,0,1.516355,-2.703413,4.483359\H,0,-1.42272,-2.744855,3.549331\H,0,
-0.875417,-2.858992,5.235615\H,0,-0.51842,-4.17025,4.093849\C,0,4.00159,-1.297318,
-2.499438\C,0,4.366336,-2.616802,-3.161886\H,0,4.829262,-0.909652,-1.903633\H,0,3.
705918,-0.542281,-3.231975\H,0,4.632757,-3.356878,-2.403952\H,0,5.220782,-2.48080
8,-3.833856\H,0,3.524626,-3.004075,-3.744052\\Version=ES64L-G16RevA.03\State=1-A\
HF=-3004.2964132\RMSD=2.608e-09\Dipole=-3.6373308,3.6229062,-1.2151649\Quadrupole
=-9.4520532,-8.0535406,17.5055938,11.7972659,-4.6061035,5.9840071\PG=C01 [X(C47H4

2O5P2Pd1)]\

TS-1”

\0,1\O,0,1.773058,-0.807123,-2.085264\C,0,2.653389,0.22984,-2.35215\C,0,2.208971,
-1.916192,-1.386247\C,0,2.950451,1.18993,-1.367157\C,0,3.163181,0.326247,-3.64365
2\C,0,4.002822,1.387984,-3.980488\C,0,3.800077,2.245263,-1.730211\C,0,4.324711,2.
345368,-3.019358\H,0,4.042483,3.003192,-0.993828\H,0,4.978218,3.174896,-3.270884\
H,0,4.399329,1.464327,-4.988062\H,0,2.885957,-0.432391,-4.36777\C,0,1.242887,-2.5
62969,-0.596566\C,0,3.517463,-2.383287,-1.472228\C,0,3.875649,-3.53271,-0.768725\
C,0,1.624293,-3.728267,0.079785\C,0,2.929012,-4.213289,-0.003572\H,0,0.898495,-4.
240814,0.700874\H,0,3.20849,-5.109699,0.540744\H,0,4.243376,-1.843004,-2.068915\H,
0,4.898612,-3.891946,-0.82064\P,0,2.185801,1.062475,0.312541\P,0,-0.346516,-1.688
703,-0.310472\C,0,3.343239,-0.10341,1.141558\C,0,2.625729,2.691987,1.046534\C,0,-
1.377442,-2.09042,-1.763543\C,0,-1.106619,-2.685329,1.031326\C,0,-0.869135,-2.699
341,-2.917364\C,0,-2.720343,-1.676606,-1.737302\C,0,-3.539245,-1.886064,-2.844962
\C,0,-1.69037,-2.895607,-4.02833\C,0,-3.025462,-2.489106,-3.995936\H,0,0.165598,-
3.023435,-2.945418\H,0,-3.117578,-1.18494,-0.857767\H,0,-4.575463,-1.562665,-2.81
2501\H,0,-1.287163,-3.370456,-4.918186\H,0,-3.662587,-2.642523,-4.862015\C,0,4.72
2121,-0.10865,0.884124\C,0,2.816651,-1.024308,2.053843\C,0,3.651629,-1.932818,2.7
04216\C,0,5.022445,-1.927061,2.448333\C,0,5.557006,-1.012994,1.537014\H,0,1.74667
4,-1.03881,2.231025\H,0,3.227892,-2.651869,3.39862\H,0,5.672646,-2.637983,2.94956
1\H,0,6.623336,-1.0104,1.330269\H,0,5.137715,0.589054,0.163847\C,0,3.642347,2.884
866,1.989732\C,0,1.843375,3.791479,0.653622\C,0,2.089772,5.058979,1.174068\C,0,3.
874946,4.154312,2.525161\C,0,3.105814,5.243014,2.116443\H,0,4.251851,2.046839,2.3
08336\H,0,1.038019,3.638177,-0.059497\H,0,1.482014,5.900825,0.855637\H,0,4.661911,
4.289702,3.261439\H,0,3.290104,6.228424,2.533693\C,0,-0.974429,-2.222547,2.348276
\C,0,-1.791978,-3.886091,0.795323\C,0,-2.320708,-4.615841,1.860146\C,0,-1.488844,
-2.960731,3.414247\C,0,-2.161978,-4.159648,3.171506\H,0,-0.498101,-1.263531,2.527
003\H,0,-1.911976,-4.242837,-0.222588\H,0,-2.854372,-5.541887,1.667147\H,0,-1.388
901,-2.584019,4.427351\H,0,-2.574131,-4.729788,3.998754\Pd,0,-0.103828,0.59257,0.
175412\C,0,-2.881117,1.752047,0.822687\C,0,-1.470762,2.299759,1.001893\C,0,-2.037
036,1.343636,-0.333446\H,0,-0.995382,1.993769,1.935185\H,0,-1.321201,3.329815,0.7
06939\C,0,-2.242719,1.498363,-1.617579\C,0,-2.444763,1.622103,-2.90762\H,0,-2.449
946,2.599846,-3.391065\H,0,-2.611048,0.756238,-3.54715\C,0,-3.242236,0.614658,1.7
6501\C,0,-4.039802,2.685496,0.593713\O,0,-3.66915,3.775725,-0.10581\O,0,-4.024437,
-0.279876,1.14175\O,0,-2.865309,0.515649,2.912671\O,0,-5.169459,2.477024,0.987035
\C,0,-4.734131,4.667738,-0.505302\C,0,-5.444941,4.13744,-1.740268\H,0,-4.231069,5.
616587,-0.703361\H,0,-5.426869,4.789078,0.33088\H,0,-4.72869,3.948269,-2.544338\H,
0,-6.183429,4.866555,-2.089484\H,0,-5.962467,3.204141,-1.507984\C,0,-4.522406,-1.
371509,1.95241\C,0,-5.28844,-2.302922,1.035393\H,0,-5.158944,-0.949217,2.736221\H,
0,-3.678111,-1.870476,2.42781\H,0,-6.112037,-1.777815,0.543337\H,0,-5.70237,-3.13
1696,1.61797\H,0,-4.630115,-2.720299,0.269612\\Version=ES64L-G16RevA.03\State=1-A
\HF=-3004.2421819\RMSD=5.129e-09\Dipole=2.3001853,-0.6207599,-0.5928726\Quadrupol
e=0.0429674,10.609178,-10.6521454,5.6533704,4.3688248,-10.1512982\PG=C01 [X(C47H4

2O5P2Pd1)]\

int-2a”

\0,1\O,0,-2.761656,-1.163209,1.40341\C,0,-3.556485,-0.070534,1.701602\C,0,-3.0150
06,-1.827197,0.213135\C,0,-3.284143,1.179138,1.117963\C,0,-4.595756,-0.246697,2.6
11545\C,0,-5.42354,0.828227,2.931975\C,0,-4.144241,2.240306,1.445479\C,0,-5.20527
1,2.068989,2.333974\H,0,-3.969273,3.216086,1.009078\H,0,-5.851295,2.909687,2.5661
11\H,0,-6.235888,0.693057,3.639152\H,0,-4.739022,-1.228582,3.049717\C,0,-1.887348,
-2.271411,-0.488627\C,0,-4.307551,-2.06264,-0.24611\C,0,-4.483176,-2.744591,-1.45
0284\C,0,-2.088738,-2.951935,-1.694921\C,0,-3.377502,-3.183223,-2.17764\H,0,-1.22
9439,-3.29122,-2.261334\H,0,-3.51451,-3.703959,-3.119806\H,0,-5.157373,-1.703583,
0.322949\H,0,-5.487962,-2.92577,-1.81962\P,0,-1.782225,1.444062,0.069317\P,0,-0.2
40748,-1.825361,0.189374\C,0,-2.275193,0.971513,-1.629285\C,0,-1.725104,3.275984,
0.008549\C,0,-0.121302,-2.870656,1.688769\C,0,0.875169,-2.619282,-1.032488\C,0,-0.
83797,-4.066902,1.828365\C,0,0.719472,-2.444122,2.725963\C,0,0.849808,-3.214903,3.
880624\C,0,-0.70592,-4.833754,2.985895\C,0,0.139944,-4.409651,4.012428\H,0,-1.504
538,-4.392762,1.035905\H,0,1.263342,-1.511705,2.619499\H,0,1.502975,-2.878142,4.6
80216\H,0,-1.265539,-5.759101,3.087258\H,0,0.240421,-5.005981,4.914599\C,0,-3.593
502,0.696244,-2.00361\C,0,-1.244793,0.867577,-2.57954\C,0,-1.540006,0.480291,-3.8
84665\C,0,-2.855865,0.184341,-4.249071\C,0,-3.880198,0.295029,-3.308933\H,0,-0.22
0151,1.092435,-2.294337\H,0,-0.738976,0.400809,-4.613368\H,0,-3.081467,-0.132554,
-5.263047\H,0,-4.903716,0.062886,-3.587369\H,0,-4.391074,0.776955,-1.273355\C,0,-
2.07148,4.006218,-1.133189\C,0,-1.28877,3.959024,1.154689\C,0,-1.211877,5.348571,
1.160549\C,0,-1.983135,5.400333,-1.128064\C,0,-1.55667,6.072983,0.016148\H,0,-2.4
06911,3.488841,-2.024961\H,0,-1.000602,3.395108,2.036539\H,0,-0.868674,5.865865,2.
051192\H,0,-2.249124,5.958076,-2.021036\H,0,-1.485396,7.15641,0.016618\C,0,1.3662
43,-1.830181,-2.081942\C,0,1.221003,-3.974269,-0.971273\C,0,2.041298,-4.536074,-1.
950417\C,0,2.172817,-2.397908,-3.067481\C,0,2.515324,-3.750531,-3.002406\H,0,1.14
305,-0.770157,-2.111572\H,0,0.860798,-4.587134,-0.152137\H,0,2.312448,-5.585896,-
1.888731\H,0,2.543734,-1.777844,-3.877854\H,0,3.15344,-4.188438,-3.764356\Pd,0,0.
30668,0.45737,0.49945\C,0,2.715597,1.601968,0.268328\C,0,1.359528,2.253299,0.5656
95\C,0,2.341569,0.159715,0.597533\H,0,1.028043,2.982525,-0.170972\H,0,1.336781,2.
672107,1.572194\C,0,3.081327,-0.897223,0.709096\C,0,3.759057,-2.018783,0.822316\H,
0,4.142204,-2.36348,1.779789\H,0,3.957256,-2.643359,-0.045615\C,0,3.021767,1.5513
83,-1.226463\C,0,3.879633,2.253631,1.010745\O,0,4.718738,1.359867,1.557118\O,0,4.
348509,1.436994,-1.432587\O,0,2.187991,1.52608,-2.112308\O,0,4.017477,3.456362,1.
106108\C,0,5.875746,1.902965,2.218683\C,0,6.670871,0.728518,2.754455\H,0,5.548895,
2.580899,3.013623\H,0,6.448518,2.495225,1.49732\H,0,6.077716,0.15633,3.473471\H,0,
7.577427,1.08296,3.255013\H,0,6.95931,0.057057,1.941075\C,0,4.805092,1.046856,-2.
745213\C,0,5.27585,-0.397192,-2.685964\H,0,5.621864,1.730438,-2.99156\H,0,3.99088,
1.187381,-3.458425\H,0,6.064483,-0.514275,-1.93802\H,0,5.66753,-0.708511,-3.66048
\H,0,4.449256,-1.057593,-2.41384\\Version=ES64L-G16RevA.03\State=1-A\HF=-3004.286
8796\RMSD=5.605e-09\Dipole=-2.6642309,-1.6084197,-0.5822436\Quadrupole=10.7488266,
-11.7816994,1.0328728,-9.9644128,-5.9461656,3.2046418\PG=C01 [X(C47H42O5P2Pd1)]\

int-3

\0,1\C,0,-3.623331,-3.56011,-1.246566\C,0,-4.591086,-2.830316,-1.921008\C,0,-4.58
3226,-1.45083,-2.168759\C,0,-2.44601,-3.090449,-0.656158\C,0,-3.639287,-0.480989,
-1.876634\C,0,-1.934684,-1.80395,-0.560784\C,0,-2.380946,-0.605034,-1.226046\H,0,
-3.794497,-4.630489,-1.161356\H,0,-5.450245,-3.379864,-2.295699\H,0,-5.456297,-1.
073768,-2.696989\H,0,-1.82896,-3.846445,-0.187868\H,0,-3.880202,0.507335,-2.24048
4\H,0,-0.946893,-1.721535,-0.121043\N,0,-1.484911,0.403561,-1.159662\S,0,-1.52873
7,1.767216,-2.141309\O,0,-0.159371,2.31749,-2.111188\O,0,-2.106109,1.45229,-3.457
594\C,0,-3.379687,5.213489,-1.210307\C,0,-2.535841,4.265561,-1.779797\C,0,-2.5980
78,2.944505,-1.328469\C,0,-3.474656,2.563906,-0.31735\C,0,-4.311471,3.530511,0.23
8743\C,0,-4.283454,4.860053,-0.196028\H,0,-3.332297,6.244578,-1.550035\H,0,-1.829
581,4.545229,-2.553965\H,0,-3.502783,1.559011,0.086068\H,0,-4.977201,3.232206,1.0
43085\C,0,-5.210175,5.888354,0.402336\H,0,-4.775296,6.891341,0.362672\H,0,-5.4406
31,5.655168,1.445587\H,0,-6.161658,5.92268,-0.142722\O,0,2.317306,-2.063752,0.947
04\C,0,3.555742,-1.441722,0.9149\C,0,2.066858,-2.988069,-0.049703\C,0,3.582042,-0.
065701,1.172145\C,0,4.71556,-2.16592,0.659963\C,0,5.941763,-1.501601,0.631101\C,0,
4.822727,0.582647,1.114078\C,0,5.992715,-0.124761,0.844941\H,0,4.871098,1.654263,
1.256073\H,0,6.939362,0.402972,0.794226\H,0,6.850464,-2.0594,0.426811\H,0,4.6543,
-3.231353,0.471107\C,0,1.804079,-2.559001,-1.364303\C,0,2.078489,-4.336101,0.2945
35\C,0,1.876781,-5.301249,-0.691468\C,0,1.6469,-3.552551,-2.343807\C,0,1.686396,-
4.90759,-2.016046\H,0,1.463907,-3.258183,-3.369853\H,0,1.554539,-5.651901,-2.7948
38\H,0,2.247802,-4.6017,1.331856\H,0,1.881296,-6.353432,-0.425104\P,0,1.989428,0.
802016,1.502948\P,0,1.506007,-0.771238,-1.712006\C,0,1.699782,0.508181,3.287128\C,
0,2.425171,2.573899,1.388939\C,0,3.136388,-0.036179,-2.092053\C,0,0.660187,-0.789
986,-3.342456\C,0,4.272426,-0.796652,-2.384074\C,0,3.218933,1.364277,-2.093909\C,
0,4.429007,1.994193,-2.37535\C,0,5.483716,-0.161899,-2.658362\C,0,5.564937,1.2312
26,-2.653\H,0,4.213985,-1.87971,-2.380478\H,0,2.331422,1.949396,-1.886573\H,0,4.4
82848,3.078509,-2.366046\H,0,6.36652,-0.757821,-2.869618\H,0,6.511686,1.720444,-2.
861855\C,0,1.833376,-0.780926,3.818556\C,0,1.21361,1.542272,4.10121\C,0,0.88944,1.
292525,5.432523\C,0,1.038668,0.00951,5.962329\C,0,1.508231,-1.023439,5.152027\H,0,
1.07008,2.534058,3.691766\H,0,0.510237,2.099764,6.051802\H,0,0.777913,-0.185229,6.
998389\H,0,1.602015,-2.029647,5.548149\H,0,2.134217,-1.604396,3.186658\C,0,3.292,
3.182728,2.31155\C,0,1.881643,3.340424,0.350145\C,0,2.233276,4.683726,0.212306\C,
0,3.636015,4.526056,2.173847\C,0,3.112706,5.276604,1.117344\H,0,3.68915,2.607721,
3.141802\H,0,1.163262,2.899342,-0.331444\H,0,1.799139,5.266336,-0.594266\H,0,4.30
8443,4.986726,2.891265\H,0,3.380551,6.323836,1.011582\C,0,-0.47041,-1.598684,-3.5
3674\C,0,1.049612,0.083459,-4.365361\C,0,0.323337,0.143384,-5.553986\C,0,-1.19229
4,-1.536235,-4.724348\C,0,-0.798712,-0.661793,-5.73721\H,0,-0.792262,-2.279871,-2.
760045\H,0,1.9109,0.726335,-4.23429\H,0,0.633937,0.831673,-6.334012\H,0,-2.072385,
-2.160059,-4.84646\H,0,-1.368868,-0.602056,-6.659094\Pd,0,0.279258,0.339612,0.056
914\C,0,-2.286916,-1.089528,2.024632\C,0,-1.56149,0.153988,2.505445\C,0,-0.90018,
1.128859,1.541162\H,0,-0.777955,-0.183345,3.186572\H,0,-2.276781,0.748365,3.08604
1\C,0,-0.986929,2.41996,1.626903\C,0,-1.041542,3.732206,1.707013\H,0,-1.762855,4.

311096,1.13727\H,0,-0.351686,4.288862,2.339224\C,0,-3.663464,-0.911633,1.668165\C,
0,-1.617615,-2.329999,2.335801\O,0,-2.414388,-3.444879,2.421392\O,0,-4.404611,-2.
052289,1.5139\O,0,-4.177314,0.19697,1.451924\O,0,-0.397096,-2.425648,2.516165\C,0,
-1.777877,-4.631697,2.910112\C,0,-1.165791,-5.465861,1.793446\H,0,-2.573937,-5.18
6323,3.415905\H,0,-1.009897,-4.356936,3.637454\H,0,-1.925582,-5.761356,1.062585\H,
0,-0.719678,-6.379426,2.203197\H,0,-0.382223,-4.899115,1.287057\C,0,-5.771929,-1.
860102,1.136371\C,0,-6.642705,-1.543393,2.343658\H,0,-6.065236,-2.805423,0.670644
\H,0,-5.837705,-1.061931,0.39304\H,0,-6.538118,-2.319814,3.107533\H,0,-7.69698,-1.
483912,2.050429\H,0,-6.345317,-0.583788,2.771979\\Version=ES64L-G16RevA.03\State=
1-A\HF=-4149.3686749\RMSD=4.958e-09\Dipole=2.6013901,-1.6803139,-1.4634464\Quadru
pole=8.3906766,21.6338362,-30.0245128,5.6495095,10.8222081,10.9296469\PG=C01 [X(C
61H55N1O7P2Pd1S1)]\

TS-2

\0,1\C,0,-2.483285,-3.983077,2.28919\C,0,-3.696615,-3.701844,1.645646\C,0,-3.9696
22,-2.735825,0.689485\C,0,-1.268205,-3.3416,2.087122\C,0,-3.105756,-1.817851,0.06
6104\C,0,-0.910154,-2.384126,1.138104\C,0,-1.676412,-1.802739,0.106027\H,0,-2.502
529,-4.764717,3.043393\H,0,-4.545764,-4.305847,1.955044\H,0,-5.008958,-2.636321,0.
392932\H,0,-0.445552,-3.668783,2.719592\H,0,-3.56355,-1.221053,-0.704627\H,0,0.13
3507,-2.099214,1.133025\N,0,-0.975463,-1.094084,-0.832159\S,0,-1.378243,-1.103396,
-2.449655\O,0,-0.207155,-0.492023,-3.102352\O,0,-1.818128,-2.465415,-2.799685\C,0,
-5.06971,0.183811,-3.501154\C,0,-4.011164,-0.627028,-3.103803\C,0,-2.770051,-0.04
4759,-2.842588\C,0,-2.574852,1.325308,-2.993097\C,0,-3.647819,2.122287,-3.378037\
C,0,-4.907946,1.56862,-3.634872\H,0,-6.035314,-0.265274,-3.716868\H,0,-4.123675,-
1.70173,-3.02431\H,0,-1.596029,1.749778,-2.820397\H,0,-3.506071,3.193436,-3.48717
7\C,0,-6.070896,2.445938,-4.020976\H,0,-6.788247,1.907895,-4.647531\H,0,-5.739032,
3.335453,-4.563832\H,0,-6.60792,2.789819,-3.128458\O,0,2.554984,-0.627652,2.28056
4\C,0,3.705218,0.114948,2.040142\C,0,2.649854,-1.998263,2.095208\C,0,3.563125,1.3
12521,1.327876\C,0,4.940478,-0.318988,2.51509\C,0,6.077491,0.440466,2.252496\C,0,
4.726757,2.044722,1.056457\C,0,5.97233,1.616029,1.51085\H,0,4.661561,2.946487,0.4
62144\H,0,6.857864,2.197647,1.27697\H,0,7.043556,0.104088,2.615724\H,0,5.003235,-
1.249993,3.066736\C,0,2.678905,-2.531429,0.794295\C,0,2.666308,-2.808274,3.226525
\C,0,2.763421,-4.192263,3.079612\C,0,2.807582,-3.92407,0.677147\C,0,2.856311,-4.7
4609,1.802965\H,0,2.835578,-4.369312,-0.309538\H,0,2.949298,-5.820114,1.67843\H,0,
2.607858,-2.339844,4.203057\H,0,2.776491,-4.828895,3.958583\P,0,1.88444,1.823053,
0.754076\P,0,2.324993,-1.451409,-0.665591\C,0,1.141804,2.584894,2.252472\C,0,2.20
6373,3.204173,-0.39428\C,0,3.934789,-0.741842,-1.166311\C,0,1.999889,-2.685816,-1.
978753\C,0,5.166055,-1.224711,-0.712907\C,0,3.905167,0.322363,-2.07865\C,0,5.0899
1,0.908273,-2.517516\C,0,6.351359,-0.632738,-1.148868\C,0,6.316284,0.43395,-2.047
946\H,0,5.1969,-2.05096,-0.01117\H,0,2.95176,0.684146,-2.444019\H,0,5.051664,1.73
827,-3.216246\H,0,7.303341,-1.002758,-0.780428\H,0,7.241625,0.894699,-2.380417\C,
0,1.128152,1.877301,3.462712\C,0,0.448849,3.800345,2.166193\C,0,-0.237007,4.30102
6,3.270319\C,0,-0.246987,3.592449,4.472493\C,0,0.438619,2.381823,4.564304\H,0,0.4
22231,4.347063,1.233329\H,0,-0.77797,5.238141,3.182568\H,0,-0.793913,3.976647,5.3

27987\H,0,0.424309,1.815373,5.490209\H,0,1.620657,0.917493,3.540578\C,0,2.770479,
4.419574,0.023466\C,0,1.870782,3.034023,-1.743949\C,0,2.137666,4.041983,-2.669462
\C,0,3.027167,5.43073,-0.901292\C,0,2.719551,5.238551,-2.25059\H,0,3.000391,4.577
459,1.07227\H,0,1.372607,2.12174,-2.05563\H,0,1.86945,3.898043,-3.711417\H,0,3.46
4698,6.367148,-0.568956\H,0,2.920364,6.026988,-2.969779\C,0,0.895147,-3.539117,-1.
847453\C,0,2.807942,-2.780683,-3.11924\C,0,2.51199,-3.714911,-4.111299\C,0,0.6065
63,-4.47204,-2.83885\C,0,1.412676,-4.562142,-3.973471\H,0,0.251228,-3.468323,-0.9
79928\H,0,3.664785,-2.128643,-3.23728\H,0,3.142708,-3.777032,-4.993121\H,0,-0.269
226,-5.10296,-2.734788\H,0,1.178883,-5.282527,-4.75152\Pd,0,0.58796,0.195058,-0.2
05353\C,0,-3.146009,0.442643,1.277352\C,0,-1.758157,1.028371,1.470463\C,0,-0.9701
25,1.477763,0.254785\H,0,-1.180816,0.277854,2.019167\H,0,-1.821006,1.88417,2.1586
73\C,0,-1.11799,2.579901,-0.410462\C,0,-1.19491,3.713499,-1.07325\H,0,-1.833523,3.
830088,-1.943362\H,0,-0.656214,4.601272,-0.74787\C,0,-4.082958,1.143685,0.414815\
C,0,-3.597009,-0.246174,2.469514\O,0,-4.954967,-0.433874,2.561318\O,0,-5.152529,0.
364001,0.031716\O,0,-3.965266,2.29666,0.020056\O,0,-2.850758,-0.619691,3.375647\C,
0,-5.447848,-0.771281,3.865592\C,0,-5.457729,-2.268442,4.129735\H,0,-6.464534,-0.
366084,3.887345\H,0,-4.842099,-0.261907,4.620344\H,0,-6.026657,-2.793791,3.356686
\H,0,-5.924946,-2.474186,5.099752\H,0,-4.436373,-2.650677,4.140584\C,0,-6.295943,
1.07265,-0.457407\C,0,-7.184924,1.502605,0.701122\H,0,-6.8104,0.371762,-1.120353\
H,0,-5.962053,1.933477,-1.039314\H,0,-7.465349,0.639447,1.310611\H,0,-8.094795,1.
987408,0.329773\H,0,-6.650444,2.212603,1.338105\\Version=ES64L-G16RevA.03\State=1
-A\HF=-4149.3477973\RMSD=6.335e-09\Dipole=3.9096696,-0.7843086,1.1997025\Quadrupo
le=20.2486513,3.3200524,-23.5687037,9.3569521,8.3928291,-22.4676037\PG=C01 [X(C61
H55N1O7P2Pd1S1)]\

int-4b-boat

\0,1\C,0,3.857914,3.170282,2.460126\C,0,4.637479,2.766397,1.31643\C,0,4.304587,1.
854509,0.378587\C,0,2.507342,3.070583,2.591346\C,0,3.086674,0.968842,0.36973\C,0,
1.579639,2.614322,1.593165\C,0,1.817445,1.791404,0.538535\H,0,4.400249,3.670672,3.
258317\H,0,5.603197,3.258247,1.207133\H,0,4.982453,1.708169,-0.456566\H,0,2.06304
4,3.48575,3.493328\H,0,3.02274,0.547975,-0.628156\H,0,0.5675,3.000343,1.679386\N,
0,0.775003,1.580384,-0.402271\S,0,0.894038,2.142949,-1.929318\O,0,-0.315171,1.658
923,-2.63335\O,0,1.220725,3.580509,-1.922688\C,0,4.587286,1.474173,-3.485974\C,0,
3.504454,2.062388,-2.838341\C,0,2.285188,1.383996,-2.785966\C,0,2.138716,0.139419,
-3.393455\C,0,3.226436,-0.427316,-4.055816\C,0,4.465248,0.223708,-4.107019\H,0,5.
539202,1.997908,-3.521017\H,0,3.585229,3.038495,-2.375553\H,0,1.186431,-0.373014,
-3.348196\H,0,3.11057,-1.390003,-4.546757\C,0,5.634505,-0.386668,-4.840149\H,0,6.
566721,-0.258687,-4.280411\H,0,5.778295,0.087275,-5.818644\H,0,5.484917,-1.456309,
-5.012372\O,0,-2.768863,-0.37708,2.364698\C,0,-3.782651,-1.145771,1.80979\C,0,-3.
065255,0.944919,2.66447\C,0,-3.424058,-1.99609,0.756229\C,0,-5.084794,-1.08893,2.
299653\C,0,-6.068333,-1.87933,1.709105\C,0,-4.436463,-2.759596,0.162101\C,0,-5.74
7662,-2.704234,0.632115\H,0,-4.202887,-3.385472,-0.689332\H,0,-6.516102,-3.299965,
0.150577\H,0,-7.086314,-1.836962,2.083885\H,0,-5.32044,-0.41942,3.118747\C,0,-3.0
62438,1.910892,1.64425\C,0,-3.323861,1.26953,3.992565\C,0,-3.632093,2.586467,4.33

1697\C,0,-3.396843,3.224671,2.011361\C,0,-3.684914,3.559273,3.334453\H,0,-3.4105,
3.996679,1.25255\H,0,-3.936759,4.585057,3.583946\H,0,-3.28227,0.483088,4.738556\H,
0,-3.834508,2.845292,5.366149\P,0,-1.664918,-2.01503,0.208056\P,0,-2.475255,1.496
982,-0.062519\C,0,-0.856507,-3.125271,1.433485\C,0,-1.688011,-2.960178,-1.355303\
C,0,-3.910859,0.759629,-0.925344\C,0,-2.397638,3.167943,-0.816434\C,0,-5.2192,0.7
92877,-0.43512\C,0,-3.650915,0.159548,-2.166167\C,0,-4.684654,-0.427541,-2.891637
\C,0,-6.251696,0.199855,-1.161802\C,0,-5.986361,-0.415231,-2.385972\H,0,-5.42769,
1.268457,0.516773\H,0,-2.641065,0.173841,-2.559796\H,0,-4.470803,-0.898404,-3.846
239\H,0,-7.262823,0.212664,-0.766552\H,0,-6.792009,-0.881516,-2.945249\C,0,-1.013
745,-2.889578,2.806789\C,0,0.049467,-4.109706,1.013513\C,0,0.784228,-4.83908,1.94
5401\C,0,0.625312,-4.597415,3.310432\C,0,-0.279017,-3.625101,3.736182\H,0,0.19992
3,-4.293781,-0.041364\H,0,1.494221,-5.584199,1.600575\H,0,1.20851,-5.156865,4.035
269\H,0,-0.404415,-3.423171,4.795568\H,0,-1.679499,-2.111415,3.154548\C,0,-2.0468
97,-4.315764,-1.414396\C,0,-1.323156,-2.298019,-2.535024\C,0,-1.35038,-2.967551,-
3.757469\C,0,-2.066386,-4.986195,-2.636592\C,0,-1.725198,-4.310173,-3.810679\H,0,
-2.298815,-4.849006,-0.503344\H,0,-0.995992,-1.264574,-2.483185\H,0,-1.063119,-2.
442202,-4.663104\H,0,-2.345136,-6.034977,-2.671927\H,0,-1.740794,-4.833919,-4.761
844\C,0,-1.370126,4.041918,-0.440303\C,0,-3.369892,3.607532,-1.725471\C,0,-3.3101
56,4.899473,-2.246303\C,0,-1.313665,5.330981,-0.962865\C,0,-2.283866,5.7646,-1.86
5402\H,0,-0.590956,3.703456,0.227056\H,0,-4.172425,2.94678,-2.02908\H,0,-4.066812,
5.226863,-2.953265\H,0,-0.490598,5.980288,-0.684575\H,0,-2.234307,6.76723,-2.2796
07\Pd,0,-0.564298,-0.017167,-0.106053\C,0,3.168795,-0.327674,1.264244\C,0,1.77504
7,-0.99864,1.373687\C,0,1.10654,-1.204572,0.030401\H,0,1.16495,-0.367444,2.020601
\H,0,1.896884,-1.957064,1.884854\C,0,1.530823,-2.002847,-0.898904\C,0,1.922447,-2.
855916,-1.821199\H,0,2.608994,-2.567909,-2.61088\H,0,1.575691,-3.887916,-1.824946
\C,0,4.105984,-1.378284,0.642255\C,0,3.66241,-0.101624,2.700383\O,0,5.00879,-0.07
9562,2.756372\O,0,4.644306,-0.992011,-0.527419\O,0,4.297603,-2.464284,1.148446\O,
0,2.944509,-0.013576,3.673025\C,0,5.601046,-0.091375,4.073755\C,0,5.724849,1.3046
53,4.658433\H,0,6.580262,-0.55156,3.922206\H,0,4.995625,-0.732855,4.718904\H,0,6.
294245,1.955701,3.989685\H,0,6.243649,1.255827,5.62184\H,0,4.735892,1.738643,4.81
32\C,0,5.458033,-1.967792,-1.211878\C,0,6.882151,-1.947434,-0.683103\H,0,5.406651,
-1.667286,-2.259279\H,0,4.996604,-2.949994,-1.089553\H,0,7.311477,-0.945126,-0.77
2042\H,0,7.505317,-2.643926,-1.253902\H,0,6.898946,-2.245908,0.367345\\Version=ES
64L-G16RevA.03\State=1-A\HF=-4149.3843689\RMSD=2.226e-09\Dipole=-2.1670864,-2.290
1448,0.4905894\Quadrupole=29.295175,-21.5114133,-7.7837617,2.038258,-13.5844713,2
6.8527985\PG=C01 [X(C61H55N1O7P2Pd1S1)]\

TS-3b-boat

\0,1\C,0,3.505501,3.435168,2.525332\C,0,4.590521,2.824445,1.785003\C,0,4.546007,1.
798675,0.913247\C,0,2.178492,3.355444,2.259382\C,0,3.369868,0.960565,0.501058\C,0,
1.534654,2.649171,1.178948\C,0,2.043976,1.684535,0.382772\H,0,3.806522,4.072456,3.
353832\H,0,5.571705,3.25941,1.969959\H,0,5.478017,1.504911,0.437679\H,0,1.508379,
3.926052,2.897694\H,0,3.595156,0.618635,-0.505513\H,0,0.498476,2.912252,0.987644\
N,0,1.213181,1.106902,-0.62783\S,0,1.324096,1.740414,-2.183635\O,0,0.39744,0.9522

54,-3.006103\O,0,1.195224,3.201746,-2.084686\C,0,5.319983,2.014897,-2.703909\C,0,
4.001915,2.324625,-2.385124\C,0,2.98994,1.42942,-2.743788\C,0,3.276051,0.262041,-
3.446452\C,0,4.602091,-0.026778,-3.764442\C,0,5.641814,0.829238,-3.381445\H,0,6.1
12939,2.701558,-2.420735\H,0,3.751558,3.23519,-1.854324\H,0,2.469847,-0.398539,-3.
733985\H,0,4.831909,-0.933345,-4.317975\C,0,7.08012,0.479316,-3.668937\H,0,7.6535
3,1.359559,-3.975027\H,0,7.16,-0.272342,-4.458853\H,0,7.567152,0.071868,-2.774019
\O,0,-2.800203,-0.441959,2.30824\C,0,-3.803093,-1.254692,1.79802\C,0,-3.129216,0.
862328,2.652868\C,0,-3.463896,-2.091681,0.723566\C,0,-5.07893,-1.254141,2.35701\C,
0,-6.061457,-2.080128,1.816726\C,0,-4.480024,-2.890073,0.181964\C,0,-5.766031,-2.
887667,0.719278\H,0,-4.267866,-3.504042,-0.68408\H,0,-6.535034,-3.51166,0.275516\
H,0,-7.05891,-2.079081,2.245502\H,0,-5.296034,-0.596372,3.190902\C,0,-3.23256,1.8
58945,1.662836\C,0,-3.285847,1.151196,4.005006\C,0,-3.577692,2.454723,4.40706\C,0,
-3.534833,3.159982,2.095599\C,0,-3.71136,3.456817,3.447422\H,0,-3.617317,3.952383,
1.36103\H,0,-3.942804,4.474166,3.747178\H,0,-3.171019,0.346478,4.723421\H,0,-3.69
8583,2.680872,5.461791\P,0,-1.753188,-1.991682,0.044906\P,0,-2.774453,1.500056,-0.
09169\C,0,-0.781488,-3.086937,1.1497\C,0,-1.80817,-2.922837,-1.531992\C,0,-4.2603
89,0.714593,-0.827109\C,0,-2.844458,3.167643,-0.860197\C,0,-5.531932,0.743318,-0.
245486\C,0,-4.08547,0.074834,-2.062917\C,0,-5.16282,-0.533073,-2.702302\C,0,-6.60
9721,0.130013,-0.885116\C,0,-6.428085,-0.508803,-2.112105\H,0,-5.675141,1.231562,
0.712665\H,0,-3.098818,0.047549,-2.512226\H,0,-5.010092,-1.034662,-3.653169\H,0,-
7.590321,0.144481,-0.418769\H,0,-7.267936,-0.991264,-2.603264\C,0,-0.885326,-2.96
4038,2.544919\C,0,0.234487,-3.894472,0.614987\C,0,1.13621,-4.548361,1.453801\C,0,
1.032835,-4.41265,2.837472\C,0,0.012655,-3.626683,3.378396\H,0,0.343695,-3.991178,
-0.456496\H,0,1.937333,-5.138511,1.021063\H,0,1.747433,-4.90592,3.48885\H,0,-0.07
4854,-3.51393,4.455018\H,0,-1.642304,-2.323825,2.97983\C,0,-2.109075,-4.292857,-1.
601725\C,0,-1.485014,-2.233956,-2.70944\C,0,-1.50078,-2.894034,-3.939124\C,0,-2.1
26294,-4.949728,-2.830731\C,0,-1.828607,-4.248264,-4.00256\H,0,-2.313115,-4.84654
6,-0.690677\H,0,-1.183705,-1.191877,-2.652753\H,0,-1.240748,-2.349655,-4.84175\H,
0,-2.364476,-6.008411,-2.874322\H,0,-1.838454,-4.76227,-4.959315\C,0,-1.637499,3.
851678,-1.040917\C,0,-4.042447,3.767966,-1.279383\C,0,-4.025377,5.037849,-1.85365
2\C,0,-1.621073,5.119369,-1.62208\C,0,-2.81514,5.716477,-2.024556\H,0,-0.701309,3.
383937,-0.769429\H,0,-4.983099,3.24042,-1.161802\H,0,-4.956365,5.495346,-2.176103
\H,0,-0.669768,5.617617,-1.777587\H,0,-2.805671,6.70081,-2.483651\Pd,0,-0.824398,
0.107217,-0.272703\C,0,3.157916,-0.396711,1.269223\C,0,1.691058,-0.843238,1.04923
2\C,0,1.226427,-0.69175,-0.387614\H,0,1.065901,-0.247649,1.715438\H,0,1.587099,-1.
882827,1.3502\C,0,1.646604,-1.505747,-1.343514\C,0,1.905014,-2.475301,-2.191539\H,
0,2.808693,-2.494031,-2.804943\H,0,1.229313,-3.321205,-2.344713\C,0,4.065846,-1.5
19801,0.754565\C,0,3.387124,-0.223884,2.770231\O,0,4.683145,-0.40557,3.088378\O,0,
4.897043,-1.123349,-0.228242\O,0,4.007138,-2.656754,1.172208\O,0,2.525014,0.06727
2,3.570339\C,0,5.02627,-0.265965,4.486661\C,0,5.230281,1.190604,4.867991\H,0,5.94
5124,-0.845957,4.596675\H,0,4.234469,-0.722519,5.085246\H,0,5.994902,1.651891,4.2
375\H,0,5.551662,1.257441,5.912735\H,0,4.298649,1.74746,4.751841\C,0,5.697551,-2.
155087,-0.844381\C,0,6.968668,-2.400571,-0.04956\H,0,5.906365,-1.768888,-1.843729
\H,0,5.092422,-3.061033,-0.922516\H,0,7.541624,-1.474634,0.058666\H,0,7.595671,-3.

137963,-0.561488\H,0,6.724252,-2.781624,0.944318\\Version=ES64L-G16RevA.03\State=1-A\HF=-4149.3211549\RMSD=5.195e-09\Dipole=0.2651699,-0.5888106,0.8388746\Quadrupole=38.1727722,-23.0526674,-15.1201049,1.8370566,-9.4743905,22.716331\PG=C01 [X(C61H55N1O7P2Pd1S1)]\

int-5

\0,1\C,0,-2.643022,-2.347084,4.074924\C,0,-3.700094,-1.402563,3.769994\C,0,-3.959491,-0.718861,2.640775\C,0,-1.759557,-2.890659,3.209447\C,0,-3.252054,-0.660083,1.312183\C,0,-1.639523,-2.651289,1.790068\C,0,-2.246882,-1.750706,0.996654\H,0,-2.589486,-2.666854,5.112907\H,0,-4.399625,-1.233297,4.587269\H,0,-4.845551,-0.091588,2.656678\H,0,-1.046941,-3.611791,3.600808\H,0,-4.040098,-0.734434,0.553359\H,0,-0.936173,-3.288247,1.27396\N,0,-1.843317,-1.770861,-0.384671\S,0,-2.701608,-2.823555,-1.430329\O,0,-1.857221,-3.081624,-2.596751\O,0,-3.203637,-3.917695,-0.597544\C,0,-6.359013,-1.107042,-1.650333\C,0,-5.310829,-1.956666,-1.300658\C,0,-4.1016,-1.86002,-1.98843\C,0,-3.935928,-0.942221,-3.027697\C,0,-4.989507,-0.093823,-3.355124\C,0,-6.21103,-0.155374,-2.667778\H,0,-7.307677,-1.186687,-1.126989\H,0,-5.417858,-2.691566,-0.510597\H,0,-2.995885,-0.896174,-3.563584\H,0,-4.865299,0.625442,-4.16009\C,0,-7.331823,0.79149,-3.016787\H,0,-7.124826,1.800187,-2.639229\H,0,-8.281357,0.464916,-2.584862\H,0,-7.460751,0.873828,-4.100431\O,0,3.686022,0.831563,1.216655\C,0,4.271046,1.361718,0.080379\C,0,4.214718,-0.344928,1.72732\C,0,3.460325,2.208865,-0.699026\C,0,5.589176,1.082252,-0.276757\C,0,6.127515,1.662037,-1.425116\C,0,4.036566,2.789843,-1.838295\C,0,5.356285,2.523077,-2.203082\H,0,3.432043,3.438678,-2.460795\H,0,5.773318,2.980516,-3.094771\H,0,7.150918,1.43399,-1.706444\H,0,6.181993,0.408606,0.329465\C,0,3.783719,-1.589015,1.236332\C,0,5.177638,-0.241157,2.730383\C,0,5.780622,-1.39214,3.232643\C,0,4.442127,-2.727632,1.729447\C,0,5.426665,-2.637768,2.711674\H,0,4.16836,-3.702955,1.344613\H,0,5.912055,-3.53952,3.072206\H,0,5.45398,0.746684,3.08378\H,0,6.534528,-1.313884,4.009803\P,0,1.643884,2.227801,-0.400873\P,0,2.385012,-1.662052,0.013757\C,0,1.382128,3.275579,1.085864\C,0,1.067494,3.393923,-1.707889\C,0,3.339858,-1.946186,-1.54101\C,0,1.632423,-3.313269,0.317487\C,0,3.955689,-3.169606,-1.839087\C,0,3.492697,-0.874024,-2.428811\C,0,4.261431,-1.013361,-3.584568\C,0,4.711907,-3.313723,-3.001005\C,0,4.870772,-2.234189,-3.874075\H,0,3.830631,-4.014333,-1.16938\H,0,3.00741,0.068201,-2.205922\H,0,4.380579,-0.166448,-4.53571\H,0,5.178562,-4.268547,-3.225848\H,0,5.464034,-2.348138,-4.776749\C,0,2.386118,3.601809,2.004235\C,0,0.07417,3.734258,1.317018\C,0,-0.219176,4.516021,2.430029\C,0,0.789243,4.84178,3.341649\C,0,2.0877,4.37842,3.127051\H,0,-0.726616,3.486345,0.6286\H,0,-1.240085,4.850611,2.585342\H,0,0.563679,5.448941,4.213593\H,0,2.877483,4.628428,3.830005\H,0,3.400156,3.256504,1.842747\C,0,1.251729,4.782689,-1.616556\C,0,0.427419,2.864283,-2.835361\C,0,-0.016348,3.706547,-3.857384\C,0,0.804737,5.621901,-2.63511\C,0,0.170165,5.085124,-3.759087\H,0,1.741529,5.20267,-0.744049\H,0,0.268875,1.793891,-2.901837\H,0,-0.512301,3.282934,-4.72619\H,0,0.950031,6.695254,-2.553134\H,0,-0.179079,5.741474,-4.550958\C,0,1.597701,-3.948831,1.567922\C,0,0.873755,-3.859288,-0.734172\C,0,0.13861,-5.02707,-0.550348\C,0,0.873841,-5.131089,1.744382\C,0,0.145983,-5.676752,0.687207\H,0,2.125872,-3.521242,2.412258\H,0,0.823779,-3.346943,-1.689001\H,0,-0.464378,-5.407309,-1.

367545\H,0,0.870024,-5.615315,2.717051\H,0,-0.432468,-6.583912,0.830617\Pd,0,0.74
868,0.030702,-0.268522\C,0,-2.598828,0.744781,0.979648\C,0,-2.163702,0.697857,-0.
522261\C,0,-1.348141,-0.52099,-0.912299\H,0,-1.621418,1.605935,-0.782272\H,0,-3.1
08016,0.699854,-1.086401\C,0,-0.395585,-0.501367,-1.915422\C,0,-0.065241,-0.70327
9,-3.18162\H,0,0.962716,-0.627669,-3.520186\H,0,-0.807806,-1.00922,-3.915251\C,0,
-3.570823,1.924014,1.003961\C,0,-1.483397,0.964614,1.997794\O,0,-1.941948,1.58206
3,3.09227\O,0,-4.856261,1.538346,0.900598\O,0,-3.220762,3.085298,0.983027\O,0,-0.
346135,0.541692,1.905085\C,0,-0.990146,1.769002,4.175549\C,0,-0.928122,0.541583,5.
065423\H,0,-1.367185,2.643767,4.707819\H,0,-0.017949,2.006152,3.7438\H,0,-1.91203
2,0.308472,5.480373\H,0,-0.234295,0.726778,5.892215\H,0,-0.576698,-0.325668,4.503
454\C,0,-5.825768,2.596043,0.721923\C,0,-7.194343,1.94643,0.713589\H,0,-5.604727,
3.110781,-0.218553\H,0,-5.710179,3.321317,1.531833\H,0,-7.256847,1.196149,-0.0781
24\H,0,-7.966361,2.70244,0.541849\H,0,-7.39883,1.455963,1.669519\\Version=ES64L-G
16RevA.03\State=1-A\HF=-4149.3556315\RMSD=6.148e-09\Dipole=-1.2104007,2.1951518,1.
1602521\Quadrupole=31.9773122,-34.3304824,2.3531702,-22.6527009,6.1392579,-14.367
0587\PG=C01 [X(C61H55N1O7P2Pd1S1)]\

product-boat

\0,1\C,0,0.338242,-1.315133,0.397862\C,0,0.380708,-2.651476,0.223441\C,0,1.505457,
-3.544218,0.355506\C,0,1.451621,-0.333176,0.691844\C,0,2.625481,-0.837891,1.48182
7\C,0,3.165474,-2.069967,1.531978\C,0,2.72057,-3.293305,0.899323\H,0,-0.556982,-3.
11313,-0.066757\H,0,1.330509,-4.54876,-0.020423\H,0,3.427439,-4.118969,0.929364\H,
0,3.098373,-0.065017,2.080322\H,0,4.053743,-2.175521,2.15269\N,0,-0.897946,-0.619
2,0.150639\C,0,-0.708959,0.623884,-0.544984\C,0,0.478021,0.540668,-1.476561\C,0,1.
793147,0.436254,-0.650946\H,0,0.521387,1.409606,-2.131488\H,0,0.399615,-0.359916,
-2.090772\H,0,0.988024,0.442768,1.308238\C,0,-1.420132,1.716564,-0.396959\C,0,-2.
107841,2.823857,-0.336176\H,0,-2.999968,2.978449,-0.941085\H,0,-1.831781,3.627793,
0.344041\C,0,2.310076,1.858949,-0.420015\C,0,2.829678,-0.367651,-1.431613\O,0,2.5
23113,2.631459,-1.3298\O,0,2.442931,2.171856,0.880989\C,0,2.738197,3.559748,1.177
57\C,0,1.45779,4.378226,1.172659\H,0,3.456201,3.92985,0.443352\H,0,3.20108,3.5331
56,2.165665\H,0,1.012715,4.381488,0.17488\H,0,1.673072,5.412461,1.458882\H,0,0.73
4866,3.964113,1.881214\O,0,2.568635,-1.209212,-2.262142\O,0,4.069704,-0.080283,-1.
00684\C,0,5.12406,-0.936913,-1.503618\C,0,6.385524,-0.568911,-0.751455\H,0,5.2208
31,-0.784562,-2.582545\H,0,4.82779,-1.976036,-1.334801\H,0,6.64707,0.479671,-0.91
7427\H,0,7.21819,-1.191569,-1.09186\H,0,6.249577,-0.726198,0.322006\S,0,-2.119171,
-0.710674,1.376398\O,0,-1.987062,0.399711,2.325918\O,0,-2.086182,-2.093668,1.8501
11\C,0,-3.581697,-0.449669,0.392186\C,0,-4.466061,0.563732,0.748584\C,0,-5.629257,
0.733947,0.001624\C,0,-5.913442,-0.092029,-1.092792\C,0,-7.183743,0.083264,-1.885
041\H,0,-7.948332,-0.630978,-1.556822\H,0,-7.015254,-0.089603,-2.951942\H,0,-7.59
7116,1.087469,-1.761377\C,0,-5.000417,-1.103567,-1.427175\C,0,-3.839359,-1.294525,
-0.687128\H,0,-4.225704,1.211636,1.582718\H,0,-6.324261,1.524731,0.268471\H,0,-5.
204441,-1.747349,-2.277838\H,0,-3.13223,-2.073285,-0.949088\\Version=ES64L-G16Rev
A.03\State=1-A\HF=-1873.8653385\RMSD=5.298e-09\Dipole=-0.3098016,0.8977509,-0.721
8644\Quadrupole=27.6916519,-4.7372742,-22.9543778,-8.1938464,18.8502504,11.042017

4\PG=C01 [X(C25H27N1O6S1)]\

int-4a-chair

\0,1\C,0,-3.601562,2.331477,-3.399765\C,0,-4.482122,2.551475,-2.27903\C,0,-4.3374
58,2.099837,-1.017176\C,0,-2.281985,2.016992,-3.314793\C,0,-3.287134,1.154286,-0.
482925\C,0,-1.489541,1.86935,-2.127043\C,0,-1.888272,1.584119,-0.858048\H,0,-4.01
5318,2.513445,-4.388329\H,0,-5.357602,3.168214,-2.480354\H,0,-5.069544,2.4169,-0.
281569\H,0,-1.726534,1.953399,-4.248403\H,0,-3.357203,1.243528,0.595444\H,0,-0.41
5125,1.934872,-2.277086\N,0,-0.885994,1.424122,0.12237\S,0,-1.020972,1.999653,1.6
43381\O,0,0.265468,1.69049,2.311913\O,0,-1.533604,3.37982,1.633113\C,0,-4.294557,
1.019749,3.860126\C,0,-3.361845,1.730295,3.104434\C,0,-2.237838,1.071971,2.610611
\C,0,-2.035361,-0.280749,2.887403\C,0,-2.980282,-0.981756,3.628567\C,0,-4.126022,
-0.343392,4.125936\H,0,-5.17152,1.53353,4.245116\H,0,-3.491821,2.783821,2.886262\
H,0,-1.150685,-0.783059,2.52223\H,0,-2.826128,-2.039694,3.822916\C,0,-5.137039,-1.
111767,4.939359\H,0,-6.073579,-0.556762,5.040444\H,0,-4.761227,-1.313146,5.94971\
H,0,-5.361422,-2.079661,4.479591\O,0,3.238915,-0.620069,-2.111264\C,0,4.155213,-1.
15977,-1.226103\C,0,3.473867,0.679173,-2.536966\C,0,3.633863,-1.856612,-0.129876\
C,0,5.528284,-1.053756,-1.439292\C,0,6.402717,-1.633526,-0.522794\C,0,4.530846,-2.
419461,0.785662\C,0,5.906718,-2.305296,0.595342\H,0,4.147129,-2.937239,1.656688\H,
0,6.588632,-2.736916,1.320477\H,0,7.473838,-1.548938,-0.678324\H,0,5.895907,-0.50
9784,-2.302058\C,0,3.237008,1.77212,-1.682196\C,0,3.922108,0.848377,-3.843984\C,0,
4.188061,2.129343,-4.325046\C,0,3.537909,3.048387,-2.187198\C,0,4.012848,3.22868,
-3.485781\H,0,3.375525,3.913693,-1.556968\H,0,4.230649,4.230465,-3.842036\H,0,4.0
57796,-0.033418,-4.460906\H,0,4.537231,2.262977,-5.344113\P,0,1.809637,-1.975234,
0.013562\P,0,2.415505,1.574098,-0.02888\C,0,1.428544,-3.446482,-1.01173\C,0,1.559
099,-2.587476,1.723616\C,0,3.775822,1.230814,1.148372\C,0,1.978181,3.314131,0.356
31\C,0,5.128002,1.309692,0.801872\C,0,3.418242,0.898612,2.464609\C,0,4.404253,0.6
18677,3.407652\C,0,6.111784,1.023371,1.748533\C,0,5.753203,0.670969,3.049443\H,0,
5.411805,1.584448,-0.20746\H,0,2.372386,0.898658,2.744682\H,0,4.117146,0.360701,4.
422612\H,0,7.159057,1.072538,1.465735\H,0,6.521279,0.444824,3.78318\C,0,2.31012,-
3.922577,-1.987241\C,0,0.187397,-4.080214,-0.84186\C,0,-0.161666,-5.168969,-1.633
992\C,0,0.721098,-5.636019,-2.612328\C,0,1.956625,-5.014427,-2.783463\H,0,-0.5064
86,-3.714738,-0.093373\H,0,-1.127249,-5.645468,-1.496367\H,0,0.444326,-6.480772,-
3.235817\H,0,2.649332,-5.373859,-3.53845\H,0,3.273657,-3.448364,-2.129827\C,0,1.7
67826,-3.929619,2.07574\C,0,1.159716,-1.674095,2.708039\C,0,0.973642,-2.093146,4.
02561\C,0,1.582667,-4.345365,3.393946\C,0,1.185952,-3.428378,4.370598\H,0,2.06367
9,-4.647224,1.317769\H,0,0.973646,-0.640458,2.436457\H,0,0.647277,-1.375461,4.771
998\H,0,1.74206,-5.386772,3.657135\H,0,1.03561,-3.757269,5.394577\C,0,0.944961,3.
928861,-0.363849\C,0,2.653271,4.044885,1.341641\C,0,2.2941,5.366714,1.604024\C,0,
0.591804,5.249031,-0.100923\C,0,1.26304,5.971352,0.885431\H,0,0.397623,3.374271,-
1.112913\H,0,3.452805,3.585051,1.909492\H,0,2.820461,5.920974,2.375547\H,0,-0.227
047,5.700365,-0.651551\H,0,0.978283,6.997244,1.099056\Pd,0,0.600021,-0.031406,-0.
23797\C,0,-3.533786,-0.401697,-0.706458\C,0,-2.292878,-1.312433,-0.383657\C,0,-0.
966173,-1.097097,-1.065918\C,0,-0.616406,-1.389087,-2.277831\C,0,-0.155726,-1.675

002,-3.47653\H,0,0.235742,-2.662229,-3.70863\H,0,-0.185472,-0.942988,-4.28006\C,0,-4.631717,-0.908771,0.254872\C,0,-4.066999,-0.74452,-2.106336\O,0,-5.407385,-0.560325,-2.160074\O,0,-5.369124,0.078141,0.791323\O,0,-4.815758,-2.085675,0.491211\O,0,-3.417433,-1.188083,-3.02344\C,0,-6.069724,-0.991276,-3.369208\C,0,-6.016122,0.069422,-4.454619\H,0,-7.096241,-1.192367,-3.053355\H,0,-5.604871,-1.920919,-3.707085\H,0,-6.441798,1.010633,-4.096964\H,0,-6.589009,-0.265411,-5.325982\H,0,-4.983473,0.246188,-4.758914\C,0,-6.524004,-0.33188,1.554499\C,0,-7.686125,-0.64036,0.624803\H,0,-6.733547,0.517808,2.206521\H,0,-6.25613,-1.197394,2.162709\H,0,-7.904392,0.219217,-0.014698\H,0,-8.580947,-0.880684,1.208254\H,0,-7.443905,-1.49542,-0.010066\H,0,-2.625583,-2.335133,-0.579883\H,0,-2.14691,-1.245324,0.691834\\Version=ES64L-G16RevA.03\State=1-A\HF=-4149.3665347\RMSD=6.300e-09\Dipole=1.9628119,-2.020662,0.5391908\Quadrupole=33.2292581,-26.9483225,-6.2809356,-2.5403802,-13.6530903,-15.7507231\PG=C01 [X(C61H55N1O7P2Pd1S1)]\

TS-3a-chair

\0,1\C,0,5.523279,-3.447001,-0.660184\C,0,5.970906,-2.151533,-0.199095\C,0,5.254071,-1.036666,0.034386\C,0,4.252457,-3.919075,-0.646502\C,0,3.785808,-0.749926,-0.147221\C,0,3.057805,-3.244913,-0.200703\C,0,2.837079,-1.926606,0.039878\H,0,6.306505,-4.131665,-0.978599\H,0,7.038824,-2.083284,0.004489\H,0,5.802074,-0.196163,0.455443\H,0,4.105403,-4.952845,-0.953733\H,0,3.539886,-0.080136,0.679907\H,0,2.19641,-3.879519,-0.013007\N,0,1.559431,-1.506846,0.458803\S,0,1.168336,-1.531761,2.033258\O,0,-0.105053,-0.781772,2.158683\O,0,1.239819,-2.88852,2.604499\C,0,4.624927,-0.429719,3.830017\C,0,3.568118,-1.199345,3.348134\C,0,2.433854,-0.558975,2.849029\C,0,2.351829,0.833195,2.815076\C,0,3.415344,1.586569,3.304839\C,0,4.564139,0.970392,3.819888\H,0,5.512136,-0.923636,4.217278\H,0,3.611346,-2.282182,3.34913\H,0,1.472272,1.312832,2.40599\H,0,3.348703,2.669882,3.281394\C,0,5.699089,1.791911,4.380542\H,0,6.672063,1.376269,4.099871\H,0,5.662288,1.815814,5.476427\H,0,5.656466,2.826343,4.028386\O,0,-3.661982,0.756058,-1.836572\C,0,-4.107153,1.703845,-0.931245\C,0,-4.397765,-0.406437,-2.01227\C,0,-3.124819,2.373528,-0.182101\C,0,-5.459383,2.010597,-0.806591\C,0,-5.856599,2.99897,0.091586\C,0,-3.550627,3.37446,0.700121\C,0,-4.903211,3.68132,0.845673\H,0,-2.816587,3.911926,1.288342\H,0,-5.207655,4.452942,1.54547\H,0,-6.911179,3.233447,0.198521\H,0,-6.186864,1.468093,-1.39968\C,0,-4.254269,-1.47825,-1.11008\C,0,-5.205568,-0.50575,-3.141135\C,0,-5.91005,-1.685298,-3.385084\C,0,-4.969837,-2.653469,-1.379566\C,0,-5.794393,-2.757146,-2.500621\H,0,-4.863704,-3.500238,-0.710929\H,0,-6.336297,-3.679262,-2.685813\H,0,-5.263155,0.342029,-3.815507\H,0,-6.541121,-1.764268,-4.264763\P,0,-1.369363,1.870223,-0.432393\P,0,-3.031116,-1.343878,0.257326\C,0,-1.027697,2.708985,-2.033432\C,0,-0.452772,2.866771,0.80282\C,0,-3.936659,-0.488626,1.603917\C,0,-2.86776,-3.039082,0.935284\C,0,-5.312871,-0.245017,1.577073\C,0,-3.181225,-0.081096,2.716019\C,0,-3.804698,0.571243,3.777718\C,0,-5.929255,0.418201,2.639626\C,0,-5.177487,0.82958,3.739219\H,0,-5.901176,-0.560349,0.722091\H,0,-2.115366,-0.285489,2.743251\H,0,-3.215714,0.88033,4.636636\H,0,-6.996578,0.615716,2.603481\H,0,-5.65834,1.347172,4.56407\C,0,-1.206716,4.092705,-2.184196\C,0,-0.624869,1.945098,-3.134879\C,0,-0.370619,2.560317,-4.362349\C,0,-0.529576,3.938651,-4.499731\C,0,-0.953736,4.704522,-3.

409604\H,0,-0.510915,0.872091,-3.028462\H,0,-0.042691,1.958794,-5.204283\H,0,-0.3
28403,4.417602,-5.453444\H,0,-1.08919,5.776852,-3.516559\H,0,-1.54219,4.68803,-1.
3404\C,0,0.652008,3.658179,0.455851\C,0,-0.778082,2.71759,2.163409\C,0,-0.054033,
3.392617,3.142528\C,0,1.386822,4.318167,1.442675\C,0,1.028841,4.201597,2.785638\H,
0,0.93452,3.767102,-0.584879\H,0,-1.598323,2.069543,2.451676\H,0,-0.324796,3.2738
54,4.187184\H,0,2.232054,4.936661,1.154891\H,0,1.59288,4.726477,3.550711\C,0,-1.6
48199,-3.706911,0.763293\C,0,-3.885672,-3.644528,1.688311\C,0,-3.693587,-4.911998,
2.233962\C,0,-1.452941,-4.968209,1.325789\C,0,-2.475985,-5.574469,2.053542\H,0,-0.
841761,-3.222824,0.221235\H,0,-4.817905,-3.115294,1.861074\H,0,-4.485749,-5.37602
6,2.814295\H,0,-0.491541,-5.458916,1.215582\H,0,-2.321461,-6.553664,2.497097\Pd,0,
-0.942287,-0.452917,-0.383154\C,0,3.408794,0.162778,-1.381548\C,0,1.948081,0.6741
14,-1.235113\C,0,0.948792,-0.428806,-1.185556\C,0,0.602126,-1.391707,-2.019002\C,
0,0.475839,-2.570326,-2.608073\H,0,-0.402572,-2.836187,-3.191569\H,0,1.269265,-3.
316228,-2.543229\C,0,4.353223,1.367025,-1.344309\C,0,3.562352,-0.46936,-2.768508\
O,0,4.489624,-1.428321,-2.786392\O,0,4.117579,2.112293,-0.242244\O,0,5.2125,1.615
18,-2.161128\O,0,2.922805,-0.109687,-3.735616\C,0,4.757994,-2.057243,-4.058175\C,
0,3.761604,-3.169908,-4.335904\H,0,5.773915,-2.443262,-3.953949\H,0,4.733056,-1.2
93341,-4.838664\H,0,3.766192,-3.892731,-3.515643\H,0,4.028801,-3.688831,-5.262419
\H,0,2.755345,-2.759961,-4.443975\C,0,5.007032,3.22457,-0.003405\C,0,6.291574,2.7
54022,0.658102\H,0,4.435378,3.885346,0.651471\H,0,5.206416,3.728321,-0.952164\H,0,
6.063657,2.18955,1.565761\H,0,6.914004,3.613502,0.927515\H,0,6.859498,2.118203,-0.
02496\H,0,1.714269,1.321211,-2.085023\H,0,1.874216,1.26888,-0.326107\\Version=ES6
4L-G16RevA.03\State=1-A\HF=-4149.3209659\RMSD=5.274e-09\Dipole=-1.3503746,2.04284
05,0.2048218\Quadrupole=21.8134389,0.4567556,-22.2701944,7.6676706,14.9880697,23.
8976779\PG=C01 [X(C61H55N1O7P2Pd1S1)]\

product-chair

\0,1\C,0,-1.908784,0.366522,-3.412557\C,0,-1.073552,1.407455,-2.848632\C,0,-0.436
688,1.451123,-1.666552\C,0,-2.081375,-0.89304,-2.952599\C,0,-0.360867,0.47375,-0.
52617\C,0,-1.510937,-1.504591,-1.772212\C,0,-0.785796,-0.959778,-0.774448\H,0,-2.
416312,0.628454,-4.337853\H,0,-0.938922,2.276124,-3.490291\H,0,0.175119,2.332518,
-1.486281\H,0,-2.717343,-1.552449,-3.537441\H,0,0.705133,0.431948,-0.27522\H,0,-1.
687867,-2.569569,-1.665242\N,0,-0.356663,-1.848173,0.270915\S,0,1.050135,-2.79536
1,-0.033462\O,0,1.353289,-3.50224,1.208982\O,0,0.812934,-3.496229,-1.294263\C,0,3.
389637,-0.008919,-1.790232\C,0,2.503186,-1.066124,-1.603993\C,0,2.323217,-1.57630
6,-0.317607\C,0,3.019035,-1.053326,0.77287\C,0,3.903793,0.000999,0.565064\C,0,4.1
00925,0.539484,-0.714252\H,0,3.532539,0.3973,-2.787435\H,0,1.948217,-1.490216,-2.
432113\H,0,2.863936,-1.474785,1.759572\H,0,4.454301,0.410956,1.406926\C,0,5.08667
7,1.657851,-0.937928\H,0,5.265471,2.224203,-0.020635\H,0,4.735398,2.352025,-1.707
041\H,0,6.052448,1.262867,-1.274921\C,0,-0.997384,0.955552,0.832409\C,0,-0.421772,
0.039798,1.944308\C,0,-0.623168,-1.423085,1.620472\H,0,-0.902937,0.265609,2.89577
1\H,0,0.647794,0.258929,2.02559\C,0,-1.038456,-2.302651,2.499462\C,0,-1.412771,-3.
169459,3.396408\H,0,-0.682911,-3.72833,3.977702\H,0,-2.46473,-3.37341,3.582108\C,
0,-0.599889,2.399329,1.128245\C,0,-2.527835,0.879091,0.843691\O,0,-3.045543,1.344

095,-0.299683\O,0,0.740247,2.545211,1.049724\O,0,-1.360603,3.296442,1.411298\O,0,
-3.175604,0.451929,1.773796\C,0,-4.489933,1.331228,-0.40441\C,0,-4.996067,-0.0415
22,-0.811839\H,0,-4.707546,2.084727,-1.163685\H,0,-4.909992,1.64547,0.553506\H,0,
-4.537104,-0.353595,-1.752869\H,0,-6.082584,-0.011583,-0.943041\H,0,-4.76107,-0.7
78221,-0.040848\C,0,1.258,3.867674,1.321755\C,0,2.745621,3.822765,1.041044\H,0,1.
037634,4.122301,2.36278\H,0,0.734726,4.587696,0.686396\H,0,3.235342,3.074298,1.67
0018\H,0,3.195686,4.798594,1.245256\H,0,2.933395,3.564558,-0.004335\\Version=ES64
L-G16RevA.03\State=1-A\HF=-1873.8691956\RMSD=4.836e-09\Dipole=1.4916599,2.8225693,
-0.9740495\Quadrupole=13.2368056,-16.044705,2.8078994,18.4303671,10.2629981,-6.09
01572\PG=C01 [X(C25H27N1O6S1)]\

int-3'

\0,1\C,0,6.217824,0.360853,0.706296\C,0,6.37039,1.720341,0.682153\C,0,5.579439,2.
702413,1.34573\C,0,5.23863,-0.37697,1.42436\C,0,4.396023,2.589744,2.02024\C,0,4.1
53757,0.074287,2.128589\C,0,3.599119,1.403137,2.236204\H,0,6.912837,-0.23227,0.12
0783\H,0,7.205834,2.107863,0.105187\H,0,5.96208,3.718037,1.265695\H,0,5.367837,-1.
455019,1.406845\H,0,3.961811,3.503124,2.412668\H,0,3.528901,-0.681985,2.590964\N,
0,2.315945,1.391458,2.587366\S,0,1.367076,2.715829,2.833217\O,0,0.102112,2.18799,
3.3824\O,0,2.043406,3.787171,3.593621\C,0,-0.43054,4.835726,-0.087754\C,0,-0.1357
84,4.212622,1.121489\C,0,0.974719,3.366777,1.204852\C,0,1.769407,3.123105,0.08770
9\C,0,1.467982,3.767827,-1.11144\C,0,0.373131,4.632667,-1.220399\H,0,-1.288703,5.
500451,-0.150471\H,0,-0.75841,4.37704,1.993122\H,0,2.596126,2.424972,0.088618\H,0,
2.094689,3.555416,-1.972193\C,0,0.031342,5.284417,-2.537381\H,0,-0.602577,4.62848
2,-3.147777\H,0,0.93096,5.499104,-3.120729\H,0,-0.514104,6.221556,-2.390608\O,0,-
2.523642,-1.8545,-2.100152\C,0,-3.765587,-1.928145,-1.503887\C,0,-1.584219,-2.877
591,-1.952828\C,0,-4.225986,-0.801609,-0.797825\C,0,-4.552921,-3.068448,-1.646827
\C,0,-5.810864,-3.111213,-1.052569\C,0,-5.488546,-0.877283,-0.196135\C,0,-6.27529
2,-2.020586,-0.316473\H,0,-5.845257,-0.043841,0.396566\H,0,-7.244136,-2.061115,0.
169875\H,0,-6.422394,-4.002346,-1.154026\H,0,-4.160822,-3.913077,-2.202917\C,0,-0.
834536,-3.021615,-0.762865\C,0,-1.286304,-3.619344,-3.088841\C,0,-0.195705,-4.492
752,-3.084442\C,0,0.271038,-3.884022,-0.79602\C,0,0.595866,-4.605048,-1.945431\H,
0,0.9349,-3.949369,0.055552\H,0,1.492266,-5.214255,-1.944631\H,0,-1.883443,-3.462
793,-3.980926\H,0,0.050148,-5.04911,-3.983369\P,0,-3.137048,0.672645,-0.661962\P,
0,-1.090893,-1.858687,0.636848\C,0,-3.638799,1.7184,-2.07961\C,0,-3.656881,1.6647
07,0.778161\C,0,-2.641847,-2.362693,1.479949\C,0,0.066119,-2.289201,1.977799\C,0,
-3.243497,-3.607676,1.276621\C,0,-3.151604,-1.501139,2.461296\C,0,-4.262094,-1.87
3258,3.213919\C,0,-4.355657,-3.978042,2.033284\C,0,-4.868092,-3.113096,2.999934\H,
0,-2.84954,-4.281941,0.523654\H,0,-2.678411,-0.542842,2.640047\H,0,-4.650857,-1.1
94813,3.967119\H,0,-4.822806,-4.943489,1.863155\H,0,-5.734706,-3.404417,3.585721\
C,0,-4.351548,1.211545,-3.170794\C,0,-3.218751,3.058839,-2.089562\C,0,-3.51806,3.
877502,-3.174875\C,0,-4.228741,3.366845,-4.265494\C,0,-4.642052,2.03555,-4.261079
\H,0,-2.654011,3.454978,-1.250743\H,0,-3.194388,4.913758,-3.17127\H,0,-4.457946,4.
005766,-5.112757\H,0,-5.19685,1.634226,-5.103785\H,0,-4.685389,0.179551,-3.169376
\C,0,-4.911432,2.291438,0.852268\C,0,-2.718206,1.879954,1.797132\C,0,-3.040805,2.

67141,2.900289\C,0,-5.23461,3.075256,1.957964\C,0,-4.30315,3.259048,2.985192\H,0,-5.620837,2.187698,0.037025\H,0,-1.721762,1.455952,1.726749\H,0,-2.285741,2.825346,3.663825\H,0,-6.207785,3.553916,2.01296\H,0,-4.558335,3.876945,3.841043\C,0,0.666188,-1.231987,2.665109\C,0,0.197147,-3.60168,2.462281\C,0,0.959817,-3.848506,3.599219\C,0,1.403123,-1.48133,3.824849\C,0,1.560477,-2.786815,4.285153\H,0,0.570412,-0.211701,2.317704\H,0,-0.307124,-4.422622,1.963258\H,0,1.072962,-4.865761,3.961575\H,0,1.846573,-0.639127,4.344456\H,0,2.140201,-2.982383,5.182437\Pd,0,-0.918547,0.109349,-0.599315\C,0,3.656975,-1.012297,-1.09102\C,0,2.364838,-0.867562,-0.336118\C,0,1.297794,-0.166735,-1.057363\H,0,2.499268,-0.313209,0.603837\H,0,2.029387,-1.867706,-0.060282\C,0,0.757469,0.6047,-1.882949\C,0,-0.35161,1.311681,-2.335595\H,0,-0.405643,2.375485,-2.133698\H,0,-0.881431,0.973382,-3.223433\C,0,4.238006,0.197341,-1.568939\C,0,4.156809,-2.352118,-1.153475\O,0,5.429825,-2.478377,-1.657725\O,0,5.402066,0.07441,-2.287067\O,0,3.758753,1.326824,-1.365804\O,0,3.547518,-3.354849,-0.744653\C,0,5.972881,-3.800968,-1.641757\C,0,6.529294,-4.157651,-0.268612\H,0,6.765811,-3.787205,-2.395698\H,0,5.204715,-4.520358,-1.938052\H,0,7.269439,-3.416259,0.05095\H,0,7.015217,-5.139652,-0.290039\H,0,5.715721,-4.188104,0.459837\C,0,5.931885,1.286862,-2.823464\C,0,7.382157,1.021805,-3.187168\H,0,5.833624,2.09332,-2.092238\H,0,5.349816,1.581743,-3.707113\H,0,7.96215,0.762402,-2.295366\H,0,7.83101,1.909201,-3.645859\H,0,7.460324,0.188319,-3.890979\\Version=ES64L-G16RevA.03\State=1-A\HF=-4149.3552065\RMSD=8.895e-09\Dipole=-4.6913187,-0.8016866,-2.1024201\Quadrupole=26.2576942,-0.6423112,-25.615383,6.7220593,5.3973775,-20.9716837\PG=C01 [X(C61H55N1O7P2Pd1S1)]\

TS-2’

\0,1\C,0,6.74093,-0.103497,1.444019\C,0,6.878965,1.298364,1.533658\C,0,5.845972,2.207651,1.643758\C,0,5.599341,-0.850754,1.295681\C,0,4.453051,2.010478,1.673212\C,0,4.242369,-0.453614,1.038958\C,0,3.690385,0.856769,1.456935\H,0,7.66322,-0.674633,1.542399\H,0,7.891111,1.688879,1.597085\H,0,6.147207,3.244643,1.785095\H,0,5.737641,-1.927813,1.319683\H,0,3.884603,2.891457,1.944978\H,0,3.53446,-1.244257,1.265562\N,0,2.338148,0.77968,1.556739\S,0,1.400123,1.841247,2.335769\O,0,0.11379,1.134682,2.573066\O,0,2.0234,2.480024,3.512568\C,0,-0.53765,4.942623,0.592755\C,0,-0.189826,3.887468,1.432636\C,0,0.984639,3.170166,1.190126\C,0,1.81018,3.506815,0.119393\C,0,1.450868,4.570566,-0.709405\C,0,0.274679,5.298341,-0.494673\H,0,-1.452837,5.498585,0.782608\H,0,-0.829404,3.607953,2.261244\H,0,2.690218,2.921986,-0.114171\H,0,2.085933,4.813179,-1.556865\C,0,-0.143374,6.394823,-1.442202\H,0,-0.749536,5.988732,-2.262079\H,0,0.722877,6.887823,-1.892311\H,0,-0.744577,7.155685,-0.935182\O,0,-2.717996,-1.47113,-2.240316\C,0,-3.94938,-1.608929,-1.624899\C,0,-1.868853,-2.571196,-2.323482\C,0,-4.333168,-0.618402,-0.706259\C,0,-4.789142,-2.672891,-1.946669\C,0,-6.028579,-2.77873,-1.321444\C,0,-5.574998,-0.761232,-0.074556\C,0,-6.416382,-1.830465,-0.375015\H,0,-5.873671,-0.043861,0.679292\H,0,-7.368586,-1.924847,0.136227\H,0,-6.683238,-3.609899,-1.564341\H,0,-4.455584,-3.412799,-2.665769\C,0,-1.101832,-2.983469,-1.212926\C,0,-1.71571,-3.160179,-3.573697\C,0,-0.778027,-4.178146,-3.752817\C,0,-0.152354,-3.994023,-1.426303\C,0,0.007551,-4.588886,-2.678442\H,0,0.496244,-4.293925,-0.612879\H,0,0.760231,-5.358981,-2.81055\H,0,-2.318

584,-2.792049,-4.396963\H,0,-0.653738,-4.63091,-4.731338\P,0,-3.178854,0.772962,-0.360311\P,0,-1.158929,-2.034631,0.366279\C,0,-3.609983,2.002162,-1.654761\C,0,-3.767358,1.593076,1.159856\C,0,-2.696753,-2.532203,1.231251\C,0,0.055992,-2.814883,1.485587\C,0,-3.450162,-3.657006,0.88544\C,0,-3.055368,-1.777046,2.356495\C,0,-4.170004,-2.130343,3.11209\C,0,-4.56658,-4.008532,1.644944\C,0,-4.930088,-3.246349,2.755323\H,0,-3.174931,-4.247713,0.018243\H,0,-2.459479,-0.916801,2.638586\H,0,-4.443428,-1.532447,3.976059\H,0,-5.155803,-4.876032,1.363233\H,0,-5.802203,-3.521246,3.340904\C,0,-4.134482,1.625071,-2.895699\C,0,-3.300328,3.351547,-1.419801\C,0,-3.517171,4.304988,-2.411065\C,0,-4.040598,3.923955,-3.64946\C,0,-4.347557,2.584606,-3.887819\H,0,-2.876981,3.651793,-0.467288\H,0,-3.275193,5.345374,-2.216071\H,0,-4.207559,4.667442,-4.422761\H,0,-4.757377,2.281931,-4.846735\H,0,-4.378146,0.58706,-3.091568\C,0,-5.01781,2.23044,1.227205\C,0,-2.902498,1.646109,2.25953\C,0,-3.301323,2.294492,3.430542\C,0,-5.412923,2.869528,2.400135\C,0,-4.557208,2.895917,3.506086\H,0,-5.668641,2.244832,0.3586\H,0,-1.90223,1.2286,2.197822\H,0,-2.614806,2.335026,4.270527\H,0,-6.381843,3.357336,2.448571\H,0,-4.865801,3.401094,4.416556\C,0,1.006,-2.002127,2.10832\C,0,-0.030303,-4.177075,1.823106\C,0,0.861039,-4.72312,2.741199\C,0,1.888324,-2.555138,3.040632\C,0,1.825115,-3.911441,3.349867\H,0,1.068056,-0.943882,1.884239\H,0,-0.799611,-4.802944,1.38161\H,0,0.797825,-5.777595,2.992704\H,0,2.619745,-1.910199,3.517481\H,0,2.514379,-4.339617,4.071641\Pd,0,-0.954633,0.160857,-0.396956\C,0,3.772474,-0.693743,-0.971086\C,0,2.268646,-1.027538,-1.022387\C,0,1.27109,0.044042,-1.084237\H,0,2.05145,-1.666315,-0.166355\H,0,2.083166,-1.671992,-1.892464\C,0,0.682283,1.057655,-1.51095\C,0,-0.431146,1.877108,-1.634714\H,0,-0.471053,2.816039,-1.096304\H,0,-0.993337,1.85237,-2.565333\C,0,4.232596,0.6015,-1.50279\C,0,4.505467,-1.960509,-1.213463\O,0,5.784566,-1.833252,-1.623576\O,0,5.574469,0.721235,-1.547232\O,0,3.497811,1.544049,-1.77808\O,0,3.991167,-3.057149,-1.014823\C,0,6.501301,-3.060804,-1.855892\C,0,7.074219,-3.637478,-0.570623\H,0,7.296214,-2.778074,-2.550497\H,0,5.834168,-3.779412,-2.338701\H,0,7.694407,-2.89601,-0.059455\H,0,7.694367,-4.511562,-0.797101\H,0,6.267661,-3.950175,0.09575\C,0,6.079141,2.024912,-1.876438\C,0,7.583092,1.891481,-2.011344\H,0,5.806048,2.7178,-1.073972\H,0,5.607822,2.375679,-2.799527\H,0,8.015744,1.537443,-1.072522\H,0,8.026859,2.861164,-2.259164\H,0,7.838965,1.178852,-2.800842\\Version=ES64L-G16RevA.03\State=1-A\HF=-4149.3424035\RMSD=3.390e-09\Dipole=-5.0224054,-1.7250439,-3.2623857\Quadrupole=18.2402498,10.0029785,-28.2432283,-5.5911235,-10.3397168,-7.5332056\PG=C01 [X(C61H55N1O7P2Pd1S1)]\

int-4a'

\0,1\C,0,-5.643291,-2.020421,-1.732233\C,0,-6.371932,-0.802848,-1.94103\C,0,-5.819476,0.443299,-2.000554\C,0,-4.37444,-2.202069,-1.301499\C,0,-4.468812,0.857747,-1.807689\C,0,-3.371672,-1.238071,-0.728093\C,0,-3.388701,0.188027,-1.273053\H,0,-6.170564,-2.929368,-2.025488\H,0,-7.428501,-0.901152,-2.178667\H,0,-6.493507,1.253023,-2.280273\H,0,-3.996776,-3.22095,-1.339915\H,0,-4.271608,1.874074,-2.12992\H,0,-2.396741,-1.623623,-1.03172\N,0,-2.135275,0.705602,-1.102885\S,0,-1.637694,2.091546,-1.732424\O,0,-0.198905,2.213212,-1.347543\O,0,-1.9219,2.28007,-3.174886\C,0,-3.467618,5.610966,-0.839732\C,0,-2.795006,4.597347,-1.52129\C,0,-2.508156,3.40

5156,-0.860076\C,0,-2.899754,3.212225,0.465262\C,0,-3.576882,4.231069,1.13056\C,0,
-3.870239,5.443999,0.491559\H,0,-3.691808,6.543447,-1.351568\H,0,-2.50707,4.71048,
-2.560764\H,0,-2.697781,2.273046,0.963578\H,0,-3.887123,4.074192,2.160429\C,0,-4.
636038,6.526978,1.210853\H,0,-4.445994,7.51062,0.771903\H,0,-4.368056,6.56849,2.2
71035\H,0,-5.71713,6.347802,1.156757\O,0,3.038729,-1.388592,2.078261\C,0,4.234089,
-1.402228,1.380415\C,0,2.218968,-2.5122,2.059974\C,0,4.545864,-0.288028,0.583246\
C,0,5.107757,-2.479483,1.508591\C,0,6.307807,-2.475766,0.804051\C,0,5.752172,-0.3
19419,-0.12852\C,0,6.624826,-1.40028,-0.025204\H,0,5.995828,0.496131,-0.797133\H,
0,7.544663,-1.404691,-0.600318\H,0,6.985668,-3.318986,0.891926\H,0,4.826685,-3.31
5493,2.139582\C,0,1.444318,-2.835387,0.922566\C,0,2.088922,-3.215638,3.251767\C,0,
1.16253,-4.255469,3.349722\C,0,0.508824,-3.872368,1.056537\C,0,0.365573,-4.574898,
2.253804\H,0,-0.163609,-4.100741,0.241346\H,0,-0.389832,-5.349776,2.325484\H,0,2.
696837,-2.914694,4.0982\H,0,1.053927,-4.793687,4.285958\P,0,3.339864,1.100577,0.4
53146\P,0,1.485192,-1.701823,-0.522821\C,0,3.774075,2.160802,1.885669\C,0,3.83527
1,2.116459,-0.979344\C,0,2.996122,-2.116573,-1.479145\C,0,0.239006,-2.183015,-1.7
65202\C,0,3.678228,-3.327236,-1.33414\C,0,3.381482,-1.221876,-2.487369\C,0,4.4434
24,-1.531397,-3.332087\C,0,4.742993,-3.634557,-2.18249\C,0,5.127211,-2.740029,-3.
180974\H,0,3.383379,-4.024634,-0.556885\H,0,2.851355,-0.283918,-2.608969\H,0,4.73
6395,-0.827882,-4.105412\H,0,5.272905,-4.574494,-2.059717\H,0,5.95618,-2.982826,-
3.838889\C,0,4.300963,1.624723,3.066611\C,0,3.473235,3.530712,1.826689\C,0,3.7058
68,4.349094,2.929589\C,0,4.234187,3.80994,4.105027\C,0,4.529075,2.448277,4.170093
\H,0,3.055381,3.95284,0.918221\H,0,3.471859,5.407708,2.871441\H,0,4.413264,4.4483
48,4.964664\H,0,4.940712,2.022663,5.080322\H,0,4.532983,0.568044,3.12942\C,0,5.07
4945,2.774435,-1.043717\C,0,2.902075,2.2926,-2.010933\C,0,3.228075,3.078712,-3.11
8071\C,0,5.396077,3.551887,-2.15379\C,0,4.475861,3.696286,-3.196747\H,0,5.775938,
2.695046,-0.218726\H,0,1.907013,1.865098,-1.927228\H,0,2.493345,3.217619,-3.90476
3\H,0,6.357399,4.054616,-2.201022\H,0,4.72674,4.308227,-4.05809\C,0,-0.33599,-1.1
13655,-2.462712\C,0,-0.04981,-3.500631,-2.158105\C,0,-0.973032,-3.731704,-3.17458
1\C,0,-1.248366,-1.348456,-3.492683\C,0,-1.584338,-2.656851,-3.831272\H,0,-0.0971
04,-0.100144,-2.174581\H,0,0.452758,-4.341203,-1.692686\H,0,-1.213866,-4.751536,-
3.458969\H,0,-1.716028,-0.49928,-3.979702\H,0,-2.317802,-2.845227,-4.609001\Pd,0,
1.163017,0.390194,0.453591\C,0,-3.244315,-1.289999,0.87941\C,0,-1.762921,-1.29401,
1.362781\C,0,-0.955327,-0.072193,1.308997\H,0,-1.272979,-2.087704,0.803423\H,0,-1.
746318,-1.616308,2.410077\C,0,-0.508752,1.02454,1.705793\C,0,0.427588,2.052667,1.
677754\H,0,0.222914,2.914446,1.046819\H,0,1.049454,2.225166,2.551864\C,0,-4.02121
2,-0.169254,1.561277\C,0,-3.747208,-2.677078,1.304245\O,0,-4.943805,-2.648517,1.9
03409\O,0,-5.244273,-0.038142,1.054434\O,0,-3.560358,0.532365,2.444376\O,0,-3.103
414,-3.693327,1.118987\C,0,-5.5359,-3.926543,2.23486\C,0,-6.242532,-4.520517,1.02
7777\H,0,-6.236325,-3.695245,3.040032\H,0,-4.754174,-4.591559,2.609459\H,0,-6.958
413,-3.805998,0.614402\H,0,-6.776646,-5.430733,1.32008\H,0,-5.519915,-4.773567,0.
249594\C,0,-6.036196,1.08241,1.501012\C,0,-7.492235,0.717625,1.293104\H,0,-5.7389
73,1.94713,0.899032\H,0,-5.799143,1.289659,2.547028\H,0,-7.670994,0.467556,0.2453
2\H,0,-8.132787,1.559302,1.57568\H,0,-7.762742,-0.147087,1.906393\\Version=ES64L-
G16RevA.03\State=1-A\HF=-4149.353043\RMSD=6.747e-09\Dipole=4.4862987,-1.7686373,2.

8279939\Quadrupole=15.5984029,11.5712275,-27.1696304,-1.4568611,-17.0925541,-0.80 63009\PG=C01 [X(C61H55N1O7P2Pd1S1)]\

TS-3a'

\0,1\C,0,-6.160248,1.388861,-2.407878\C,0,-6.4489,0.430877,-1.364599\C,0,-5.60757 7,-0.21084,-0.533475\C,0,-4.988601,2.037243,-2.61669\C,0,-4.106492,-0.201523,-0.4 32179\C,0,-3.741844,1.913702,-1.904365\C,0,-3.35602,1.001666,-0.97767\H,0,-6.9983 02,1.654372,-3.048418\H,0,-7.507626,0.219372,-1.220035\H,0,-6.074629,-0.838885,0. 223499\H,0,-4.973003,2.773819,-3.417896\H,0,-3.888574,-0.182911,0.644088\H,0,-2.9 92923,2.655956,-2.159728\N,0,-2.068031,1.015722,-0.387622\S,0,-1.441051,2.416046, 0.172402\O,0,-0.229342,2.054781,0.945353\O,0,-1.276233,3.505534,-0.816839\C,0,-4. 271507,4.588984,2.105217\C,0,-3.276741,4.207941,1.203991\C,0,-2.711557,2.942787,1. 319573\C,0,-3.123194,2.055758,2.317144\C,0,-4.115331,2.451132,3.206215\C,0,-4.707 047,3.72109,3.112501\H,0,-4.72057,5.574613,2.019654\H,0,-2.939401,4.86992,0.41513 2\H,0,-2.679244,1.069106,2.378637\H,0,-4.443328,1.764841,3.982741\C,0,-5.809481,4. 122711,4.059933\H,0,-6.766425,3.68187,3.755238\H,0,-5.940049,5.207797,4.084646\H, 0,-5.604976,3.779892,5.079019\O,0,3.862043,-1.681088,-0.649047\C,0,4.15131,-1.789 776,0.698588\C,0,4.645659,-0.899763,-1.479957\C,0,3.039405,-1.776581,1.552753\C,0, 5.444592,-1.951304,1.180287\C,0,5.639479,-2.070189,2.557144\C,0,3.257123,-1.88885 7,2.927796\C,0,4.552727,-2.028728,3.43001\H,0,2.409409,-1.86432,3.603735\H,0,4.70 9897,-2.107455,4.500795\H,0,6.646416,-2.187217,2.94573\H,0,6.280502,-1.961896,0.4 89686\C,0,4.385512,0.479183,-1.600708\C,0,5.604277,-1.536276,-2.26255\C,0,6.33810 3,-0.800632,-3.192763\C,0,5.124054,1.191221,-2.557571\C,0,6.09271,0.563245,-3.341 333\H,0,4.930084,2.247278,-2.698495\H,0,6.646705,1.142252,-4.073301\H,0,5.744141, -2.605283,-2.143273\H,0,7.085079,-1.295758,-3.805101\P,0,1.394851,-1.634707,0.752 449\P,0,3.020625,1.249345,-0.630192\C,0,1.335534,-3.25658,-0.117562\C,0,0.228176, -1.789147,2.162391\C,0,3.674045,1.386823,1.082842\C,0,3.064246,3.030132,-1.081832 \C,0,5.026684,1.246656,1.405303\C,0,2.75545,1.737374,2.083176\C,0,3.190343,1.9261 49,3.392932\C,0,5.455866,1.432181,2.719806\C,0,4.539785,1.769057,3.715677\H,0,5.7 4269,0.976329,0.636533\H,0,1.711598,1.876604,1.826294\H,0,2.472283,2.195246,4.161 978\H,0,6.50602,1.3042,2.964847\H,0,4.876127,1.908678,4.738797\C,0,1.859879,-4.40 9396,0.4894\C,0,0.787716,-3.346455,-1.403712\C,0,0.722043,-4.582042,-2.049961\C,0, 1.219561,-5.728788,-1.432077\C,0,1.797281,-5.639167,-0.163253\H,0,0.421088,-2.455 807,-1.908854\H,0,0.276447,-4.637965,-3.03806\H,0,1.166684,-6.688268,-1.938234\H, 0,2.202787,-6.525107,0.316772\H,0,2.329178,-4.34281,1.465959\C,0,-0.241019,-3.017 132,2.647545\C,0,-0.226198,-0.599109,2.752691\C,0,-1.109379,-0.643743,3.831752\C, 0,-1.13279,-3.055179,3.719948\C,0,-1.565426,-1.87058,4.317586\H,0,0.076467,-3.942 378,2.181791\H,0,0.072694,0.356721,2.338568\H,0,-1.450004,0.283983,4.281619\H,0,- 1.487456,-4.013545,4.088173\H,0,-2.259284,-1.903195,5.152507\C,0,1.896226,3.66217 5,-1.51466\C,0,4.224079,3.798308,-0.876265\C,0,4.219546,5.163292,-1.147151\C,0,1. 888417,5.035718,-1.76648\C,0,3.049728,5.785283,-1.596029\H,0,0.98169,3.099819,-1. 615714\H,0,5.124347,3.33186,-0.489774\H,0,5.122732,5.746192,-0.992359\H,0,0.96308 5,5.508699,-2.078885\H,0,3.045196,6.853332,-1.79305\Pd,0,0.984983,0.11225,-0.7586 36\C,0,-3.407682,-1.547158,-0.872698\C,0,-1.908385,-1.512809,-0.45944\C,0,-1.1500

72,-0.40353,-1.108759\C,0,-0.527915,-0.256279,-2.292219\C,0,0.094505,0.961431,-2.
631856\H,0,0.775031,1.010095,-3.479656\H,0,-0.372493,1.911769,-2.365221\C,0,-4.11
3868,-2.683678,-0.133918\C,0,-3.454397,-1.877225,-2.368802\O,0,-4.212111,-1.02013
1,-3.056021\O,0,-3.792399,-2.671024,1.179641\O,0,-4.901009,-3.458653,-0.629484\O,
0,-2.832276,-2.799374,-2.85148\C,0,-4.181692,-1.127435,-4.496087\C,0,-2.999136,-0.
34663,-5.046511\H,0,-5.135279,-0.704039,-4.816819\H,0,-4.132382,-2.183355,-4.7706
04\H,0,-3.058484,0.699539,-4.73501\H,0,-2.9962,-0.389443,-6.140874\H,0,-2.060617,
-0.765279,-4.674698\C,0,-4.464244,-3.636376,2.017418\C,0,-5.832521,-3.126929,2.43
9579\H,0,-3.795768,-3.758736,2.87141\H,0,-4.543755,-4.579987,1.472833\H,0,-5.7459
9,-2.153248,2.931174\H,0,-6.295561,-3.828587,3.14109\H,0,-6.486422,-3.029541,1.56
9819\H,0,-1.45965,-2.463532,-0.748282\H,0,-1.85646,-1.412688,0.621783\\Version=ES
64L-G16RevC.01\State=1-A\HF=-4149.3286658\RMSD=3.051e-09\Dipole=2.5449026,-0.0230
098,3.3298234\Quadrupole=20.5437768,-18.5026365,-2.0411403,-12.8976426,-21.856164
9,-14.9952362\PG=C01 [X(C61H55N1O7P2Pd1S1)]\

int-5'

\0,1\C,0,2.721427,2.292998,4.087565\C,0,3.781153,1.359726,3.758\C,0,4.032416,0.69
2619,2.616995\C,0,1.826108,2.845093,3.239632\C,0,3.307276,0.645765,1.29746\C,0,1.
693409,2.62664,1.818247\C,0,2.300191,1.7411,1.007367\H,0,2.676385,2.596235,5.1308
84\H,0,4.490962,1.184552,4.5651\H,0,4.922518,0.070963,2.614034\H,0,1.113076,3.555
466,3.649443\H,0,4.084397,0.72507,0.527923\H,0,0.979426,3.265593,1.318453\N,0,1.8
84888,1.779208,-0.369798\S,0,2.720551,2.859318,-1.411624\O,0,1.856259,3.130952,-2.
560106\O,0,3.229795,3.941088,-0.567969\C,0,6.366339,1.130608,-1.696343\C,0,5.3282
81,1.984082,-1.326404\C,0,4.110341,1.901123,-2.000369\C,0,3.924984,0.993043,-3.04
5004\C,0,4.968665,0.140142,-3.391997\C,0,6.199017,0.188144,-2.719406\H,0,7.321814,
1.199195,-1.183998\H,0,5.449442,2.710352,-0.530409\H,0,2.978372,0.959149,-3.57029
2\H,0,4.829748,-0.571809,-4.201026\C,0,7.308823,-0.763348,-3.090165\H,0,7.097442,
-1.774587,-2.722052\H,0,8.265101,-0.449097,-2.664079\H,0,7.426384,-0.834287,-4.17
5914\O,0,-3.701473,-0.847816,1.219999\C,0,-4.287658,-1.384738,0.087656\C,0,-4.232
617,0.328103,1.729532\C,0,-3.476589,-2.22907,-0.695813\C,0,-5.608936,-1.112462,-0.
263561\C,0,-6.15115,-1.696423,-1.408121\C,0,-4.056977,-2.81436,-1.830678\C,0,-5.3
80035,-2.554619,-2.189112\H,0,-3.452791,-3.461188,-2.455626\H,0,-5.799494,-3.0155
7,-3.077871\H,0,-7.177332,-1.473865,-1.683729\H,0,-6.201931,-0.440893,0.344779\C,
0,-3.808516,1.576103,1.241209\C,0,-5.193461,0.219932,2.734639\C,0,-5.800328,1.367
036,3.240615\C,0,-4.470628,2.710894,1.738341\C,0,-5.452741,2.615543,2.722144\H,0,
-4.200443,3.687834,1.35454\H,0,-5.940815,3.514515,3.085909\H,0,-5.464872,-0.76965
9,3.086937\H,0,-6.55232,1.28358,4.019103\P,0,-1.656572,-2.241455,-0.410061\P,0,-2.
416635,1.67577,0.014012\C,0,-1.38108,-3.298731,1.066972\C,0,-1.080048,-3.388646,-
1.733335\C,0,-3.376016,2.003894,-1.528323\C,0,-1.645495,3.309724,0.363657\C,0,-3.
928497,3.255625,-1.831869\C,0,-3.598029,0.931484,-2.402581\C,0,-4.371819,1.101221,
-3.550662\C,0,-4.689123,3.428723,-2.987323\C,0,-4.916567,2.35097,-3.846931\H,0,-3.
750045,4.098882,-1.172563\H,0,-3.162631,-0.034375,-2.174969\H,0,-4.544509,0.25541
5,-4.209372\H,0,-5.105701,4.405278,-3.217174\H,0,-5.512542,2.488151,-4.744566\C,0,
-2.380968,-3.642537,1.983364\C,0,-0.06763,-3.743658,1.294285\C,0,0.234419,-4.5315

5,2.40054\C,0,-0.769849,-4.874613,3.310547\C,0,-2.073357,-4.423518,3.100627\H,0,0.
729945,-3.480891,0.607458\H,0,1.258816,-4.856956,2.552577\H,0,-0.537018,-5.485712,
4.177828\H,0,-2.859614,-4.686735,3.802718\H,0,-3.398925,-3.307128,1.824204\C,0,-1.
240209,-4.78089,-1.654069\C,0,-0.460923,-2.836985,-2.862359\C,0,-0.015203,-3.6612
09,-3.897913\C,0,-0.790714,-5.602307,-2.686161\C,0,-0.1781,-5.043759,-3.811577\H,
0,-1.713052,-5.217561,-0.780361\H,0,-0.319998,-1.763301,-2.918215\H,0,0.464044,-3.
221091,-4.767903\H,0,-0.917116,-6.678735,-2.613609\H,0,0.172887,-5.686174,-4.6140
32\C,0,-1.608601,3.910812,1.630883\C,0,-0.872818,3.873101,-0.668832\C,0,-0.124173,
5.026288,-0.4505\C,0,-0.86856,5.077081,1.843096\C,0,-0.128808,5.64203,0.804422\H,
0,-2.149835,3.469332,2.459706\H,0,-0.824637,3.386559,-1.637178\H,0,0.487094,5.421
176,-1.254505\H,0,-0.863264,5.534666,2.828589\H,0,0.460823,6.537203,0.975037\Pd,0,
-0.849552,-0.043187,-0.258623\C,0,2.642582,-0.753239,0.96519\C,0,2.176272,-0.6945
53,-0.527301\C,0,1.398053,0.544448,-0.919212\H,0,1.602513,-1.586475,-0.775045\H,0,
3.108247,-0.723909,-1.111584\C,0,0.40585,0.538939,-1.870779\C,0,0.022066,0.756961,
-3.11905\H,0,0.741352,1.052138,-3.880187\H,0,-1.019878,0.700882,-3.415282\C,0,3.6
09443,-1.936965,0.958324\C,0,1.543608,-0.976704,1.999304\O,0,2.009119,-1.636196,3.
068527\O,0,4.896221,-1.555498,0.852947\O,0,3.253376,-3.095715,0.91617\O,0,0.41837
3,-0.522781,1.935359\C,0,1.073492,-1.828061,4.163916\C,0,1.056254,-0.622538,5.085
382\H,0,1.43854,-2.724561,4.667747\H,0,0.087979,-2.031536,3.744961\H,0,2.053841,-
0.420269,5.483907\H,0,0.377077,-0.814095,5.922847\H,0,0.710943,0.265368,4.552736\
C,0,5.858986,-2.614141,0.646468\C,0,7.229502,-1.968802,0.625319\H,0,5.621959,-3.1
16824,-0.296609\H,0,5.75332,-3.348953,1.449162\H,0,7.282455,-1.208914,-0.157987\H,
0,7.996415,-2.724904,0.432433\H,0,7.450185,-1.490808,1.583958\\Version=ES64L-G16R
evA.03\State=1-A\HF=-4149.3568321\RMSD=6.319e-09\Dipole=1.3308937,-2.1018677,0.96
01396\Quadrupole=32.1573842,-34.8382178,2.6808336,-22.4051592,-6.829102,13.727977
4\PG=C01 [X(C61H55N1O7P2Pd1S1)]\

3a

\0,1\C,0,-3.486274,-0.992373,0.178666\C,0,-2.3681,-1.697193,0.521\C,0,-1.000518,-
1.303769,0.437771\C,0,-3.532792,0.334759,-0.338598\C,0,-0.397972,-0.110025,0.1174
52\C,0,-2.519784,1.230228,-0.494279\H,0,-4.442533,-1.497444,0.285464\H,0,-2.52494
7,-2.711717,0.879005\H,0,-0.307256,-2.100237,0.69598\H,0,-4.520235,0.681453,-0.63
6119\H,0,-2.775527,2.207989,-0.894616\C,0,-1.098108,1.179572,-0.136231\O,0,-0.479
16,2.248522,-0.113379\C,0,1.08525,-0.103193,0.044443\C,0,1.740525,-1.159187,-0.61
4659\C,0,1.867744,0.908056,0.629239\C,0,3.129635,-1.211621,-0.682888\H,0,1.148807,
-1.927855,-1.102179\C,0,3.258159,0.845654,0.568409\H,0,1.379921,1.732686,1.127681
\C,0,3.894913,-0.208678,-0.086638\H,0,3.61302,-2.029504,-1.208453\H,0,3.847087,1.
629014,1.0357\H,0,4.978871,-0.245947,-0.137557\\Version=ES64L-G16RevC.01\State=1-
A\HF=-576.8763672\RMSD=3.336e-09\Dipole=-1.0299779,-1.8544386,0.0892342\Quadrupol
e=11.542568,-5.4933449,-6.0492231,1.7692648,0.4518483,0.9203027\PG=C01 [X(C13H10O
1)]\

int-5-o

\0,1\C,0,5.463916,-1.860627,2.267054\C,0,5.74811,-0.495887,1.879879\C,0,5.027028,

0.338736,1.114038\C,0,4.322561,-2.560153,2.071704\C,0,3.713183,0.16604,0.410751\C,
0,3.089596,-2.190342,1.393763\C,0,2.863414,-1.051298,0.682304\H,0,6.260495,-2.369
025,2.805086\H,0,6.677342,-0.091779,2.27828\H,0,5.418943,1.346946,0.996586\H,0,4.
30754,-3.574793,2.462209\H,0,3.094753,1.007969,0.746296\O,0,-3.581433,2.031704,-0.
474546\C,0,-3.562052,2.175949,0.902126\C,0,-4.593624,1.28065,-1.051896\C,0,-2.291
513,2.246063,1.495555\C,0,-4.72751,2.277541,1.65738\C,0,-4.631036,2.445998,3.0385
74\C,0,-2.222716,2.423685,2.882494\C,0,-3.381896,2.519239,3.653108\H,0,-1.251037,
2.46877,3.360955\H,0,-3.306707,2.64821,4.728054\H,0,-5.537845,2.518499,3.631356\H,
0,-5.69356,2.21132,1.170757\C,0,-4.438851,-0.110382,-1.194053\C,0,-5.720383,1.944
96,-1.529565\C,0,-6.745643,1.219184,-2.135738\C,0,-5.488974,-0.817608,-1.799644\C,
0,-6.632698,-0.165431,-2.259996\H,0,-5.399539,-1.891109,-1.920824\H,0,-7.429056,-
0.739332,-2.723417\H,0,-5.775003,3.022724,-1.418495\H,0,-7.62599,1.734821,-2.5064
4\P,0,-0.830817,1.960204,0.418904\P,0,-2.847616,-0.919969,-0.723046\Pd,0,-0.73582
8,0.051402,-0.958625\C,0,3.756393,0.377609,-1.150667\C,0,2.298718,0.694876,-1.581
405\C,0,1.333644,-0.358411,-1.098227\H,0,2.065295,1.671968,-1.156864\H,0,2.227119,
0.756583,-2.668897\C,0,0.579745,-1.206311,-1.922073\C,0,0.658205,-2.148213,-2.851
501\H,0,1.626244,-2.539232,-3.162997\H,0,-0.225195,-2.558146,-3.331286\C,0,4.6262
8,1.572946,-1.522009\C,0,4.28369,-0.855123,-1.887093\O,0,5.344868,-1.379203,-1.26
2541\O,0,4.189996,2.681026,-0.88468\O,0,5.550238,1.561661,-2.304407\O,0,3.787599,
-1.307569,-2.897658\C,0,5.887481,-2.598097,-1.821216\C,0,5.105734,-3.806758,-1.33
3339\H,0,6.920774,-2.616947,-1.469217\H,0,5.872559,-2.521381,-2.910652\H,0,5.0979
13,-3.839475,-0.241487\H,0,5.565907,-4.725301,-1.712292\H,0,4.074176,-3.765432,-1.
690338\C,0,4.818055,3.931729,-1.23786\C,0,3.91635,5.038507,-0.72575\H,0,4.946798,
3.966707,-2.322914\H,0,5.813958,3.964586,-0.78344\H,0,2.925008,4.973968,-1.18376\
H,0,4.348249,6.014951,-0.965624\H,0,3.797964,4.97114,0.359296\C,0,-3.014324,-1.24
1489,1.076237\C,0,-4.19802,-1.02475,1.788939\C,0,-1.868809,-1.68466,1.760499\C,0,
-4.232253,-1.216737,3.170839\H,0,-5.088042,-0.6894,1.268482\C,0,-1.913543,-1.8948
82,3.137196\H,0,-0.946566,-1.863474,1.215566\C,0,-3.091573,-1.649057,3.84667\H,0,
-5.150591,-1.023589,3.717212\H,0,-1.023891,-2.248851,3.646469\H,0,-3.119851,-1.79
639,4.922384\C,0,-0.648544,3.548514,-0.47937\C,0,-0.126302,3.51428,-1.778995\C,0,
-0.957782,4.784213,0.105131\C,0,0.108846,4.700708,-2.475639\H,0,0.082591,2.551834,
-2.2372\C,0,-0.727453,5.968748,-0.593438\H,0,-1.373387,4.814483,1.107818\C,0,-0.1
87728,5.928934,-1.881787\H,0,0.513479,4.664549,-3.482787\H,0,-0.967766,6.922979,-
0.133803\H,0,-0.008077,6.852719,-2.423755\C,0,-3.026391,-2.588963,-1.46691\C,0,-2.
831755,-2.711459,-2.851631\C,0,-3.269848,-3.739768,-0.708142\C,0,-2.895957,-3.958
879,-3.467326\H,0,-2.61473,-1.823945,-3.439775\C,0,-3.314413,-4.992445,-1.32463\H,
0,-3.412679,-3.659517,0.363448\C,0,-3.130881,-5.105177,-2.702585\H,0,-2.745408,-4.
040144,-4.539719\H,0,-3.496002,-5.879687,-0.724929\H,0,-3.165414,-6.079996,-3.179
624\C,0,0.563893,1.99832,1.61614\C,0,1.501455,3.03709,1.676909\C,0,0.746279,0.869
171,2.43349\C,0,2.59647,2.949075,2.540865\H,0,1.387668,3.90893,1.043717\C,0,1.832
499,0.789279,3.300148\H,0,0.044709,0.045911,2.371364\C,0,2.765189,1.828785,3.3535
84\H,0,3.322651,3.756167,2.569821\H,0,1.968876,-0.098855,3.908793\H,0,3.628174,1.
752218,4.007001\O,0,1.595802,-0.896878,0.205813\C,0,1.965909,-3.167995,1.54187\C,
0,1.258986,-3.670556,0.438475\C,0,1.595462,-3.606567,2.822224\C,0,0.196807,-4.554

967,0.611141\H,0,1.52129,-3.343112,-0.558551\C,0,0.539735,-4.504088,2.997166\H,0,
2.134389,-3.230105,3.687609\C,0,-0.170031,-4.97417,1.892122\H,0,-0.351904,-4.9064
57,-0.257313\H,0,0.266356,-4.825574,3.998394\H,0,-1.001763,-5.659322,2.027173\\Ve
rsion=ES64L-G16RevA.03\State=1-A\HF=-3581.1895115\RMSD=4.223e-09\Dipole=-2.267330
8,1.723908,1.6765301\Quadrupole=4.3469219,8.9685535,-13.3154754,7.3984172,11.6065
494,-1.1322284\PG=C01 [X(C60H52O6P2Pd1)]\

product-o

\0,1\C,0,-0.371251,3.152529,-1.168909\C,0,1.01316,2.792498,-1.385614\C,0,1.600345,
1.586905,-1.321041\C,0,-1.427771,2.332007,-0.979096\C,0,1.038397,0.224538,-1.0314
12\C,0,-1.496859,0.876544,-0.935207\C,0,-0.454966,0.016051,-1.043571\H,0,-0.58008
8,4.21942,-1.174021\H,0,1.667096,3.625469,-1.63606\H,0,2.662978,1.550157,-1.54725
8\H,0,-2.391582,2.809914,-0.828226\H,0,1.436023,-0.432649,-1.817832\C,0,1.553586,
-0.460447,0.297665\C,0,1.242493,-1.971408,0.154932\C,0,-0.194625,-2.209079,-0.230
338\H,0,1.903537,-2.365221,-0.624879\H,0,1.452754,-2.494431,1.088056\C,0,-0.92284
6,-3.205131,0.206929\C,0,-1.621472,-4.216399,0.644504\H,0,-2.219074,-4.143051,1.5
51494\H,0,-1.642612,-5.171357,0.121595\C,0,3.056955,-0.293553,0.496712\C,0,0.8391
67,0.100446,1.533668\O,0,0.950272,1.430054,1.600003\O,0,3.729466,-0.593632,-0.633
842\O,0,3.591148,0.020507,1.536064\O,0,0.242293,-0.585542,2.336708\C,0,0.35124,2.
067236,2.757466\C,0,-1.13976,2.284306,2.569305\H,0,0.889053,3.013134,2.84363\H,0,
0.558141,1.449509,3.634057\H,0,-1.331764,2.878643,1.674016\H,0,-1.543905,2.814432,
3.437969\H,0,-1.661976,1.330355,2.474443\C,0,5.173683,-0.516124,-0.547831\C,0,5.7
23507,-0.859846,-1.916153\H,0,5.514091,-1.211645,0.224684\H,0,5.44921,0.493229,-0.
229263\H,0,5.422767,-1.867573,-2.215481\H,0,6.816454,-0.817049,-1.897934\H,0,5.36
3493,-0.154124,-2.669917\O,0,-0.76918,-1.329189,-1.153297\C,0,-2.869904,0.320916,
-0.734426\C,0,-3.918013,0.680513,-1.590652\C,0,-3.145247,-0.51736,0.35634\C,0,-5.
210141,0.202495,-1.373731\H,0,-3.713999,1.328139,-2.43843\C,0,-4.436217,-0.991969,
0.574019\H,0,-2.342969,-0.790463,1.03317\C,0,-5.472979,-0.635732,-0.290403\H,0,-6.
009931,0.48334,-2.052543\H,0,-4.63354,-1.638934,1.42355\H,0,-6.479026,-1.006451,-
0.118858\\Version=ES64L-G16RevA.03\State=1-A\HF=-1305.6710014\RMSD=6.715e-09\Dipo
le=0.9560363,0.4031874,-0.6895158\Quadrupole=8.5315903,3.117698,-11.6492883,-2.08
66789,-10.3265178,0.2061321\PG=C01 [X(C24H24O5)]\

2a-Yb

\0,1\C,0,-2.236298,-0.220408,1.980542\C,0,-3.36026,-0.263834,3.049319\C,0,-3.4266
09,-1.172199,1.927468\H,0,-4.017197,0.600559,3.063918\H,0,-3.058211,-0.655711,4.0
16324\C,0,-3.912477,-2.111862,1.193426\C,0,-4.393202,-3.065042,0.441206\H,0,-5.07
6908,-2.849636,-0.374183\H,0,-4.104196,-4.101826,0.591937\C,0,-2.252815,0.861074,
0.951753\C,0,-0.914466,-0.824681,2.318498\O,0,-0.978332,-1.74038,3.259928\O,0,-3.
343552,1.597303,0.979189\O,0,-1.367576,1.041039,0.115031\O,0,0.151127,-0.509786,1.
785007\C,0,0.26452,-2.46657,3.552258\C,0,-0.104654,-3.623044,4.453405\H,0,0.95363,
-1.762721,4.025526\H,0,0.695301,-2.784935,2.600776\H,0,-0.552794,-3.271376,5.3866
78\H,0,0.798342,-4.189904,4.697116\H,0,-0.808302,-4.295152,3.955607\C,0,-3.491854,
2.60197,-0.080321\C,0,-4.82367,3.281322,0.145049\H,0,-3.438604,2.079394,-1.037008

\H,0,-2.647296,3.288042,-0.009977\H,0,-5.645854,2.562886,0.089912\H,0,-4.976073,4.
038961,-0.628832\H,0,-4.853081,3.776002,1.119592\Yb,0,0.674885,0.022409,-0.412357
\O,0,-1.0444,-1.790313,-0.468767\O,0,1.717527,1.769336,0.796652\O,0,2.806378,-0.1
27453,-1.430007\S,0,3.193197,-1.464584,-0.844122\S,0,-1.103379,-1.823328,-1.97156
1\S,0,1.57582,2.835381,-0.268718\O,0,2.746315,3.622506,-0.607981\O,0,0.812643,2.1
75013,-1.391015\O,0,1.981782,-1.884155,-0.027324\O,0,3.790892,-2.454931,-1.718954
\O,0,-0.224811,-0.677067,-2.432318\O,0,-0.938533,-3.102446,-2.637419\C,0,-2.82680
7,-1.253937,-2.426602\C,0,0.354786,4.046358,0.478511\C,0,4.455973,-1.046859,0.456
543\F,0,-3.293608,-0.363457,-1.30635\F,0,-2.807446,-0.684823,-3.627157\F,0,-3.652
96,-2.302594,-2.444388\F,0,5.508322,-0.463756,-0.109494\F,0,3.912282,-0.216061,1.
353105\F,0,4.838514,-2.166256,1.0747\F,0,-0.478422,3.425574,1.332095\F,0,1.012465,
4.991918,1.142762\F,0,-0.375767,4.605483,-0.491483\\Version=ES64L-G16RevC.01\Stat
e=1-A\HF=-3653.8721993\RMSD=5.396e-09\Dipole=-5.2090864,0.0903226,4.4852899\Quadr
upole=4.3392557,-7.7513937,3.412138,-8.9200825,3.7545734,-13.8065662\PG=C01 [X(C1
4H14F9O13S3Yb1)]\

int-1-Yb

\0,1\O,0,-5.604394,-0.86253,0.715823\C,0,-5.814502,-2.108875,0.172812\C,0,-4.8824
25,-0.656018,1.869304\C,0,-4.837339,-2.675657,-0.660732\C,0,-7.057559,-2.703423,0.
370955\C,0,-7.357675,-3.902192,-0.273249\C,0,-5.173871,-3.871971,-1.312965\C,0,-6.
414469,-4.481511,-1.120667\H,0,-4.461281,-4.333061,-1.984555\H,0,-6.641526,-5.406
93,-1.640359\H,0,-8.325272,-4.370452,-0.123364\H,0,-7.774899,-2.202427,1.011817\C,
0,-4.277696,0.607914,1.941515\C,0,-4.767341,-1.591774,2.889371\C,0,-4.02549,-1.25
4221,4.023107\C,0,-3.569021,0.935064,3.101619\C,0,-3.440514,0.007128,4.136679\H,0,
-3.087732,1.902647,3.180906\H,0,-2.867793,0.265069,5.021117\H,0,-5.228281,-2.5673
63,2.785278\H,0,-3.905693,-1.983953,4.817228\P,0,-3.173248,-1.86599,-0.819324\P,0,
-4.179111,1.567023,0.379989\C,0,-2.32321,-2.513298,0.672453\C,0,-2.493197,-2.8138
43,-2.240995\C,0,-5.886659,1.766098,-0.239329\C,0,-3.744342,3.257361,0.952044\C,0,
-6.997304,1.832725,0.611973\C,0,-6.07023,1.856837,-1.623966\C,0,-7.351322,2.02398,
-2.152179\C,0,-8.275619,1.995377,0.082131\C,0,-8.454178,2.092968,-1.30055\H,0,-6.
858079,1.744019,1.685253\H,0,-5.207854,1.783758,-2.281111\H,0,-7.487353,2.093029,
-3.22725\H,0,-9.133597,2.042758,0.746221\H,0,-9.451587,2.217165,-1.711337\C,0,-2.
615994,-3.764199,1.235806\C,0,-1.352429,-1.700405,1.275564\C,0,-0.666771,-2.13755
9,2.408228\C,0,-0.951202,-3.391084,2.950569\C,0,-1.927814,-4.201542,2.366561\H,0,
-1.141506,-0.721164,0.856413\H,0,0.098191,-1.509991,2.848737\H,0,-0.412761,-3.734
188,3.828516\H,0,-2.156683,-5.173236,2.794311\H,0,-3.392894,-4.385947,0.802326\C,
0,-2.029854,-4.132228,-2.145282\C,0,-2.495384,-2.18174,-3.491389\C,0,-2.041984,-2.
854346,-4.625858\C,0,-1.577384,-4.806115,-3.280617\C,0,-1.580277,-4.168007,-4.522
671\H,0,-2.018584,-4.633573,-1.183999\H,0,-2.833821,-1.152394,-3.562973\H,0,-2.04
5623,-2.352144,-5.588683\H,0,-1.219791,-5.827926,-3.193276\H,0,-1.223787,-4.69161
5,-5.40441\C,0,-2.399445,3.540464,1.227403\C,0,-4.701965,4.265381,1.131251\C,0,-4.
317145,5.527076,1.585872\C,0,-2.019011,4.795586,1.698938\C,0,-2.97839,5.79349,1.8
7603\H,0,-1.653856,2.779847,1.044688\H,0,-5.745063,4.070781,0.911864\H,0,-5.06715,
6.301673,1.715112\H,0,-0.974818,4.991162,1.916933\H,0,-2.68334,6.775411,2.233451\

Pd,0,-2.646289,0.481895,-1.036387\C,0,0.626057,0.447106,-2.015894\C,0,-0.133569,0.
423288,-3.362962\C,0,-0.911437,0.792133,-2.186586\H,0,-0.23976,-0.575145,-3.77568
5\H,0,0.150723,1.201411,-4.067025\C,0,-1.547658,1.979714,-1.793967\C,0,-1.602475,
3.295977,-1.96461\H,0,-0.861868,3.811638,-2.573349\H,0,-2.374849,3.902557,-1.5044
87\C,0,1.099408,-0.851467,-1.47147\C,0,1.483025,1.610262,-1.684724\O,0,1.330526,2.
641255,-2.494295\O,0,0.513876,-1.921769,-1.961807\O,0,2.040115,-0.967435,-0.67453
8\O,0,2.259581,1.66229,-0.721225\C,0,2.073688,3.863436,-2.193774\C,0,2.299148,4.5
81049,-3.506407\H,0,3.006953,3.595862,-1.701634\H,0,1.459782,4.43991,-1.496692\H,
0,2.914023,3.969893,-4.170844\H,0,2.825287,5.521512,-3.319067\H,0,1.353502,4.8072
75,-4.00683\C,0,1.192686,-3.208069,-1.715763\C,0,0.979826,-3.761043,-0.321557\H,0,
2.254161,-3.051144,-1.918335\H,0,0.754917,-3.845346,-2.481284\H,0,1.472821,-3.148
093,0.431592\H,0,1.42303,-4.762097,-0.286851\H,0,-0.07981,-3.846217,-0.08292\Yb,0,
3.597991,0.189635,0.504848\O,0,4.06084,-2.110514,0.935686\O,0,5.15837,1.814305,-0.
313723\O,0,3.066906,1.904147,2.079046\S,0,2.147976,1.085119,2.945132\S,0,5.109654,
-1.869161,1.996851\S,0,5.730414,0.969792,-1.423063\O,0,7.174407,0.857267,-1.52095
2\O,0,4.916063,-0.304168,-1.415079\O,0,2.109337,-0.288091,2.317117\O,0,2.282671,1.
179153,4.386468\O,0,5.281904,-0.362594,2.038253\O,0,4.963003,-2.566834,3.262911\C,
0,6.696369,-2.482654,1.242828\C,0,5.194922,1.822491,-2.998322\C,0,0.455475,1.7410
37,2.545841\F,0,6.952199,-1.833911,0.107984\F,0,7.695088,-2.283975,2.102814\F,0,6.
578383,-3.788929,0.989325\F,0,0.410353,3.059241,2.74302\F,0,0.158473,1.483756,1.2
56431\F,0,-0.459813,1.143226,3.313261\F,0,3.899834,1.565469,-3.26208\F,0,5.343152,
3.145311,-2.873208\F,0,5.927411,1.386234,-4.018369\\Version=ES64L-G16RevC.01\Stat
e=1-A\HF=-5929.3669921\RMSD=2.648e-09\Dipole=-7.6598244,-1.0107732,-3.7834004\Qua
drupole=-30.1708092,35.4946507,-5.3238415,13.9100914,-23.2169712,3.1585783\PG=C01
[X(C50H42F9O14P2Pd1S3Yb1)]\

TS-1-Yb

\0,1\O,0,3.884233,-0.668489,1.958108\C,0,4.890777,0.279809,1.877916\C,0,4.190072,
-2.002555,1.769519\C,0,4.516083,1.513039,1.323992\C,0,6.176008,0.04555,2.353773\C,
0,7.124275,1.065255,2.266706\C,0,5.480683,2.522762,1.259115\C,0,6.779941,2.300309,
1.719779\H,0,5.214403,3.488131,0.84484\H,0,7.517057,3.094131,1.655505\H,0,8.13184
4,0.890155,2.630971\H,0,6.428571,-0.920646,2.775602\C,0,4.239913,-2.52134,0.46333
7\C,0,4.351792,-2.814586,2.887789\C,0,4.59744,-4.177272,2.717992\C,0,4.495101,-3.
893366,0.318554\C,0,4.677738,-4.713108,1.432544\H,0,4.536921,-4.323747,-0.675132\
H,0,4.873094,-5.771626,1.293616\H,0,4.272498,-2.366546,3.872364\H,0,4.722789,-4.8
14841,3.587299\P,0,2.769952,1.653801,0.761482\P,0,3.794042,-1.443983,-0.964024\C,
0,1.913188,1.65249,2.375322\C,0,2.615382,3.374123,0.155187\C,0,5.30338,-0.463334,
-1.31887\C,0,3.785787,-2.64753,-2.352036\C,0,6.566488,-0.786909,-0.814142\C,0,5.1
6431,0.651602,-2.160395\C,0,6.271968,1.431765,-2.484489\C,0,7.672354,0.003383,-1.
130332\C,0,7.527946,1.112223,-1.963694\H,0,6.682874,-1.646688,-0.163278\H,0,4.181
285,0.907964,-2.545971\H,0,6.153405,2.29305,-3.135341\H,0,8.645639,-0.245592,-0.7
18926\H,0,8.389644,1.727043,-2.204845\C,0,2.393822,2.470219,3.410458\C,0,0.807885,
0.827644,2.598568\C,0,0.187769,0.817472,3.849612\C,0,0.664192,1.635383,4.87173\C,
0,1.766051,2.466114,4.651998\H,0,0.417127,0.216192,1.79459\H,0,-0.678527,0.189988,

4.009165\H,0,0.170955,1.630748,5.838979\H,0,2.136453,3.106726,5.4469\H,0,3.257899,3.10568,3.242264\C,0,1.604954,4.21466,0.64046\C,0,3.399289,3.796927,-0.931276\C,0,3.195198,5.05105,-1.501569\C,0,1.393789,5.464448,0.052885\C,0,2.188357,5.888267,-1.011842\H,0,0.965589,3.890275,1.453072\H,0,4.161301,3.13836,-1.335667\H,0,3.813253,5.371427,-2.33524\H,0,0.596346,6.098345,0.427468\H,0,2.021766,6.861148,-1.464282\C,0,2.607064,-3.369561,-2.587116\C,0,4.905863,-2.883681,-3.160136\C,0,4.843345,-3.826838,-4.187452\C,0,2.551191,-4.318688,-3.606626\C,0,3.669349,-4.547366,-4.411492\H,0,1.732863,-3.174251,-1.97939\H,0,5.824281,-2.333641,-2.988346\H,0,5.71525,-3.999425,-4.811284\H,0,1.632228,-4.871981,-3.776849\H,0,3.624885,-5.280524,-5.211087\Pd,0,2.007658,0.152723,-0.926114\C,0,-1.095801,-0.934949,-2.305235\C,0,0.022597,-1.028309,-3.334975\C,0,0.704931,-0.086932,-2.456466\H,0,-0.279269,-0.641182,-4.307561\H,0,0.47114,-2.01512,-3.405025\C,0,0.739178,1.229907,-2.101491\C,0,0.412583,2.485079,-2.417586\H,0,0.697473,3.3271,-1.800055\H,0,-0.157971,2.691187,-3.322009\C,0,-2.134437,0.052533,-2.48472\C,0,-1.135398,-1.79942,-1.155739\O,0,-0.218722,-2.770247,-1.13601\O,0,-2.020562,0.750748,-3.617665\O,0,-3.076471,0.273078,-1.696815\O,0,-1.951517,-1.709178,-0.2161\C,0,-0.117044,-3.572012,0.08145\C,0,0.543825,-2.802102,1.211278\H,0,0.489509,-4.421482,-0.23804\H,0,-1.114677,-3.916041,0.356566\H,0,1.476314,-2.345677,0.873173\H,0,0.776371,-3.491399,2.028877\H,0,-0.116026,-2.02647,1.598982\C,0,-3.060328,1.708797,-3.965587\C,0,-3.833239,1.183578,-5.160459\H,0,-2.527957,2.634396,-4.196217\H,0,-3.70176,1.86538,-3.102351\H,0,-3.169961,1.000477,-6.010405\H,0,-4.58615,1.919185,-5.460172\H,0,-4.346246,0.255033,-4.904202\Yb,0,-3.397701,-0.138728,0.539586\O,0,-1.461284,1.381159,0.342159\O,0,-4.919599,-1.742233,-0.396146\O,0,-4.448478,-1.019042,2.520946\S,0,-3.183177,-1.307309,3.281682\S,0,-2.092516,2.505511,1.115173\S,0,-6.062043,-0.777864,-0.590758\O,0,-7.392787,-1.202091,-0.193608\O,0,-5.562551,0.548061,-0.060851\O,0,-2.064978,-0.791063,2.405914\O,0,-3.122155,-0.977644,4.695848\O,0,-3.437349,1.988823,1.56719\O,0,-1.27575,3.222112,2.08306\C,0,-2.546569,3.773625,-0.168312\C,0,-6.145742,-0.522783,-2.463066\C,0,-2.988061,-3.154305,3.158458\F,0,-3.31488,3.219873,-1.11647\F,0,-3.21281,4.773401,0.404736\F,0,-1.438102,4.259011,-0.743949\F,0,-3.887585,-3.764248,3.925057\F,0,-3.152833,-3.544245,1.886633\F,0,-1.756005,-3.497692,3.555212\F,0,-5.154426,-1.185732,-3.074985\F,0,-7.30904,-0.97822,-2.922527\F,0,-6.039815,0.777787,-2.757695\\Version=ES64L-G16RevA.03\State=1-A\HF=-5929.3652203\RMSD=1.558e-09\Dipole=8.6497916,-0.8403021,-4.1627107\Quadrupole=-19.8987299,19.4857987,0.4129312,-13.1443509,22.5772753,-9.5869888\PG=C01 [X(C50H42F9O14P2Pd1S3Yb1)]\

int-2a-Yb

\0,1\O,0,3.033663,0.246947,2.314495\C,0,4.321637,0.685673,2.114182\C,0,2.792671,-1.043711,2.739895\C,0,4.431121,1.666067,1.11945\C,0,5.428056,0.226607,2.81887\C,0,6.674814,0.788188,2.536686\C,0,5.682431,2.235598,0.872773\C,0,6.802571,1.797795,1.58168\H,0,5.785153,3.000566,0.111618\H,0,7.774414,2.23608,1.379643\H,0,7.549614,0.433557,3.072436\H,0,5.315092,-0.556334,3.559583\C,0,2.711401,-2.072162,1.792601\C,0,2.56129,-1.263283,4.091458\C,0,2.269628,-2.550606,4.535932\C,0,2.42151,-3.36222,2.263348\C,0,2.211427,-3.59973,3.620957\H,0,2.347323,-4.18501,1.564831\H,0,1.979368,-4.605385,3.955632\H,0,2.586234,-0.416186,4.767025\H,0,2.076868,-2.727577,5.58

8805\P,0,2.901129,1.944065,0.160981\P,0,3.051532,-1.769232,0.005402\C,0,1.774941,
2.785999,1.314163\C,0,3.425868,3.209414,-1.060931\C,0,4.881005,-1.767777,-0.04912
3\C,0,2.542652,-3.365732,-0.729523\C,0,5.624945,-2.63543,0.764063\C,0,5.546959,-0.
88967,-0.909308\C,0,6.939887,-0.86926,-0.953433\C,0,7.016794,-2.619799,0.711913\C,
0,7.675974,-1.733916,-0.143461\H,0,5.115288,-3.310265,1.44366\H,0,4.96275,-0.2307
05,-1.539746\H,0,7.448349,-0.173796,-1.613858\H,0,7.587161,-3.291993,1.345613\H,0,
8.761127,-1.715286,-0.174067\C,0,2.231945,3.810488,2.157861\C,0,0.446067,2.354984,
1.394043\C,0,-0.426563,2.946711,2.307597\C,0,0.025238,3.978661,3.128995\C,0,1.352
969,4.410886,3.055887\H,0,0.102234,1.544153,0.763586\H,0,-1.449972,2.599727,2.372
897\H,0,-0.657,4.441346,3.835406\H,0,1.704651,5.206879,3.70554\H,0,3.270606,4.125
444,2.11624\C,0,2.955491,4.52821,-1.040279\C,0,4.302215,2.81497,-2.08655\C,0,4.71
6049,3.725636,-3.056189\C,0,3.358233,5.434601,-2.023003\C,0,4.240706,5.038808,-3.
028225\H,0,2.272874,4.844848,-0.259646\H,0,4.656429,1.78996,-2.126471\H,0,5.39692
6,3.407574,-3.839822\H,0,2.98198,6.452757,-1.99878\H,0,4.553181,5.747119,-3.78915
1\C,0,1.175641,-3.684954,-0.705856\C,0,3.455861,-4.249284,-1.312242\C,0,3.002111,
-5.44456,-1.875608\C,0,0.735613,-4.887115,-1.249767\C,0,1.645948,-5.767487,-1.841
762\H,0,0.468336,-2.990923,-0.267\H,0,4.512371,-4.007951,-1.331643\H,0,3.713184,-
6.124396,-2.335255\H,0,-0.320968,-5.12745,-1.215619\H,0,1.298289,-6.700689,-2.274
351\Pd,0,2.131182,0.031703,-1.097194\C,0,-0.741874,-0.908219,-2.253446\C,0,0.3635
31,-1.511571,-3.090719\C,0,1.789062,-1.203946,-2.675197\H,0,0.261519,-1.196987,-4.
13843\H,0,0.238565,-2.595194,-3.081296\C,0,2.862763,-1.441833,-3.360706\C,0,3.982
183,-1.742659,-3.987086\H,0,4.494331,-1.033461,-4.63372\H,0,4.431951,-2.728617,-3.
881172\C,0,-0.733768,0.430277,-1.867608\C,0,-1.853666,-1.701546,-1.859608\O,0,-1.
871316,-2.95432,-2.339374\O,0,0.409497,1.129231,-2.230149\O,0,-1.619695,1.08758,-
1.244803\O,0,-2.813293,-1.323756,-1.132476\C,0,-3.04041,-3.760848,-2.046606\C,0,-
2.95354,-4.995116,-2.921056\H,0,-3.937411,-3.174669,-2.254259\H,0,-3.040646,-4.00
3551,-0.982399\H,0,-2.953572,-4.721699,-3.979719\H,0,-3.815491,-5.641402,-2.73062
2\H,0,-2.043182,-5.565601,-2.715708\C,0,0.280425,2.572605,-2.413272\C,0,1.057955,
2.947302,-3.655336\H,0,0.662382,3.061165,-1.517124\H,0,-0.779783,2.803463,-2.4950
67\H,0,2.10019,2.634182,-3.576631\H,0,1.039515,4.033566,-3.78249\H,0,0.616602,2.4
81759,-4.540905\Yb,0,-3.455365,0.543321,-0.081867\O,0,-5.406735,-0.243768,-1.2127
99\O,0,-0.018527,-1.004254,0.823336\O,0,-3.455041,2.486835,1.318838\S,0,-4.087235,
3.432482,0.326186\S,0,-6.195825,-0.605265,0.021811\S,0,-0.957088,-0.941834,1.9599
49\O,0,-0.424823,-0.605993,3.276848\O,0,-2.217582,-0.170602,1.618469\O,0,-4.25357
4,2.6212,-0.938374\O,0,-5.213664,4.234171,0.770066\O,0,-5.342301,-0.155821,1.1860
3\O,0,-7.611401,-0.284942,0.043376\C,0,-6.075662,-2.46664,0.101508\C,0,-1.611193,
-2.684453,2.111261\C,0,-2.731689,4.648799,-0.057886\F,0,-4.841215,-2.832191,0.475
146\F,0,-6.952727,-2.94041,0.98376\F,0,-6.331371,-2.989644,-1.103592\F,0,-2.41161
8,5.31545,1.052873\F,0,-1.641285,4.013662,-0.509436\F,0,-3.147531,5.506843,-0.990
086\F,0,-1.7494,-3.239443,0.890073\F,0,-0.7455,-3.431305,2.806388\F,0,-2.793821,-
2.701522,2.723239\\Version=ES64L-G16RevA.03\State=1-A\HF=-5929.3937069\RMSD=1.432
e-09\Dipole=9.8051241,-1.4980036,-1.796125\Quadrupole=-22.3652934,24.0834729,-1.7
181795,23.9414187,9.0950034,-8.175872\PG=C01 [X(C50H42F9O14P2Pd1S3Yb1)]\

int-3-Yb

\0,1\C,0,1.369018,2.558684,-4.662225\C,0,1.970721,3.784805,-4.517751\C,0,2.640648,
4.283135,-3.373765\C,0,1.286601,1.523646,-3.702707\C,0,2.933228,3.689138,-2.17292
\C,0,1.830923,1.43183,-2.447413\C,0,2.660612,2.354149,-1.719806\H,0,0.879414,2.36
0084,-5.611789\H,0,1.909391,4.466094,-5.362161\H,0,2.982195,5.311869,-3.459873\H,
0,0.708791,0.655821,-3.996204\H,0,3.466999,4.316328,-1.473958\H,0,1.632422,0.5068
47,-1.918272\N,0,3.133093,1.870904,-0.561736\S,0,4.43022,2.574441,0.27227\O,0,4.9
70797,1.497369,1.119428\O,0,5.352802,3.229124,-0.668544\C,0,4.074457,5.396538,3.1
07368\C,0,4.572411,4.403694,2.270085\C,0,3.719456,3.825422,1.325866\C,0,2.388921,
4.217933,1.207696\C,0,1.913582,5.215173,2.058114\C,0,2.738476,5.817272,3.014666\H,
0,4.727271,5.845844,3.850464\H,0,5.60213,4.073349,2.354576\H,0,1.704792,3.757246,
0.503665\H,0,0.870934,5.50822,1.978765\C,0,2.208277,6.904113,3.914981\H,0,2.76973
9,6.957742,4.851869\H,0,1.154127,6.74024,4.155084\H,0,2.284223,7.884965,3.429768\
O,0,1.579757,-2.831257,-1.083251\C,0,2.234921,-3.779721,-0.303258\C,0,2.072891,-2.
682712,-2.369134\C,0,2.522978,-3.434079,1.021366\C,0,2.562678,-5.029894,-0.823839
\C,0,3.220997,-5.952641,-0.015319\C,0,3.200228,-4.373186,1.809924\C,0,3.552585,-5.
619668,1.297536\H,0,3.469562,-4.117899,2.826676\H,0,4.088486,-6.325645,1.923063\H,
0,3.482843,-6.926243,-0.417471\H,0,2.314393,-5.26517,-1.852126\C,0,3.300981,-2.02
962,-2.585657\C,0,1.326145,-3.210498,-3.418265\C,0,1.812944,-3.12749,-4.72249\C,0,
3.791543,-2.007529,-3.901111\C,0,3.058902,-2.546538,-4.958555\H,0,4.749655,-1.544
338,-4.100781\H,0,3.462537,-2.507588,-5.965204\H,0,0.384983,-3.697842,-3.189274\H,
0,1.231744,-3.537237,-5.542364\P,0,1.991148,-1.789005,1.649321\P,0,4.124963,-1.13
6613,-1.196598\C,0,0.246526,-2.111111,2.110038\C,0,2.836435,-1.641092,3.263645\C,
0,5.224686,-2.357127,-0.390308\C,0,5.324293,-0.021108,-2.038963\C,0,5.597894,-3.5
62733,-0.991841\C,0,5.728883,-2.02983,0.876563\C,0,6.590397,-2.902787,1.536727\C,
0,6.455651,-4.437284,-0.324976\C,0,6.9521,-4.110756,0.937722\H,0,5.207966,-3.8242
52,-1.969494\H,0,5.450895,-1.088914,1.333938\H,0,6.967032,-2.64098,2.520664\H,0,6.
731,-5.378377,-0.791298\H,0,7.615883,-4.797504,1.454212\C,0,-0.507369,-3.103448,1.
473704\C,0,-0.342545,-1.345603,3.129713\C,0,-1.66294,-1.574905,3.5041\C,0,-2.4109
48,-2.571307,2.867712\C,0,-1.830282,-3.334793,1.8529\H,0,0.227064,-0.563359,3.616
72\H,0,-2.114363,-0.96176,4.275082\H,0,-3.44207,-2.743609,3.152935\H,0,-2.406326,
-4.107208,1.354048\H,0,-0.078072,-3.698425,0.67886\C,0,2.459475,-2.440379,4.35505
6\C,0,3.886771,-0.726567,3.410613\C,0,4.579038,-0.644749,4.619337\C,0,3.150144,-2.
351166,5.561898\C,0,4.217627,-1.458574,5.692411\H,0,1.624342,-3.126429,4.260534\H,
0,4.137861,-0.052466,2.600364\H,0,5.384988,0.075095,4.723034\H,0,2.853037,-2.9742
74,6.399858\H,0,4.75342,-1.388498,6.634421\C,0,4.936139,0.764711,-3.135416\C,0,6.
615759,0.143425,-1.520285\C,0,7.491845,1.068651,-2.084963\C,0,5.814078,1.687332,-
3.697197\C,0,7.095977,1.844761,-3.171824\H,0,3.947105,0.656711,-3.558711\H,0,6.94
0415,-0.441213,-0.669459\H,0,8.485117,1.186481,-1.663374\H,0,5.48759,2.288711,-4.
54023\H,0,7.778084,2.57113,-3.602378\Pd,0,2.559821,-0.02067,0.313469\C,0,-0.48212
7,1.234401,-0.430102\C,0,-0.196062,1.250183,1.051902\C,0,1.243213,1.034407,1.5110
09\H,0,-0.792692,0.46295,1.511192\H,0,-0.53913,2.194287,1.482656\C,0,1.704316,1.4
98075,2.631748\C,0,2.175134,1.96138,3.768785\H,0,2.700593,2.910823,3.823073\H,0,2.
063427,1.403372,4.696091\C,0,-0.472763,2.505688,-1.104891\C,0,-1.054926,0.081552,

-0.986835\O,0,-0.864308,-0.135326,-2.29846\O,0,-1.160984,2.540981,-2.287535\O,0,0.
023836,3.540988,-0.646692\O,0,-1.713423,-0.800568,-0.308166\C,0,-1.686622,-1.0683
19,-3.035852\C,0,-2.210923,-0.366628,-4.274525\H,0,-2.49594,-1.436115,-2.413153\H,
0,-1.040593,-1.911768,-3.29471\H,0,-2.820709,0.488969,-3.984074\H,0,-2.829276,-1.
059337,-4.854445\H,0,-1.393637,-0.018485,-4.91528\C,0,-1.381574,3.84322,-2.840293
\C,0,-2.126596,3.647847,-4.146949\H,0,-0.42443,4.35299,-2.986654\H,0,-1.966262,4.
440734,-2.132195\H,0,-1.539091,3.03912,-4.840625\H,0,-2.325962,4.615816,-4.617991
\H,0,-3.079923,3.144503,-3.97327\Yb,0,-3.792639,-0.603731,0.337485\O,0,-5.817213,
0.373334,-0.315605\O,0,-4.399766,-0.129221,2.593097\O,0,-4.870022,-2.593827,1.022
368\S,0,-4.864421,-3.312429,-0.310118\S,0,-5.35046,0.950439,-1.636009\S,0,-3.7596
64,1.239556,2.666511\O,0,-2.878423,1.504808,3.794329\O,0,-3.21009,1.481973,1.2815
3\O,0,-4.263286,-2.325572,-1.280975\O,0,-4.340815,-4.671476,-0.323248\O,0,-3.8752
08,0.642838,-1.689272\O,0,-6.14735,0.658796,-2.815577\C,0,-5.432132,2.799412,-1.4
08288\C,0,-5.175814,2.437702,2.829369\C,0,-6.657664,-3.425884,-0.793735\F,0,-4.55
8996,3.207542,-0.490506\F,0,-5.142576,3.386273,-2.576154\F,0,-6.665002,3.141804,-
1.035248\F,0,-7.289047,-4.225798,0.066324\F,0,-7.212654,-2.217554,-0.770542\F,0,-
6.74048,-3.93471,-2.02346\F,0,-6.07552,2.232234,1.868683\F,0,-5.75211,2.269388,4.
019888\F,0,-4.699927,3.67986,2.732856\\Version=ES64L-G16RevA.03\State=1-A\HF=-707
4.4437068\RMSD=2.575e-09\Dipole=6.7694684,-1.0365549,-2.8550901\Quadrupole=-44.79
92029,16.0117385,28.7874644,-21.4333238,-3.7123448,-8.8190102\PG=C01 [X(C64H55F9N
1O16P2Pd1S4Yb1)]\

TS-2-Yb

\0,1\C,0,1.406028,2.651845,2.530642\C,0,2.007514,3.040438,1.298274\C,0,1.585359,2.
762018,0.02935\C,0,0.150454,2.127849,2.702851\C,0,0.485757,1.910297,-0.393118\C,0,
-0.861517,1.899966,1.73357\C,0,-0.782286,1.910206,0.35353\H,0,1.999631,2.822382,3.
424251\H,0,2.939183,3.593481,1.375958\H,0,2.212128,3.138048,-0.773281\H,0,-0.1557
91,1.937066,3.729485\H,0,0.306407,1.948534,-1.459363\H,0,-1.848274,1.698922,2.136
71\N,0,-2.000303,1.793679,-0.332244\S,0,-2.331816,2.561133,-1.75568\O,0,-1.198338,
2.637607,-2.704527\O,0,-3.600466,1.975881,-2.23154\O,0,-3.30029,-2.022985,2.24236
3\C,0,-4.326998,-2.878717,1.855839\C,0,-3.614513,-1.047047,3.176468\C,0,-4.440708,
-3.174806,0.490979\C,0,-5.187673,-3.429589,2.802047\C,0,-6.211043,-4.276208,2.383
637\C,0,-5.495083,-4.008174,0.094234\C,0,-6.373685,-4.554464,1.027562\H,0,-5.6454
19,-4.212038,-0.957721\H,0,-7.186278,-5.190068,0.69183\H,0,-6.886418,-4.704673,3.
117475\H,0,-5.062719,-3.17978,3.84921\C,0,-4.29002,0.121728,2.779703\C,0,-3.21016
9,-1.250915,4.49271\C,0,-3.49746,-0.289639,5.461555\C,0,-4.588127,1.061312,3.7797
17\C,0,-4.199844,0.860682,5.104486\H,0,-5.113529,1.969322,3.511464\H,0,-4.44218,1.
608156,5.85297\H,0,-2.677165,-2.163056,4.736889\H,0,-3.18115,-0.446093,6.487868\P,
0,-3.240992,-2.429582,-0.693752\P,0,-4.613666,0.449754,0.990829\C,0,-1.779701,-3.
533962,-0.580372\C,0,-3.948097,-2.736003,-2.34723\C,0,-6.168021,-0.427243,0.59187
5\C,0,-5.158438,2.206502,1.017889\C,0,-7.045273,-0.931324,1.556579\C,0,-6.485099,
-0.558018,-0.767842\C,0,-7.656553,-1.201997,-1.158272\C,0,-8.214655,-1.58168,1.16
271\C,0,-8.521103,-1.719962,-0.191852\H,0,-6.808472,-0.828486,2.609913\H,0,-5.812
073,-0.151768,-1.513377\H,0,-7.884661,-1.307026,-2.214404\H,0,-8.883869,-1.986011,

1.915925\H,0,-9.430011,-2.2319,-0.493044\C,0,-1.224366,-3.833729,0.67372\C,0,-1.0
84809,-3.909143,-1.739454\C,0,0.150519,-4.548539,-1.643876\C,0,0.717767,-4.808918,
-0.395519\C,0,0.016417,-4.464295,0.761005\H,0,-1.485369,-3.663134,-2.715208\H,0,0.
689741,-4.80884,-2.548226\H,0,1.704461,-5.25544,-0.325868\H,0,0.448286,-4.665801,
1.736529\H,0,-1.732236,-3.53084,1.580524\C,0,-4.124627,-4.033923,-2.854798\C,0,-4.
308731,-1.634645,-3.135554\C,0,-4.875941,-1.830638,-4.395258\C,0,-4.689873,-4.225
084,-4.113904\C,0,-5.074299,-3.122579,-4.881994\H,0,-3.807032,-4.891855,-2.270847
\H,0,-4.11277,-0.628235,-2.780811\H,0,-5.142538,-0.969228,-4.999462\H,0,-4.824138,
-5.231718,-4.497679\H,0,-5.511953,-3.272958,-5.864327\C,0,-4.254848,3.212832,1.39
62\C,0,-6.480637,2.563889,0.720394\C,0,-6.89133,3.894933,0.814029\C,0,-4.674582,4.
533945,1.512697\C,0,-5.993891,4.880681,1.220288\H,0,-3.221398,2.969146,1.590639\H,
0,-7.196223,1.807089,0.424138\H,0,-7.918797,4.154299,0.577016\H,0,-3.956702,5.297
291,1.791958\H,0,-6.314381,5.915213,1.2958\Pd,0,-2.764387,-0.203757,-0.409124\C,0,
1.173491,-0.022225,-0.498719\C,0,0.105223,-1.056907,-0.884805\C,0,-1.142745,-0.62
4199,-1.615917\H,0,-0.190599,-1.557798,0.03075\H,0,0.621883,-1.812941,-1.48197\C,
0,-1.278539,-0.494777,-2.897058\C,0,-1.449809,-0.307065,-4.186431\H,0,-1.3178,0.6
83576,-4.613754\H,0,-1.755454,-1.113897,-4.848094\C,0,2.154586,0.240007,-1.575073
\C,0,1.729558,-0.20733,0.840977\O,0,0.90889,-0.774008,1.713127\O,0,1.562334,0.281
577,-2.753217\O,0,3.373461,0.457316,-1.448403\O,0,2.860643,0.175727,1.205508\C,0,
1.43406,-0.999405,3.049157\C,0,0.30566,-1.601445,3.855474\H,0,1.766409,-0.040352,
3.451162\H,0,2.302011,-1.656278,2.965669\H,0,-0.538668,-0.910201,3.91491\H,0,0.65
3748,-1.81934,4.869556\H,0,-0.044049,-2.531255,3.398309\C,0,2.278229,0.745875,-3.
929601\C,0,2.05407,2.238737,-4.090193\H,0,3.331191,0.488971,-3.833953\H,0,0.98717,
2.472465,-4.054879\H,0,2.460812,2.568193,-5.051626\H,0,2.567716,2.795062,-3.30326
4\Yb,0,4.825597,-0.283786,0.219315\O,0,3.28743,-2.171456,-0.367107\O,0,5.016759,2.
110214,0.323443\O,0,6.400652,-0.018331,1.967886\S,0,5.88521,-1.092774,2.897143\S,
0,4.357755,-2.99954,-1.014807\S,0,6.201278,2.215557,-0.603929\O,0,7.31177,3.05558
1,-0.191383\O,0,6.502601,0.796342,-1.033599\O,0,4.775713,-1.770868,2.134746\O,0,6.
852863,-1.925537,3.587928\O,0,5.630117,-2.186238,-0.911933\O,0,4.417456,-4.414418,
-0.682886\C,0,3.940518,-2.923666,-2.832174\C,0,5.508867,3.020829,-2.159019\C,0,4.
989604,-0.13062,4.219868\F,0,4.222598,-1.710214,-3.323851\F,0,4.633985,-3.838102,
-3.504155\F,0,2.628663,-3.153023,-2.994178\F,0,5.862985,0.398185,5.072641\F,0,4.2
63936,0.853673,3.669372\F,0,4.160883,-0.949275,4.883394\F,0,5.127976,2.09907,-3.0
46439\F,0,4.447523,3.778754,-1.837731\F,0,6.439335,3.794009,-2.713058\H,0,1.82500
3,0.177375,-4.742687\C,0,-2.677399,4.25714,-1.291563\C,0,-3.889188,4.819064,-1.68
1526\C,0,-1.722817,5.012424,-0.606327\C,0,-4.147537,6.154847,-1.380523\H,0,-4.618
368,4.203033,-2.193659\C,0,-2.005544,6.339482,-0.29737\H,0,-0.774144,4.576771,-0.
313753\C,0,-3.216536,6.933573,-0.684768\H,0,-5.095654,6.594194,-1.677728\H,0,-1.2
70549,6.928589,0.24456\C,0,-3.494481,8.383225,-0.374916\H,0,-3.040179,9.039604,-1.
126987\H,0,-4.568119,8.59093,-0.365733\H,0,-3.081713,8.671174,0.596687\\Version=E
S64L-G16RevA.03\State=1-A\HF=-7074.4411825\RMSD=3.547e-09\Dipole=-11.0384972,0.48
73739,1.9257258\Quadrupole=-44.3082149,16.2598256,28.0483894,19.3771858,-27.65050
33,13.1130823\PG=C01 [X(C64H55F9N1O16P2Pd1S4Yb1)]\

int-4a-Yb

\0,1\C,0,1.733227,3.224242,1.638352\C,0,1.980933,3.493076,0.24574\C,0,1.333661,2.
946558,-0.806918\C,0,0.557563,2.770238,2.159068\C,0,0.384132,1.777264,-0.761908\C,
0,-0.647036,2.436172,1.45177\C,0,-0.790322,2.06098,0.14772\H,0,2.522082,3.499606,
2.332734\H,0,2.754693,4.226628,0.032241\H,0,1.538228,3.340225,-1.79876\H,0,0.4820
98,2.73834,3.244675\H,0,-0.026825,1.667064,-1.758422\H,0,-1.555776,2.468104,2.043
27\N,0,-2.101708,1.837973,-0.341287\S,0,-2.621764,2.54446,-1.735085\O,0,-1.572748,
2.69225,-2.769891\O,0,-3.872407,1.845952,-2.107183\O,0,-3.25522,-2.135121,2.26476
7\C,0,-4.220879,-3.031426,1.816172\C,0,-3.660556,-1.204553,3.210625\C,0,-4.261229,
-3.29616,0.441242\C,0,-5.092003,-3.649106,2.710175\C,0,-6.052585,-4.534162,2.2266
64\C,0,-5.254964,-4.168907,-0.021835\C,0,-6.143428,-4.783294,0.858494\H,0,-5.3491
93,-4.352941,-1.083774\H,0,-6.907245,-5.44972,0.471773\H,0,-6.735353,-5.015427,2.
919858\H,0,-5.02554,-3.421328,3.76751\C,0,-4.351659,-0.048982,2.80751\C,0,-3.3348
23,-1.442217,4.542987\C,0,-3.726166,-0.530984,5.522824\C,0,-4.75469,0.839947,3.81
7581\C,0,-4.450711,0.603214,5.158027\H,0,-5.298586,1.736086,3.547264\H,0,-4.77477
7,1.311534,5.913669\H,0,-2.778495,-2.339696,4.790451\H,0,-3.472755,-0.712769,6.56
2346\P,0,-3.048151,-2.466015,-0.67275\P,0,-4.580796,0.331599,1.012212\C,0,-1.5368
21,-3.493316,-0.494865\C,0,-3.670753,-2.811253,-2.353615\C,0,-6.074444,-0.586721,
0.492623\C,0,-5.205683,2.058597,1.092012\C,0,-6.95517,-1.210527,1.380687\C,0,-6.3
31749,-0.628676,-0.885564\C,0,-7.443096,-1.314174,-1.370166\C,0,-8.064382,-1.9002
83,0.891386\C,0,-8.307027,-1.958021,-0.481786\H,0,-6.766797,-1.170928,2.447737\H,
0,-5.663418,-0.113283,-1.565667\H,0,-7.625242,-1.352741,-2.439643\H,0,-8.734757,-
2.399437,1.584205\H,0,-9.167549,-2.502774,-0.858313\C,0,-1.042601,-3.79698,0.7835
76\C,0,-0.771608,-3.836955,-1.61904\C,0,0.462029,-4.468512,-1.464628\C,0,0.956043,
-4.754678,-0.191541\C,0,0.195241,-4.421445,0.929651\H,0,-1.12329,-3.582618,-2.611
381\H,0,1.052724,-4.712283,-2.341673\H,0,1.933928,-5.207923,-0.076789\H,0,0.57403
8,-4.635902,1.924345\H,0,-1.605414,-3.518881,1.664984\C,0,-3.725297,-4.11528,-2.8
73562\C,0,-4.111652,-1.739135,-3.140555\C,0,-4.634487,-1.972553,-4.41307\C,0,-4.2
44381,-4.343588,-4.146241\C,0,-4.708057,-3.271862,-4.914263\H,0,-3.353124,-4.9484
86,-2.286168\H,0,-4.012435,-0.723072,-2.775456\H,0,-4.963875,-1.132014,-5.015776\
H,0,-4.283344,-5.354846,-4.539344\H,0,-5.111105,-3.450813,-5.90661\C,0,-4.345972,
3.081024,1.520102\C,0,-6.544952,2.369925,0.823286\C,0,-7.013769,3.673537,0.99724\
C,0,-4.820058,4.37421,1.713118\C,0,-6.157374,4.674727,1.452612\H,0,-3.303205,2.86
2346,1.686521\H,0,-7.227561,1.598154,0.489698\H,0,-8.054041,3.899785,0.783091\H,0,
-4.134372,5.153625,2.027438\H,0,-6.523962,5.687511,1.588523\Pd,0,-2.684483,-0.215
525,-0.383338\C,0,1.136321,0.375973,-0.564944\C,0,0.230781,-0.875587,-0.909831\C,
0,-1.063784,-0.578956,-1.618836\H,0,0.019469,-1.395418,0.019159\H,0,0.836096,-1.5
62097,-1.501426\C,0,-1.195149,-0.474364,-2.904561\C,0,-1.355919,-0.311657,-4.1981
86\H,0,-1.285103,0.683071,-4.630996\H,0,-1.604562,-1.141311,-4.855226\C,0,2.24747
7,0.416636,-1.621894\C,0,1.681122,0.196554,0.8372\O,0,0.779189,-0.215936,1.695376
\O,0,1.710655,0.556577,-2.811425\O,0,3.466121,0.317286,-1.454297\O,0,2.850861,0.3
88876,1.189253\C,0,1.219464,-0.452756,3.066383\C,0,-0.009459,-0.891687,3.828691\H,
0,1.647162,0.477361,3.444377\H,0,2.006024,-1.209459,3.038717\H,0,-0.773587,-0.110
608,3.820745\H,0,0.258574,-1.109201,4.866712\H,0,-0.438263,-1.79185,3.380555\C,0,

2.530879,0.637619,-4.011555\C,0,2.520261,2.071293,-4.504751\H,0,3.531418,0.277549,-3.784156\H,0,1.494044,2.421048,-4.644888\H,0,3.046001,2.132321,-5.462621\H,0,3.027894,2.727603,-3.795531\Yb,0,4.823395,-0.306006,0.352797\O,0,3.207306,-2.130304,-0.204721\O,0,5.087161,2.062636,0.315493\O,0,6.360134,-0.060688,2.133801\S,0,5.704388,-1.007003,3.113195\S,0,4.24419,-2.991837,-0.862117\S,0,6.315499,2.08215,-0.560589\O,0,7.429647,2.916652,-0.148728\O,0,6.592423,0.629875,-0.885556\O,0,4.563852,-1.629906,2.344819\O,0,6.554625,-1.877726,3.903475\O,0,5.559265,-2.261054,-0.694\O,0,4.224536,-4.4193,-0.585235\C,0,3.900421,-2.824334,-2.707025\C,0,5.712225,2.804177,-2.19282\C,0,4.846971,0.130064,4.316107\F,0,4.62405,-1.830715,-3.230535\F,0,4.200697,-3.960763,-3.32846\F,0,2.598754,-2.55136,-2.905545\F,0,5.728266,0.648613,5.165634\F,0,4.235031,1.122348,3.654494\F,0,3.922534,-0.56041,4.997907\F,0,5.375653,1.829697,-3.04422\F,0,4.638551,3.577393,-1.976711\F,0,6.676604,3.539278,-2.739502\H,0,2.036763,-0.040687,-4.709138\C,0,-3.077484,4.216203,-1.282545\C,0,-4.364731,4.660481,-1.568795\C,0,-2.134703,5.068479,-0.701671\C,0,-4.713435,5.974745,-1.265144\H,0,-5.078868,3.971475,-2.002916\C,0,-2.507758,6.371581,-0.386146\H,0,-1.12892,4.721312,-0.495258\C,0,-3.797613,6.84702,-0.667201\H,0,-5.720006,6.322342,-1.480593\H,0,-1.782944,7.035726,0.077277\C,0,-4.177439,8.272269,-0.351522\H,0,-3.873259,8.949049,-1.159294\H,0,-5.258784,8.379042,-0.226533\H,0,-3.691067,8.622694,0.56388\\Version=ES64L-G16RevA.03\State=1-A\HF=-7074.4518425\RMSD=5.661e-10\Dipole=-9.6703939,-0.1685316,1.2674858\Quadrupole=-41.3799483,12.7327975,28.6471508,19.3893646,-34.3272273,13.6913597\PG=C01 [X(C64H55F9N1O16P2Pd1S4Yb1)]\

TS-3a-Yb

\0,1\C,0,-1.8831,4.061904,-3.985344\C,0,-2.441288,4.281163,-2.66756\C,0,-2.013694,3.838998,-1.470269\C,0,-0.644147,3.596646,-4.275921\C,0,-0.875141,2.92062,-1.100575\C,0,0.387961,3.164503,-3.364162\C,0,0.32332,2.867575,-2.042298\H,0,-2.505691,4.379963,-4.818367\H,0,-3.318633,4.925814,-2.647438\H,0,-2.556016,4.211231,-0.60441\H,0,-0.359934,3.572689,-5.325943\H,0,-0.461729,3.339835,-0.181731\H,0,1.377059,3.038705,-3.79445\N,0,1.472292,2.377183,-1.391633\S,0,2.577156,3.394412,-0.765106\O,0,3.517669,2.546052,0.005325\O,0,3.115615,4.312175,-1.781693\C,0,0.275169,6.285548,0.889683\C,0,1.104544,5.592332,0.008911\C,0,1.682799,4.392687,0.423598\C,0,1.438424,3.879463,1.698423\C,0,0.616511,4.590576,2.56767\C,0,0.023283,5.800573,2.179927\H,0,-0.17901,7.220001,0.571517\H,0,1.304836,5.965238,-0.988888\H,0,1.889412,2.942778,2.001067\H,0,0.439844,4.196873,3.563969\C,0,-0.829702,6.583671,3.147293\H,0,-0.217566,7.284042,3.728328\H,0,-1.334311,5.926171,3.861486\H,0,-1.590679,7.173332,2.627908\O,0,4.15239,-2.998558,0.015539\C,0,4.657672,-2.877646,1.298831\C,0,5.033646,-3.146284,-1.043682\C,0,3.982189,-1.997579,2.160636\C,0,5.750106,-3.62709,1.725965\C,0,6.195807,-3.497164,3.039827\C,0,4.438942,-1.900739,3.480829\C,0,5.541159,-2.634744,3.917894\H,0,3.932286,-1.238751,4.172625\H,0,5.882222,-2.534098,4.943172\H,0,7.052671,-4.073667,3.374263\H,0,6.246812,-4.293372,1.029879\C,0,5.653656,-2.014317,-1.605607\C,0,5.21789,-4.418805,-1.57591\C,0,6.052853,-4.589703,-2.680869\C,0,6.486217,-2.211584,-2.716344\C,0,6.690659,-3.485975,-3.246706\H,0,6.963928,-1.354276,-3.177123\H,0,7.339468,-3.612164,-4.107636\H,0,4.698386,-5.254394,-1.119153\H,0,6.199081,-5.581384,-3.097241\P,0,2.549263,-1.067828,1.473664\P,0,5.2

23794,-0.345697,-0.966875\C,0,1.284553,-2.395975,1.433967\C,0,2.045258,0.037185,2.
845739\C,0,6.335768,-0.088074,0.466917\C,0,5.898452,0.828571,-2.200576\C,0,7.3982
65,-0.939746,0.782334\C,0,6.085724,1.036971,1.270194\C,0,6.889576,1.291459,2.3792
96\C,0,8.192903,-0.68513,1.901483\C,0,7.939174,0.42728,2.702478\H,0,7.596474,-1.8
08748,0.164276\H,0,5.270823,1.706757,1.014848\H,0,6.694306,2.165504,2.994129\H,0,
9.006847,-1.360467,2.147636\H,0,8.557103,0.622319,3.573901\C,0,0.884727,-3.058553,
2.605581\C,0,0.776466,-2.816366,0.198582\C,0,-0.135385,-3.871924,0.137919\C,0,-0.
54585,-4.508785,1.30626\C,0,-0.032655,-4.102621,2.541017\H,0,1.097991,-2.315904,-
0.70785\H,0,-0.539181,-4.18118,-0.819223\H,0,-1.282689,-5.303466,1.263939\H,0,-0.
364586,-4.590567,3.451193\H,0,1.289899,-2.752932,3.565452\C,0,0.72032,0.100228,3.
30143\C,0,2.982986,0.951662,3.359439\C,0,2.614294,1.864232,4.345034\C,0,0.350285,
1.028824,4.276275\C,0,1.296756,1.902527,4.811738\H,0,-0.017956,-0.585781,2.906062
\H,0,4.00139,0.945315,2.985758\H,0,3.353317,2.554286,4.740786\H,0,-0.675962,1.048
354,4.626501\H,0,1.013381,2.609557,5.585884\C,0,4.997695,1.525284,-3.01715\C,0,7.
271165,1.101323,-2.298655\C,0,7.734323,2.040596,-3.218087\C,0,5.463089,2.474861,-
3.925948\C,0,6.830251,2.728915,-4.031527\H,0,3.931369,1.347171,-2.91479\H,0,7.971
568,0.5911,-1.64459\H,0,8.79811,2.24708,-3.28958\H,0,4.75359,3.031388,-4.52945\H,
0,7.192112,3.473948,-4.733791\Pd,0,2.928247,0.052174,-0.583173\C,0,-1.340602,1.49
9786,-0.616406\C,0,-0.144371,0.671165,-0.015011\C,0,0.969013,0.495347,-0.986444\C,
0,1.039071,-0.122256,-2.157038\C,0,1.101229,-0.301683,-3.466941\H,0,1.564258,-1.1
801,-3.91191\H,0,0.670289,0.426192,-4.155914\C,0,-2.310321,1.634711,0.56942\C,0,-
2.00881,0.646981,-1.685857\O,0,-2.144838,1.203699,-2.848838\O,0,-1.981376,2.63655
3,1.353859\O,0,-3.239739,0.867624,0.821953\O,0,-2.401976,-0.500814,-1.428113\C,0,
-2.974037,0.535362,-3.86674\C,0,-2.136436,-0.362105,-4.752037\H,0,-3.395904,1.378
699,-4.412725\H,0,-3.764806,-0.006934,-3.352113\H,0,-1.371873,0.209691,-5.283237\
H,0,-2.79389,-0.832491,-5.48977\H,0,-1.656938,-1.150921,-4.171932\C,0,-2.791751,2.
956674,2.526018\C,0,-3.591918,4.209682,2.230163\H,0,-2.056293,3.109546,3.317161\H,
0,-3.42311,2.103691,2.763265\H,0,-2.931543,5.022011,1.917987\H,0,-4.125878,4.5224
21,3.132806\H,0,-4.329052,4.024117,1.446546\H,0,-0.513057,-0.306363,0.310489\H,0,
0.218996,1.205446,0.86295\Yb,0,-4.11782,-1.131914,-0.06861\O,0,-4.797945,-2.04702
9,-2.186395\O,0,-4.637192,-1.817284,2.116576\O,0,-6.365922,-0.543632,0.12316\S,0,
-6.395899,0.648413,-0.810431\S,0,-4.445741,-3.48103,-1.871838\S,0,-3.261625,-2.29
888,2.526029\O,0,-3.144026,-3.668417,2.994968\O,0,-2.336161,-1.816546,1.439276\O,
0,-4.989938,0.776854,-1.335984\O,0,-7.491634,0.752989,-1.755534\O,0,-3.921967,-3.
442575,-0.45189\O,0,-5.404277,-4.513861,-2.21652\C,0,-2.925182,-3.828081,-2.89230
8\C,0,-2.834684,-1.254223,4.004746\C,0,-6.568133,2.096523,0.355962\F,0,-1.926907,
-3.002393,-2.547049\F,0,-2.530181,-5.084565,-2.687593\F,0,-3.206774,-3.647663,-4.
18404\F,0,-7.842934,2.266869,0.690004\F,0,-5.84797,1.886376,1.467822\F,0,-6.10984,
3.204504,-0.242765\F,0,-2.734803,0.043204,3.656461\F,0,-3.773658,-1.378045,4.9376
42\F,0,-1.656792,-1.64637,4.498927\\Version=ES64L-G16RevA.03\State=1-A\HF=-7074.3
895014\RMSD=2.369e-09\Dipole=4.5764316,2.8970064,1.4226448\Quadrupole=-29.5081482,
-3.1301856,32.6383337,-64.285357,-4.6303419,23.1218477\PG=C01 [X(C64H55F9N1O16P2P
d1S4Yb1)]\

int-5-Yb

\0,1\C,0,-0.535655,1.801277,4.640738\C,0,0.723643,2.221404,4.056955\C,0,1.005675,2.639695,2.809633\C,0,-1.765519,1.909905,4.090032\C,0,0.161014,2.70451,1.565029\C,0,-2.116875,2.364536,2.764921\C,0,-1.338232,2.652543,1.70851\H,0,-0.472907,1.422647,5.657748\H,0,1.560085,2.237335,4.752534\H,0,2.028582,2.972382,2.650341\H,0,-2.616771,1.614347,4.698119\H,0,0.377769,3.666498,1.083211\H,0,-3.175303,2.433406,2.563667\N,0,-2.018116,2.902505,0.467398\S,0,-2.413742,4.524412,0.080657\O,0,-3.468871,4.483341,-0.9322\O,0,-2.597673,5.20601,1.363047\C,0,1.058201,6.515139,-0.53425\C,0,-0.089724,6.001957,0.06365\C,0,-0.972175,5.240899,-0.704425\C,0,-0.724709,4.99808,-2.055735\C,0,0.436669,5.506622,-2.633172\C,0,1.342111,6.272228,-1.885701\H,0,1.740007,7.123665,0.053619\H,0,-0.319273,6.193822,1.105822\H,0,-1.433468,4.424275,-2.641536\H,0,0.638485,5.315115,-3.683215\C,0,2.575158,6.857855,-2.527014\H,0,2.872714,6.292924,-3.414749\H,0,3.418668,6.875752,-1.830343\H,0,2.395832,7.892224,-2.843561\O,0,-4.358091,-2.999164,0.588534\C,0,-4.722994,-3.322756,-0.709175\C,0,-5.367937,-2.693675,1.485906\C,0,-3.735676,-3.151845,-1.695184\C,0,-5.997472,-3.79392,-1.019599\C,0,-6.307164,-4.115501,-2.340119\C,0,-4.074735,-3.498564,-3.01175\C,0,-5.344392,-3.975859,-3.337079\H,0,-3.338903,-3.370428,-3.795629\H,0,-5.577268,-4.229885,-4.3663\H,0,-7.303397,-4.472462,-2.581693\H,0,-6.74229,-3.894432,-0.240158\C,0,-5.852125,-1.380436,1.566759\C,0,-5.871489,-3.720807,2.283064\C,0,-6.929358,-3.461605,3.15117\C,0,-6.948689,-1.159342,2.416697\C,0,-7.484173,-2.18108,3.198072\H,0,-7.378177,-0.166509,2.478869\H,0,-8.329584,-1.975386,3.847182\H,0,-5.441818,-4.712519,2.188743\H,0,-7.329776,-4.258175,3.770265\P,0,-2.185126,-2.223685,-1.299728\P,0,-5.032775,-0.028364,0.585902\C,0,-1.119843,-3.406585,-0.375428\C,0,-1.378739,-2.186334,-2.952182\C,0,-6.254826,0.150182,-0.78446\C,0,-5.364886,1.445304,1.632126\C,0,-7.496085,0.779762,-0.617451\C,0,-5.947149,-0.428798,-2.022378\C,0,-6.865067,-0.390391,-3.071885\C,0,-8.407629,0.830386,-1.670673\C,0,-8.09587,0.242579,-2.899291\H,0,-7.744293,1.243547,0.331306\H,0,-4.989946,-0.916563,-2.159016\H,0,-6.613041,-0.856965,-4.019152\H,0,-9.363186,1.327745,-1.532156\H,0,-8.809965,0.280413,-3.716634\C,0,-1.626275,-4.339898,0.541299\C,0,0.272344,-3.27493,-0.505825\C,0,1.136975,-4.05009,0.263689\C,0,0.625466,-4.954474,1.195395\C,0,-0.756482,-5.100085,1.324821\H,0,0.687636,-2.561481,-1.203612\H,0,2.206215,-3.925526,0.149898\H,0,1.300083,-5.536795,1.814828\H,0,-1.164593,-5.810994,2.037904\H,0,-2.693546,-4.460439,0.661867\C,0,-0.710498,-3.291594,-3.50026\C,0,-1.484952,-1.012165,-3.70612\C,0,-0.933734,-0.940518,-4.985618\C,0,-0.144074,-3.211996,-4.770621\C,0,-0.256595,-2.036689,-5.517332\H,0,-0.623501,-4.208444,-2.927489\H,0,-1.98583,-0.150656,-3.280966\H,0,-1.017736,-0.020396,-5.556665\H,0,0.384258,-4.068469,-5.179057\H,0,0.187569,-1.977141,-6.506657\C,0,-5.385852,1.394683,3.035545\C,0,-5.409135,2.700982,1.003457\C,0,-5.509167,3.8681,1.757084\C,0,-5.497598,2.566136,3.787128\C,0,-5.56268,3.80587,3.151046\H,0,-5.308831,0.442202,3.547459\H,0,-5.330111,2.77317,-0.075429\H,0,-5.512424,4.82664,1.251046\H,0,-5.520619,2.506371,4.871533\H,0,-5.632074,4.717987,3.735004\Pd,0,-2.787214,-0.113843,-0.315925\C,0,0.662623,1.663591,0.45632\C,0,-0.203927,1.789805,-0.842104\C,0,-1.688013,1.977753,-0.588805\H,0,-0.029912,0.925394,-1.485774\H,0,0.174422,2.67172,-1.364589\C,0,-2.650051,1.603571,-1.49713\C,0,-3.359265,1.886021,-2.577946\H,0,-4.126531,1.2226,-2.958986\H,0,-3.223079,

2.837455,-3.087183\C,0,2.106172,2.069634,0.182044\C,0,0.63909,0.229733,0.978056\O,
0,-0.351348,-0.026669,1.778747\O,0,2.215541,3.125819,-0.588752\O,0,3.087686,1.546
276,0.720949\O,0,1.473999,-0.619177,0.636151\C,0,-0.454401,-1.357958,2.405576\C,0,
-1.907294,-1.569113,2.757911\H,0,0.201846,-1.326858,3.276901\H,0,-0.082079,-2.096
906,1.701792\H,0,-2.26323,-0.7991,3.446306\H,0,-2.015284,-2.550873,3.227026\H,0,-
2.52771,-1.55781,1.855875\C,0,3.563975,3.606483,-0.902056\C,0,4.121575,4.443108,0.
232578\H,0,3.405746,4.192368,-1.806512\H,0,4.185817,2.740401,-1.125737\H,0,3.4343
36,5.25432,0.486625\H,0,5.075385,4.880037,-0.07926\H,0,4.303701,3.829827,1.117115
\Yb,0,3.791037,-0.654556,0.465633\O,0,5.869626,-1.426355,-0.382923\O,0,3.075057,-
2.014408,-1.369476\O,0,3.748516,-2.674359,1.635268\S,0,3.451041,-2.117833,3.01334
2\S,0,6.731316,-0.354871,0.241122\S,0,2.843793,-0.893381,-2.347805\O,0,1.511174,-
0.754643,-2.917549\O,0,3.411249,0.343525,-1.685709\O,0,3.409864,-0.619919,2.83254
6\O,0,2.369209,-2.734372,3.766911\O,0,5.759969,0.519844,1.007538\O,0,7.952494,-0.
761418,0.911595\C,0,7.247377,0.71612,-1.191261\C,0,3.992132,-1.240291,-3.767801\C,
0,5.026334,-2.428113,3.952174\F,0,6.16382,1.217665,-1.802319\F,0,7.995143,1.72508
4,-0.742867\F,0,7.943687,-0.00784,-2.063922\F,0,5.209601,-3.740121,4.087192\F,0,6.
050446,-1.899899,3.278822\F,0,4.941806,-1.858944,5.154275\F,0,5.244504,-1.356465,
-3.33154\F,0,3.61672,-2.371459,-4.3639\F,0,3.916723,-0.228757,-4.636466\\Version=
ES64L-G16RevC.01\State=1-A\HF=-7074.4346679\RMSD=1.425e-09\Dipole=-2.728614,2.142
1497,-0.5280598\Quadrupole=-20.2483152,6.4591071,13.7892082,55.9542477,-21.506938
6,-11.5519925\PG=C01 [X(C64H55F9N1O16P2Pd1S4Yb1)]\

int-4a'-Yb

\0,1\C,0,-2.291237,4.953364,-1.905564\C,0,-2.823683,4.614922,-0.61557\C,0,-2.2653
44,3.881941,0.37354\C,0,-0.993332,4.79644,-2.295168\C,0,-1.02318,3.028117,0.38189
3\C,0,0.122496,4.231649,-1.603121\C,0,0.173767,3.483099,-0.450668\H,0,-2.967342,5.
461729,-2.587886\H,0,-3.79513,5.048485,-0.383798\H,0,-2.815553,3.852177,1.312862\
H,0,-0.740004,5.201938,-3.274658\H,0,-0.628165,3.048666,1.400685\H,0,1.075008,4.4
02571,-2.08946\N,0,1.312225,3.015984,0.173896\S,0,2.7844,3.352552,-0.360441\O,0,3.
735711,2.567549,0.471792\O,0,2.999513,3.218191,-1.831538\C,0,4.099803,7.192737,-0.
547985\C,0,3.850499,5.857394,-0.859999\C,0,3.07396,5.089184,0.003787\C,0,2.540958,
5.643505,1.16759\C,0,2.794966,6.978952,1.464864\C,0,3.576452,7.773469,0.613788\H,
0,4.704642,7.796354,-1.219562\H,0,4.238336,5.407334,-1.766668\H,0,1.921313,5.0311
89,1.813712\H,0,2.377188,7.41662,2.367786\C,0,3.815482,9.229578,0.926778\H,0,2.98
1236,9.848488,0.574347\H,0,4.724078,9.598661,0.442937\H,0,3.910292,9.396464,2.003
996\O,0,3.889874,-3.579811,-0.161232\C,0,4.443812,-3.533697,1.104836\C,0,4.686583,
-3.706561,-1.284383\C,0,3.892021,-2.571335,1.960344\C,0,5.43825,-4.406323,1.52662
7\C,0,5.917687,-4.293449,2.832886\C,0,4.391619,-2.465895,3.259747\C,0,5.406242,-3.
321997,3.693303\H,0,3.985441,-1.714333,3.927583\H,0,5.793663,-3.229621,4.702423\H,
0,6.699868,-4.964415,3.173882\H,0,5.838228,-5.14407,0.839886\C,0,5.263338,-2.5640
15,-1.873215\C,0,4.785517,-4.963936,-1.872436\C,0,5.467754,-5.112197,-3.079374\C,
0,5.919712,-2.737375,-3.10192\C,0,6.027398,-3.994777,-3.696341\H,0,6.341066,-1.87
6891,-3.60567\H,0,6.5423,-4.094457,-4.646323\H,0,4.30205,-5.803198,-1.384279\H,0,
5.543992,-6.091792,-3.54016\P,0,2.50706,-1.582487,1.288533\P,0,5.009658,-0.899528,

-1.125698\C,0,1.247374,-2.906902,1.090351\C,0,1.961202,-0.540642,2.690509\C,0,6.0
34071,-0.833504,0.392246\C,0,5.989858,0.257201,-2.15751\C,0,7.063358,-1.738844,0.
66256\C,0,5.821359,0.262214,1.241799\C,0,6.625091,0.428878,2.367437\C,0,7.860642,
-1.566845,1.793993\C,0,7.641136,-0.486567,2.648861\H,0,7.232484,-2.584393,0.00434
1\H,0,5.051555,0.991278,1.009885\H,0,6.455764,1.278387,3.021944\H,0,8.647924,-2.2
82721,2.009838\H,0,8.262245,-0.357747,3.530226\C,0,1.152542,-3.936945,2.0411\C,0,
0.406733,-2.936142,-0.028947\C,0,-0.53582,-3.9533,-0.17973\C,0,-0.647286,-4.95313
4,0.78485\C,0,0.203598,-4.945548,1.892008\H,0,0.489656,-2.168686,-0.788862\H,0,-1.
199192,-3.944596,-1.035957\H,0,-1.404102,-5.72302,0.683141\H,0,0.125742,-5.72534,
2.643274\H,0,1.829009,-3.95889,2.889153\C,0,1.140774,-1.022545,3.72117\C,0,2.3337
36,0.812355,2.674501\C,0,1.89313,1.66901,3.683414\C,0,0.711299,-0.161291,4.73001\
C,0,1.086028,1.184998,4.713397\H,0,0.8079,-2.053091,3.721769\H,0,2.908361,1.22508,
1.851712\H,0,2.168965,2.717479,3.639427\H,0,0.076342,-0.542459,5.524725\H,0,0.739
138,1.854273,5.495519\C,0,5.428464,1.470551,-2.559298\C,0,7.353004,0.012277,-2.40
4864\C,0,8.115967,0.943741,-3.101938\C,0,6.202672,2.409801,-3.244663\C,0,7.539065,
2.143991,-3.530939\H,0,4.41399,1.727965,-2.293696\H,0,7.821082,-0.895956,-2.03988
9\H,0,9.164841,0.741774,-3.297194\H,0,5.747908,3.351339,-3.535453\H,0,8.14027,2.8
72786,-4.066332\Pd,0,2.768897,-0.422273,-0.72865\C,0,-1.429575,1.503084,0.228465\
C,0,-0.25236,0.483545,0.453622\C,0,0.681503,0.337357,-0.663746\C,0,1.095474,0.409
794,-1.846685\C,0,2.130525,0.372713,-2.764491\H,0,2.150277,-0.400269,-3.528845\H,
0,2.606187,1.320747,-2.993478\C,0,-2.399469,1.189904,1.381213\C,0,-2.070828,1.131
42,-1.100598\O,0,-1.829683,1.94827,-2.074111\O,0,-1.90821,1.571152,2.539201\O,0,-
3.485119,0.617065,1.286071\O,0,-2.695652,0.071243,-1.257081\C,0,-2.462961,1.68643
4,-3.373278\C,0,-1.689375,0.644476,-4.15755\H,0,-2.439884,2.669003,-3.841512\H,0,
-3.493357,1.384297,-3.182613\H,0,-0.642877,0.94049,-4.268974\H,0,-2.132102,0.5539
25,-5.154417\H,0,-1.740655,-0.332245,-3.672927\C,0,-2.676302,1.359818,3.765019\C,
0,-3.192333,2.697246,4.256498\H,0,-1.954988,0.913585,4.449745\H,0,-3.477937,0.652
839,3.562342\H,0,-2.373748,3.412324,4.374357\H,0,-3.677365,2.561913,5.228234\H,0,
-3.928614,3.109625,3.563617\H,0,-0.674449,-0.499435,0.686157\H,0,0.296226,0.83725
3,1.32398\Yb,0,-4.586336,-0.716148,-0.281128\O,0,-5.086528,-0.961817,-2.596794\O,
0,-4.887652,-1.959267,1.751318\O,0,-6.628989,0.248788,0.256248\S,0,-6.478436,1.57
5929,-0.469947\S,0,-5.381485,-2.44402,-2.563469\S,0,-3.645645,-2.80544,1.628602\O,
0,-3.782429,-4.248024,1.739735\O,0,-2.899204,-2.252737,0.440465\O,0,-5.082543,1.5
59085,-1.029673\O,0,-7.566597,1.989175,-1.336935\O,0,-5.383672,-2.800204,-1.09591
\O,0,-6.452056,-2.954453,-3.397948\C,0,-3.798477,-3.199523,-3.194204\C,0,-2.58560
2,-2.302069,3.09926\C,0,-6.416802,2.837251,0.899173\F,0,-2.751031,-2.638611,-2.55
9398\F,0,-3.787561,-4.510015,-2.962417\F,0,-3.676332,-2.970844,-4.501352\F,0,-7.5
99617,2.90174,1.505302\F,0,-5.481829,2.504548,1.802624\F,0,-6.111568,4.03034,0.38
3827\F,0,-1.740055,-1.314235,2.753974\F,0,-3.361154,-1.86538,4.095953\F,0,-1.8646
62,-3.336036,3.527473\\Version=ES64L-G16RevC.01\State=1-A\HF=-7074.4341414\RMSD=1.
483e-09\Dipole=8.6038668,-0.9764058,2.4865428\Quadrupole=-37.4372454,6.0467803,31.
3904651,-46.0928897,-27.0093827,10.1423576\PG=C01 [X(C64H55F9N1O16P2Pd1S4Yb1)]\

TS-3a'-Yb

\0,1\C,0,-2.334743,4.526219,-2.907094\C,0,-2.907752,4.323026,-1.595247\C,0,-2.389
357,3.700351,-0.521642\C,0,-1.054477,4.290264,-3.285056\C,0,-1.11682,2.916589,-0.
344054\C,0,0.034335,3.716388,-2.534001\C,0,0.035095,3.121669,-1.316017\H,0,-2.997
928,4.973173,-3.643382\H,0,-3.89231,4.761891,-1.450956\H,0,-2.991236,3.737188,0.3
84852\H,0,-0.790115,4.567245,-4.303693\H,0,-0.69632,3.212773,0.623487\H,0,0.99226
4,3.725133,-3.042117\N,0,1.186411,2.52278,-0.750616\S,0,2.633774,3.284603,-0.8276
65\O,0,3.563712,2.4977,0.014036\O,0,3.119283,3.6298,-2.182374\C,0,2.263493,7.2238
04,0.066332\C,0,2.564633,6.037411,-0.603487\C,0,2.263573,4.824571,0.006581\C,0,1.
669325,4.778676,1.270325\C,0,1.375542,5.969901,1.92287\C,0,1.664448,7.210143,1.33
0845\H,0,2.492787,8.175473,-0.404696\H,0,3.020236,6.039565,-1.586754\H,0,1.431311,
3.821964,1.720464\H,0,0.910513,5.942747,2.905001\C,0,1.30611,8.495127,2.033653\H,
0,0.241473,8.725638,1.90629\H,0,1.875195,9.339929,1.637169\H,0,1.497061,8.428703,
3.109114\O,0,4.153191,-3.271461,0.008454\C,0,4.687892,-3.133324,1.275899\C,0,4.97
236,-3.473501,-1.088056\C,0,4.084089,-2.151518,2.074235\C,0,5.71702,-3.935405,1.7
5285\C,0,6.179936,-3.73148,3.053746\C,0,4.568103,-1.957041,3.369651\C,0,5.616823,
-2.740172,3.856779\H,0,4.122824,-1.191597,3.995587\H,0,5.989055,-2.576421,4.86270
3\H,0,6.988846,-4.346923,3.434941\H,0,6.157192,-4.68929,1.109766\C,0,5.530451,-2.
367769,-1.758042\C,0,5.124896,-4.772186,-1.563582\C,0,5.852019,-4.99924,-2.731902
\C,0,6.239738,-2.621902,-2.941412\C,0,6.404967,-3.921514,-3.421507\H,0,6.657119,-
1.792644,-3.498889\H,0,6.958752,-4.086206,-4.340166\H,0,4.651589,-5.582914,-1.020
283\H,0,5.971043,-6.011189,-3.105636\P,0,2.676594,-1.246572,1.325606\P,0,5.192806,
-0.660803,-1.155124\C,0,1.461165,-2.622149,1.193475\C,0,2.078848,-0.181546,2.7006
41\C,0,6.23261,-0.45587,0.345168\C,0,6.125979,0.441202,-2.289944\C,0,7.284734,-1.
314298,0.675262\C,0,5.989422,0.679647,1.131808\C,0,6.78377,0.937134,2.246592\C,0,
8.073685,-1.054052,1.796063\C,0,7.823815,0.068767,2.584401\H,0,7.47831,-2.19398,0.
070737\H,0,5.192593,1.361372,0.857419\H,0,6.588105,1.817163,2.852048\H,0,8.877976,
-1.735492,2.056297\H,0,8.437215,0.266183,3.45844\C,0,1.351855,-3.573155,2.220909\
C,0,0.656948,-2.750195,0.052794\C,0,-0.281134,-3.779501,-0.029222\C,0,-0.419796,-
4.692638,1.015429\C,0,0.407424,-4.594533,2.135672\H,0,0.76576,-2.052866,-0.773823
\H,0,-0.922466,-3.849743,-0.900183\H,0,-1.175114,-5.469769,0.962951\H,0,0.313965,
-5.310754,2.946186\H,0,2.009881,-3.521971,3.082527\C,0,1.21331,-0.634344,3.705721
\C,0,2.50194,1.157367,2.712754\C,0,2.080692,2.018142,3.727462\C,0,0.7847,0.233817,
4.710495\C,0,1.220216,1.560241,4.727302\H,0,0.849995,-1.653209,3.694194\H,0,3.122
109,1.535737,1.908246\H,0,2.420956,3.049209,3.727866\H,0,0.110095,-0.131238,5.479
864\H,0,0.888963,2.232935,5.513177\C,0,5.47775,1.513761,-2.906506\C,0,7.516742,0.
299818,-2.443472\C,0,8.228964,1.191139,-3.240132\C,0,6.197775,2.418593,-3.689279\
C,0,7.568961,2.252831,-3.86784\H,0,4.423665,1.674405,-2.746954\H,0,8.043406,-0.49
6595,-1.928041\H,0,9.30142,1.068712,-3.358786\H,0,5.676486,3.257039,-4.139579\H,0,
8.129843,2.954736,-4.477818\Pd,0,2.933976,-0.200001,-0.767715\C,0,-1.42613,1.3871
4,-0.124728\C,0,-0.118279,0.580179,0.212443\C,0,0.917889,0.693841,-0.847594\C,0,1.
143337,0.044748,-2.00215\C,0,2.233413,0.388309,-2.824729\H,0,2.547793,-0.279074,-
3.624358\H,0,2.507316,1.435482,-2.964487\C,0,-2.30758,1.228646,1.117339\C,0,-2.07
1811,0.711149,-1.328978\O,0,-1.927934,1.343933,-2.451082\O,0,-1.811705,1.888402,2.
143273\O,0,-3.326916,0.546717,1.20857\O,0,-2.638209,-0.387901,-1.244913\C,0,-2.56

9205,0.784897,-3.650921\C,0,-1.753698,-0.354232,-4.230893\H,0,-2.610855,1.649879,
-4.311709\H,0,-3.577609,0.47745,-3.371235\H,0,-0.723048,-0.042122,-4.415626\H,0,-
2.206711,-0.659479,-5.17964\H,0,-1.746047,-1.21505,-3.56136\C,0,-2.463246,1.80811
3,3.450127\C,0,-2.789415,3.217542,3.897554\H,0,-1.727934,1.324278,4.094222\H,0,-3.
34624,1.17805,3.364664\H,0,-1.8935,3.844632,3.89711\H,0,-3.193593,3.191212,4.9140
96\H,0,-3.539298,3.667629,3.242935\H,0,-0.385663,-0.468508,0.354812\H,0,0.266209,
0.975064,1.150957\Yb,0,-4.526607,-0.957138,-0.111175\O,0,-5.217762,-1.350042,-2.3
4751\O,0,-4.763642,-1.862584,2.090085\O,0,-6.48045,0.200656,0.395014\S,0,-6.29884
6,1.424789,-0.485164\S,0,-5.605612,-2.801811,-2.168315\S,0,-3.54747,-2.755869,2.0
45501\O,0,-3.691371,-4.135418,2.476313\O,0,-2.908286,-2.491704,0.706999\O,0,-4.92
8027,1.28723,-1.083492\O,0,-7.400623,1.790595,-1.356623\O,0,-5.508321,-3.040636,-
0.679571\O,0,-6.774746,-3.293341,-2.871265\C,0,-4.129578,-3.704385,-2.856873\C,0,
-2.350374,-1.975504,3.271568\C,0,-6.145388,2.833285,0.725619\F,0,-3.016198,-3.246
254,-2.256099\F,0,-4.239531,-5.010308,-2.632945\F,0,-4.033827,-3.476313,-4.166919
\F,0,-7.293566,2.998799,1.37523\F,0,-5.170614,2.595635,1.620983\F,0,-5.841945,3.9
53821,0.061166\F,0,-1.538245,-1.105598,2.64776\F,0,-3.024355,-1.318466,4.221599\F,
0,-1.598021,-2.914161,3.844022\\Version=ES64L-G16RevC.01\State=1-A\HF=-7074.39992
64\RMSD=2.115e-09\Dipole=7.689553,2.2329446,3.0623388\Quadrupole=-42.389354,17.17
39826,25.2153713,-49.7482595,-11.5998315,18.3137232\PG=C01 [X(C64H55F9N1O16P2Pd1S
4Yb1)]\

product-Yb

\0,1\C,0,-3.118577,4.439989,-1.429193\C,0,-2.390681,3.397895,-2.126668\C,0,-2.343
683,2.074132,-1.899901\C,0,-4.009778,4.285618,-0.425064\C,0,-2.945019,1.200947,-0.
83033\C,0,-4.391411,3.074178,0.265829\C,0,-3.94564,1.807725,0.140492\H,0,-2.95183
7,5.448813,-1.797446\H,0,-1.825325,3.742648,-2.989867\H,0,-1.771042,1.500322,-2.6
27451\H,0,-4.4969,5.183424,-0.053743\H,0,-3.485455,0.396992,-1.349378\H,0,-5.1386
15,3.202614,1.041203\N,0,-4.487432,0.850292,1.06151\S,0,-6.078868,0.24779,0.80830
7\O,0,-6.368805,-0.616658,1.948841\O,0,-6.904078,1.403573,0.464892\C,0,-5.905699,
-0.989137,-3.039126\C,0,-6.076052,-0.193761,-1.908415\C,0,-5.941236,-0.776678,-0.
647131\C,0,-5.638821,-2.131556,-0.503391\C,0,-5.476063,-2.910377,-1.646541\C,0,-5.
614405,-2.356247,-2.927275\H,0,-6.01782,-0.546106,-4.024691\H,0,-6.322809,0.85858
1,-1.991232\H,0,-5.554179,-2.560606,0.488706\H,0,-5.254864,-3.96906,-1.54389\C,0,
-5.505722,-3.222003,-4.156772\H,0,-4.845463,-4.077091,-3.99012\H,0,-5.128632,-2.6
57693,-5.01429\H,0,-6.489342,-3.61821,-4.435781\C,0,-1.81638,0.38818,-0.068436\C,
0,-2.513462,-0.668806,0.853081\C,0,-3.541634,-0.0049,1.726174\H,0,-1.777654,-1.19
6564,1.458482\H,0,-2.999585,-1.387211,0.186573\C,0,-3.597668,-0.169555,3.026852\C,
0,-3.666144,-0.390639,4.30756\H,0,-4.2841,-1.192933,4.703693\H,0,-3.113782,0.2158
01,5.020998\C,0,-0.975574,-0.355525,-1.101067\C,0,-0.910566,1.251574,0.806607\O,0,
-1.411893,2.410059,1.121931\O,0,-1.691847,-1.180892,-1.830808\O,0,0.233255,-0.207
583,-1.287374\O,0,0.171141,0.849335,1.24789\C,0,-0.645564,3.27365,2.032969\C,0,-1.
523797,4.466958,2.332143\H,0,0.284523,3.542333,1.530808\H,0,-0.406522,2.683054,2.
920121\H,0,-1.778085,4.999164,1.412584\H,0,-0.984949,5.149295,2.99553\H,0,-2.4505
76,4.159386,2.823645\C,0,-0.968136,-2.010831,-2.799241\C,0,-2.00514,-2.852373,-3.

506124\H,0,-0.242173,-2.605109,-2.239796\H,0,-0.424772,-1.345511,-3.473644\H,0,-2.
548425,-3.477878,-2.793616\H,0,-1.510778,-3.500435,-4.235096\H,0,-2.726097,-2.221
519,-4.031277\Yb,0,2.00901,-0.165054,0.218823\O,0,3.469349,1.409647,1.0945\O,0,3.
688617,-1.820459,0.016294\O,0,0.629811,-2.092346,0.675949\S,0,0.911432,-2.13195,2.
165679\S,0,2.970519,2.641597,0.359875\S,0,4.030504,-1.559846,-1.432868\O,0,4.0896
21,-2.698179,-2.333834\O,0,3.12798,-0.409062,-1.831005\O,0,1.9886,-1.103782,2.401
289\O,0,-0.25919,-2.094898,3.035522\O,0,1.911885,2.138945,-0.584534\O,0,2.641532,
3.803551,1.175809\C,0,4.395766,3.156,-0.725641\C,0,5.74103,-0.823096,-1.375168\C,
0,1.716797,-3.784022,2.463401\F,0,4.623688,2.230529,-1.656664\F,0,4.083228,4.3088
46,-1.318058\F,0,5.483667,3.315035,0.024924\F,0,0.827385,-4.740275,2.181071\F,0,2.
7872,-3.932003,1.693154\F,0,2.068258,-3.863903,3.746248\F,0,5.810668,0.082317,-0.
397205\F,0,6.623833,-1.792401,-1.14354\F,0,6.00773,-0.239284,-2.542788\\Version=E
S64L-G16RevC.01\State=1-A\HF=-4798.9650794\RMSD=3.193e-09\Dipole=-2.7109364,0.095
3314,-2.9036242\Quadrupole=-34.8343353,16.8325343,18.0018009,11.858274,23.0338317,
11.0521782\PG=C01 [X(C28H27F9N1O15S4Yb1)]\

int-IV+CO_3^{2-}

\-2,1\C,0,6.055056,-2.29558,0.053235\C,0,6.729344,-1.0302,-0.139151\C,0,6.3193,0.
207327,0.192754\C,0,4.98576,-2.540493,0.845268\C,0,5.008834,0.674138,0.767628\C,0,
4.209683,-1.643,1.683883\C,0,4.222822,-0.283703,1.637416\H,0,6.482519,-3.14507,-0.
475868\H,0,7.710948,-1.099346,-0.610256\H,0,7.003472,1.024508,-0.017498\H,0,4.638
934,-3.570387,0.881794\H,0,5.235959,1.509673,1.441283\O,0,-3.762542,0.349889,-1.4
54988\C,0,-4.547395,0.270424,-0.333104\C,0,-3.644477,-0.69883,-2.357116\C,0,-4.14
436,1.112347,0.726792\C,0,-5.660562,-0.557398,-0.232978\C,0,-6.408787,-0.554321,0.
946333\C,0,-4.917637,1.094494,1.894125\C,0,-6.043358,0.274413,2.006003\H,0,-4.620
217,1.715543,2.732602\H,0,-6.619832,0.270325,2.927824\H,0,-7.263352,-1.219188,1.0
37361\H,0,-5.909087,-1.2173,-1.056043\C,0,-2.608625,-1.630805,-2.199353\C,0,-4.49
9532,-0.716453,-3.456282\C,0,-4.346483,-1.698755,-4.435212\C,0,-2.465113,-2.59623
8,-3.204478\C,0,-3.321562,-2.637175,-4.304636\H,0,-1.656483,-3.313653,-3.120875\H,
0,-3.179534,-3.398894,-5.067832\H,0,-5.262036,0.052689,-3.532506\H,0,-5.01145,-1.
718907,-5.29503\P,0,-2.481888,1.883702,0.53812\P,0,-1.536079,-1.56378,-0.690662\P
d,0,-0.596862,0.375607,0.422994\C,0,4.028865,1.313011,-0.294691\C,0,2.721255,1.79
1414,0.432002\C,0,2.246108,0.858416,1.497479\H,0,2.95506,2.767557,0.886115\H,0,1.
923381,1.912792,-0.314137\C,0,1.024522,0.395692,1.656113\C,0,0.083362,-0.378822,2.
339935\H,0,0.162811,-1.462398,2.330723\H,0,-0.40007,0.028871,3.230096\C,0,4.59021
4,2.574108,-0.940362\C,0,3.677964,0.242793,-1.331289\O,0,4.335873,0.419163,-2.487
879\O,0,5.697374,3.055832,-0.281199\O,0,4.096901,3.186695,-1.855033\O,0,2.962408,
-0.698101,-1.075504\C,0,3.911254,-0.43215,-3.583481\C,0,4.49317,-1.832482,-3.4518
08\H,0,4.303878,0.076435,-4.469362\H,0,2.814572,-0.391087,-3.618658\H,0,5.588548,
-1.805381,-3.401154\H,0,4.200928,-2.438251,-4.318492\H,0,4.118578,-2.30959,-2.545
27\C,0,6.18427,4.310519,-0.766262\C,0,7.380614,4.688848,0.088626\H,0,5.385958,5.0
58146,-0.70601\H,0,6.451529,4.213808,-1.824344\H,0,7.091992,4.769508,1.140982\H,0,
7.790517,5.652245,-0.234394\H,0,8.168452,3.933193,0.009901\O,0,-0.288787,-0.54398
3,-2.577437\C,0,0.430169,0.533109,-2.449312\O,0,1.245342,0.908635,-3.336806\O,0,0.

299785,1.266872,-1.346297\C,0,-2.848673,-2.400862,0.413263\C,0,-3.729107,-3.40170
7,-0.032443\C,0,-2.980193,-1.958023,1.735345\C,0,-4.695144,-3.947173,0.812635\H,0,
-3.657176,-3.756399,-1.056329\C,0,-3.946611,-2.498456,2.586297\H,0,-2.321919,-1.1
62711,2.072898\C,0,-4.806776,-3.496977,2.130331\H,0,-5.365829,-4.720302,0.441546\
H,0,-4.038791,-2.121783,3.602076\H,0,-5.566912,-3.911385,2.789452\C,0,-2.813859,3.
074932,-0.840892\C,0,-1.739117,3.387871,-1.687022\C,0,-4.072624,3.644099,-1.08419
2\C,0,-1.92804,4.290321,-2.737727\H,0,-0.788917,2.859465,-1.561766\C,0,-4.253033,
4.544126,-2.133771\H,0,-4.919931,3.359513,-0.466093\C,0,-3.174982,4.87636,-2.9592
65\H,0,-1.093612,4.511274,-3.397992\H,0,-5.234875,4.978375,-2.313636\H,0,-3.31522
7,5.573166,-3.783477\C,0,-0.407742,-3.042853,-0.873806\C,0,0.742363,-2.949612,-1.
682331\C,0,-0.598539,-4.227851,-0.140125\C,0,1.645087,-4.009575,-1.76122\H,0,0.87
9979,-2.03022,-2.243697\C,0,0.309181,-5.286668,-0.223214\H,0,-1.460184,-4.335027,
0.505907\C,0,1.438112,-5.185245,-1.035627\H,0,2.523335,-3.906146,-2.392218\H,0,0.
130465,-6.190244,0.35667\H,0,2.149574,-6.006399,-1.095847\C,0,-2.44331,3.065819,1.
978562\C,0,-3.444375,4.003793,2.274287\C,0,-1.299591,3.029782,2.787977\C,0,-3.315
022,4.869789,3.359899\H,0,-4.326983,4.061169,1.645811\C,0,-1.165844,3.898541,3.87
42\H,0,-0.512352,2.324123,2.534748\C,0,-2.174276,4.817062,4.166153\H,0,-4.100959,
5.591078,3.574198\H,0,-0.268768,3.856913,4.4868\H,0,-2.071628,5.494685,5.010967\O,
0,3.316833,0.415116,2.395309\C,0,3.213099,-2.313572,2.571803\C,0,3.196982,-2.0808
02,3.953975\C,0,2.279496,-3.208861,2.031025\C,0,2.277925,-2.727253,4.776914\H,0,3.
903015,-1.371581,4.37337\C,0,1.360927,-3.859396,2.855803\H,0,2.239529,-3.360248,0.
957583\C,0,1.357405,-3.624815,4.230903\H,0,2.276346,-2.526236,5.845951\H,0,0.6325
13,-4.525235,2.406957\H,0,0.632656,-4.124024,4.869385\\Version=ES64L-G16RevA.03\S
tate=1-A\HF=-3845.2675922\RMSD=6.761e-09\Dipole=-1.2025595,-0.5829505,3.8097032\Q
uadrupole=3.7721542,7.7255465,-11.4977007,10.3857682,-3.9825343,16.0094098\PG=C01
[X(C61H52O9P2Pd1)]\

TS-IV

\-2,1\C,0,7.308958,-0.881904,0.414983\C,0,7.291794,0.514581,0.054773\C,0,6.296184,
1.413924,0.173493\C,0,6.335535,-1.568851,1.07097\C,0,4.878805,1.294604,0.657193\C,
0,5.038977,-1.168387,1.559541\C,0,4.425381,0.058844,1.399803\H,0,8.203876,-1.4363
18,0.137827\H,0,8.224908,0.893642,-0.366161\H,0,6.530139,2.431145,-0.136015\H,0,6.
542553,-2.624148,1.241036\H,0,4.699907,2.129012,1.346264\O,0,-4.034405,0.145369,-
1.287938\C,0,-4.677611,-0.07733,-0.095737\C,0,-3.868635,-0.86237,-2.224081\C,0,-4.
258004,0.746068,0.971503\C,0,-5.679312,-1.030564,0.05744\C,0,-6.300582,-1.174831,
1.299831\C,0,-4.905191,0.580972,2.203118\C,0,-5.919665,-0.365123,2.369101\H,0,-4.
590971,1.18545,3.047528\H,0,-6.395359,-0.481675,3.339556\H,0,-7.066029,-1.934635,
1.429919\H,0,-5.941257,-1.666053,-0.78105\C,0,-2.796577,-1.762114,-2.115869\C,0,-
4.74189,-0.878396,-3.308394\C,0,-4.5688,-1.818297,-4.324016\C,0,-2.640016,-2.6889
4,-3.154161\C,0,-3.512341,-2.724881,-4.242372\H,0,-1.804062,-3.378071,-3.113614\H,
0,-3.35603,-3.455376,-5.032208\H,0,-5.533539,-0.13661,-3.343458\H,0,-5.246472,-1.
831545,-5.173801\P,0,-2.698223,1.686335,0.701669\P,0,-1.671577,-1.676118,-0.64281
7\Pd,0,-0.81684,0.287925,0.40216\C,0,3.780681,1.582077,-0.482391\C,0,2.409477,1.7
78988,0.162873\C,0,2.106383,0.84392,1.208673\H,0,2.232357,2.817252,0.447005\H,0,1.

151553,1.515545,-0.947651\C,0,0.914066,0.425978,1.623618\C,0,0.083886,-0.432628,2.
306477\H,0,0.221417,-1.512228,2.256407\H,0,-0.504611,-0.089352,3.160874\C,0,4.107
378,2.871148,-1.218858\C,0,3.738782,0.336103,-1.365789\O,0,4.469292,0.41362,-2.48
8433\O,0,4.434194,3.854641,-0.30967\O,0,4.05497,3.117327,-2.399242\O,0,3.183165,-
0.67974,-0.996068\C,0,4.299851,-0.69331,-3.395786\C,0,5.103032,-1.911926,-2.95714
\H,0,4.658135,-0.306657,-4.354845\H,0,3.22789,-0.890648,-3.472628\H,0,6.150593,-1.
642858,-2.783674\H,0,5.065529,-2.687689,-3.733169\H,0,4.693449,-2.315437,-2.03030
6\C,0,4.633781,5.1521,-0.869396\C,0,4.924231,6.095786,0.284454\H,0,3.738747,5.452
441,-1.425167\H,0,5.461411,5.121909,-1.588511\H,0,4.087246,6.106381,0.988709\H,0,
5.083391,7.115375,-0.084674\H,0,5.820043,5.778191,0.8274\O,0,-0.701661,0.011298,-
2.969537\C,0,0.349513,0.602283,-2.605872\O,0,1.46822,0.645032,-3.153538\O,0,0.220
566,1.306368,-1.384731\C,0,-2.740696,-2.69698,0.50113\C,0,-3.459263,-3.82499,0.07
5617\C,0,-2.849676,-2.300459,1.838841\C,0,-4.264555,-4.536243,0.963508\H,0,-3.392
482,-4.139586,-0.961181\C,0,-3.654789,-3.011421,2.730596\H,0,-2.308441,-1.413915,
2.153638\C,0,-4.364266,-4.130715,2.297162\H,0,-4.817619,-5.40602,0.615117\H,0,-3.
740315,-2.675692,3.760582\H,0,-4.998402,-4.679972,2.989546\C,0,-3.219379,2.878638,
-0.615006\C,0,-2.23631,3.267657,-1.535843\C,0,-4.524437,3.371454,-0.753054\C,0,-2.
55515,4.169282,-2.554038\H,0,-1.246329,2.817111,-1.478717\C,0,-4.839231,4.268049,
-1.773467\H,0,-5.302359,3.02869,-0.07618\C,0,-3.849959,4.676285,-2.67242\H,0,-1.7
88822,4.454212,-3.269895\H,0,-5.856481,4.641742,-1.873162\H,0,-4.094954,5.371229,
-3.472828\C,0,-0.363809,-2.910186,-1.06556\C,0,0.529105,-2.624103,-2.116158\C,0,-
0.121587,-4.036556,-0.262508\C,0,1.628758,-3.447655,-2.34365\H,0,0.349066,-1.7507
41,-2.736939\C,0,0.986736,-4.852791,-0.495562\H,0,-0.793697,-4.273798,0.554988\C,
0,1.869494,-4.559736,-1.534175\H,0,2.317292,-3.196536,-3.144593\H,0,1.168612,-5.7
07438,0.151381\H,0,2.745436,-5.181773,-1.700623\C,0,-2.658876,2.815191,2.180442\C,
0,-3.721858,3.627739,2.603762\C,0,-1.447908,2.869373,2.885229\C,0,-3.584622,4.462
323,3.71254\H,0,-4.660834,3.609535,2.060014\C,0,-1.307637,3.708805,3.993587\H,0,-
0.616579,2.257947,2.538097\C,0,-2.375131,4.502941,4.412508\H,0,-4.41849,5.085625,
4.028473\H,0,-0.359828,3.740322,4.52454\H,0,-2.2669,5.155919,5.275643\O,0,3.22861
4,0.212793,1.977753\C,0,4.269584,-2.248168,2.245241\C,0,4.856138,-2.983506,3.2876
16\C,0,2.967009,-2.593232,1.839759\C,0,4.168617,-4.020155,3.921719\H,0,5.860994,-
2.722783,3.611681\C,0,2.277731,-3.619759,2.480969\H,0,2.510499,-2.0544,1.01733\C,
0,2.869906,-4.338052,3.52414\H,0,4.64445,-4.569054,4.73246\H,0,1.274172,-3.864353,
2.150193\H,0,2.323148,-5.138095,4.018495\\Version=ES64L-G16RevA.03\State=1-A\HF=-
3845.2452039\RMSD=5.367e-09\Dipole=-4.5147454,-0.7696945,3.0830392\Quadrupole=-20.
2321442,23.5916081,-3.3594639,7.7209385,-4.8489794,10.9640337\PG=C01 [X(C61H52O9P
2Pd1)]\

int-V

\-2,1\C,0,1.919693,-0.581051,3.061166\C,0,1.830311,-1.957718,3.473955\C,0,1.58865,
-3.032368,2.647758\C,0,2.383799,-0.188101,1.848469\C,0,1.338887,-3.028057,1.22883
9\C,0,1.888699,-2.039572,0.435248\H,0,1.563812,0.17875,3.755161\H,0,1.829574,-2.1
55365,4.545604\H,0,1.423622,-3.988998,3.141365\H,0,2.289377,0.834638,1.499263\C,0,
0.476828,-4.095058,0.671461\C,0,0.677565,-4.592558,-0.630534\C,0,-0.52485,-4.6950

77,1.455178\C,0,-0.076982,-5.656027,-1.112735\H,0,1.437006,-4.139078,-1.255169\C,
0,-1.272341,-5.768556,0.973103\H,0,-0.741651,-4.29277,2.437303\C,0,-1.048413,-6.2
60251,-0.311186\H,0,0.086465,-6.009143,-2.126803\H,0,-2.047768,-6.203286,1.599354
\H,0,-1.642781,-7.083815,-0.697701\O,0,1.400302,-1.794445,-0.784527\C,0,1.803992,
-0.633923,-1.491925\C,0,3.907743,-0.645998,-0.164212\C,0,3.028678,-1.218557,0.966
369\C,0,3.017442,-0.086377,-1.24735\H,0,3.302167,0.812287,-1.776793\C,0,4.872572,
0.43277,0.372419\C,0,4.66974,-1.76353,-0.865869\C,0,0.924101,0.030856,-3.638902\C,
0,0.743889,-0.104281,-2.313022\H,0,1.841478,-0.294984,-4.143014\H,0,0.186396,0.52
2425,-4.270917\O,0,4.802399,1.595365,0.065463\O,0,5.867984,0.084516,1.240967\O,0,
4.380971,-2.94711,-0.855384\O,0,5.716012,-1.260457,-1.570802\C,0,6.121474,-1.2274
39,1.755733\C,0,6.417297,-2.187524,-2.413885\H,0,5.587646,-2.001,1.204807\H,0,7.1
93338,-1.401254,1.604477\H,0,5.694837,-2.865771,-2.874981\H,0,6.877093,-1.566394,
-3.186452\C,0,7.463151,-2.963156,-1.624886\C,0,5.768043,-1.27383,3.235405\H,0,6.0
30361,-2.251111,3.656338\H,0,6.316622,-0.49838,3.778239\H,0,4.698387,-1.106736,3.
384066\H,0,8.04956,-3.602381,-2.294872\H,0,8.145912,-2.275831,-1.115621\H,0,6.975
086,-3.595699,-0.879994\H,0,3.602465,-1.897642,1.601597\O,0,-3.794315,1.893671,0.
060856\C,0,-4.683837,1.051718,-0.57147\C,0,-3.452163,1.697341,1.385117\C,0,-4.319
745,-0.257109,-0.962034\C,0,-5.92277,1.599755,-0.900353\C,0,-6.849481,0.857503,-1.
631801\C,0,-5.273946,-0.974102,-1.704823\C,0,-6.519311,-0.436162,-2.033433\H,0,-5.
027994,-1.975447,-2.038321\H,0,-7.225725,-1.029644,-2.608654\H,0,-7.812306,1.2910
37,-1.889059\H,0,-6.126626,2.618047,-0.58531\C,0,-2.131428,2.028935,1.745559\C,0,
-4.376927,1.207019,2.300823\C,0,-3.990007,0.997386,3.625408\C,0,-1.771453,1.78596
2,3.081126\C,0,-2.681578,1.279299,4.009936\H,0,-0.74874,1.969923,3.387721\H,0,-2.
357349,1.085626,5.028913\H,0,-5.37727,0.959442,1.961898\H,0,-4.700723,0.585941,4.
336093\P,0,-2.589071,-0.876711,-0.632386\P,0,-0.891461,2.342767,0.40938\C,0,-2.81
5118,-1.527243,1.090361\C,0,-2.631957,-2.456445,-1.620954\C,0,-1.444578,3.879626,
-0.458375\C,0,0.494888,3.186521,1.315389\C,0,-2.573678,4.637368,-0.126497\C,0,-0.
575105,4.346547,-1.457735\C,0,-0.81754,5.560222,-2.098086\C,0,-2.834813,5.835793,
-0.794708\C,0,-1.956766,6.302235,-1.776645\H,0,-3.242699,4.30144,0.658448\H,0,0.3
26121,3.805895,-1.731711\H,0,-0.072258,5.901113,-2.809906\H,0,-3.718972,6.414729,
-0.532473\H,0,-2.154484,7.24851,-2.276626\C,0,-4.022261,-2.048156,1.58194\C,0,-1.
705808,-1.478113,1.943821\C,0,-1.795879,-1.95605,3.251783\C,0,-2.993889,-2.492685,
3.723346\C,0,-4.11057,-2.534991,2.885156\H,0,-0.778443,-1.049686,1.575877\H,0,-0.
921476,-1.905918,3.891857\H,0,-3.063137,-2.862462,4.743853\H,0,-5.053717,-2.93549
8,3.250894\H,0,-4.901075,-2.053485,0.94381\C,0,-3.30118,-3.635413,-1.264118\C,0,-
1.932384,-2.419302,-2.836611\C,0,-1.950013,-3.516456,-3.6984\C,0,-3.320242,-4.730
619,-2.127969\C,0,-2.654801,-4.670769,-3.353393\H,0,-3.803286,-3.703474,-0.305649
\H,0,-1.343254,-1.535535,-3.069161\H,0,-1.397972,-3.470335,-4.633716\H,0,-3.84145
3,-5.638591,-1.834262\H,0,-2.66481,-5.528747,-4.022079\C,0,1.754186,3.134353,0.70
2197\C,0,0.309448,4.043506,2.412911\C,0,1.373934,4.791107,2.912512\C,0,2.80836,3.
926696,1.168503\C,0,2.622874,4.736326,2.287729\H,0,1.90344,2.533441,-0.190548\H,0,
-0.674735,4.15036,2.857263\H,0,1.21776,5.445938,3.768098\H,0,3.738262,3.953543,0.
613812\H,0,3.441446,5.352513,2.651792\Pd,0,-0.799514,0.488794,-0.952215\O,0,2.198
743,4.964082,-2.894951\C,0,3.136861,4.566741,-2.180388\O,0,3.994472,5.179653,-1.5

0105\O,0,3.257984,3.133509,-2.120436\H,0,4.003943,2.989447,-1.520717\\Version=ES64L-G16RevA.03\State=1-A\HF=-3845.3098071\RMSD=7.574e-09\Dipole=-5.0373524,-9.8188932,6.3883869\Quadrupole=26.6598967,-57.7647852,31.1048885,-53.367858,16.9073915,38.1091857\PG=C01 [X(C61H52O9P2Pd1)]\

TS-V

\-2,1\C,0,-2.229889,-0.804379,2.991625\C,0,-2.831956,0.347098,3.614685\C,0,-3.126213,1.53643,2.986797\C,0,-2.426101,-1.144405,1.693976\C,0,-2.937147,1.892125,1.605668\C,0,-2.972857,0.933355,0.612845\H,0,-1.542888,-1.399184,3.59236\H,0,-2.920959,0.333364,4.700609\H,0,-3.424716,2.360463,3.634487\H,0,-1.83345,-1.906561,1.196626\C,0,-2.69214,3.31543,1.273785\C,0,-3.251309,3.904713,0.124585\C,0,-1.918577,4.124804,2.121501\C,0,-3.050054,5.252839,-0.153693\H,0,-3.840948,3.29111,-0.545896\C,0,-1.712697,5.474899,1.838223\H,0,-1.437329,3.676001,2.981441\C,0,-2.282553,6.049165,0.702092\H,0,-3.487632,5.683565,-1.050498\H,0,-1.087197,6.069914,2.498936\H,0,-2.117569,7.099399,0.474193\O,0,-2.54299,1.225179,-0.614306\C,0,-2.326872,0.192481,-1.586166\C,0,-3.96522,-1.179933,-0.334808\C,0,-3.509456,-0.435891,0.931618\C,0,-2.904751,-1.017371,-1.401658\H,0,-2.612063,-1.832392,-2.062976\C,0,-4.253359,-2.662916,-0.037848\C,0,-5.189086,-0.504402,-0.942786\C,0,-1.476157,1.815317,-3.146447\C,0,-1.457544,0.558815,-2.66862\H,0,-0.500553,-0.498329,-3.031065\H,0,-2.132603,2.610132,-2.77181\H,0,-0.796791,2.124411,-3.939484\O,0,-3.599135,-3.567411,-0.48402\O,0,-5.307356,-3.003376,0.771033\O,0,-5.564589,0.634293,-0.722934\O,0,-5.807066,-1.331403,-1.823845\C,0,-6.190892,-2.097548,1.439615\C,0,-6.866436,-0.75169,-2.60011\H,0,-6.117435,-1.081678,1.053102\H,0,-7.203926,-2.455968,1.220358\H,0,-6.589947,0.269999,-2.872156\H,0,-6.916822,-1.365964,-3.502422\C,0,-8.182624,-0.773254,-1.834772\C,0,-5.919357,-2.127767,2.937323\H,0,-6.642378,-1.496502,3.466545\H,0,-6.00365,-3.151207,3.315198\H,0,-4.913073,-1.76179,3.156118\H,0,-8.99867,-0.407483,-2.468712\H,0,-8.422613,-1.791807,-1.51364\H,0,-8.111931,-0.131107,-0.953951\H,0,-4.34454,-0.265232,1.617028\O,0,4.185369,-0.101997,0.514561\C,0,4.613346,1.149002,0.123916\C,0,3.562061,-0.317876,1.723597\C,0,3.729376,2.072438,-0.47256\C,0,5.978796,1.406904,0.247682\C,0,6.511039,2.611581,-0.208424\C,0,4.298609,3.277062,-0.919585\C,0,5.661176,3.549893,-0.793492\H,0,3.653419,4.013416,-1.384411\H,0,6.054776,4.496803,-1.155123\H,0,7.575502,2.808872,-0.111135\H,0,6.602562,0.638069,0.692533\C,0,2.640074,-1.385059,1.744809\C,0,3.852214,0.439146,2.853596\C,0,3.223001,0.130157,4.061394\C,0,2.026631,-1.667354,2.972749\C,0,2.315197,-0.925527,4.120966\H,0,1.299951,-2.470751,3.021083\H,0,1.812305,-1.161813,5.055317\H,0,4.544577,1.270298,2.776396\H,0,3.430609,0.730622,4.94256\P,0,1.903927,1.66957,-0.640338\P,0,2.137864,-2.101079,0.113486\C,0,1.433077,2.183214,1.087474\C,0,1.364367,3.148327,-1.616015\C,0,3.697358,-2.945107,-0.417265\C,0,1.144685,-3.572779,0.631169\C,0,4.793872,-3.160459,0.427838\C,0,3.754025,-3.378746,-1.752483\C,0,4.902554,-4.025009,-2.214543\C,0,5.940614,-3.799482,-0.046724\C,0,5.997704,-4.234315,-1.372303\H,0,4.754981,-2.821031,1.458907\H,0,2.884176,-3.223217,-2.404874\H,0,4.938949,-4.363167,-3.247609\H,0,6.787887,-3.956048,0.618597\H,0,6.891288,-4.731686,-1.745352\C,0,1.957098,3.331905,1.701115\C,0,0.611129,1.334675,1.834397\C,0,0.351565,1.60605,3.178355\C,0,0.891925,2.738729,3.784669\C,0,1.686371,3.612931,3.038251\H,0,0.21258,0.

443347,1.360994\H,0,-0.269339,0.926207,3.74622\H,0,0.695603,2.940389,4.835479\H,0,
2.106148,4.503387,3.501542\H,0,2.596362,3.998202,1.130447\C,0,0.469246,4.122137,-
1.154498\C,0,1.768791,3.195659,-2.962575\C,0,1.346836,4.220221,-3.802345\C,0,0.02
37,5.1359,-2.006673\C,0,0.469253,5.200259,-3.325542\H,0,0.102563,4.088539,-0.1349
8\H,0,2.401691,2.400456,-3.34853\H,0,1.678044,4.239505,-4.838012\H,0,-0.680085,5.
87006,-1.62738\H,0,0.118887,5.990336,-3.985765\C,0,-0.11072,-3.758581,0.037185\C,
0,1.625446,-4.531939,1.53751\C,0,0.848453,-5.636085,1.880465\C,0,-0.886767,-4.872
267,0.377838\C,0,-0.415119,-5.801636,1.303416\H,0,-0.4792,-3.073054,-0.722305\H,0,
2.615298,-4.411188,1.969106\H,0,1.228845,-6.369673,2.588997\H,0,-1.856496,-4.9898
32,-0.089906\H,0,-1.023834,-6.664061,1.567766\Pd,0,1.416897,-0.413083,-1.259324\O,
0,0.423783,-1.194306,-3.118976\C,0,0.119579,-2.558562,-3.088126\O,0,1.106906,-3.3
39154,-3.142933\O,0,-1.097723,-2.869526,-2.994739\\Version=ES64L-G16RevA.03\State
=1-A\HF=-3845.2877576\RMSD=4.891e-09\Dipole=-0.5805262,5.9024095,8.2376141\Quadru
pole=26.136961,-24.8823117,-1.2546492,13.102796,3.0932923,-33.901202\PG=C01 [X(C6
1H52O9P2Pd1)]\

5a+PdL$_n$+CO$_3^{2-}$

\-2,1\C,0,-2.002504,-0.753274,2.889642\C,0,-2.627635,0.353263,3.571814\C,0,-2.997
745,1.544867,2.995767\C,0,-2.242729,-1.068879,1.595118\C,0,-2.883053,1.943502,1.6
1513\C,0,-2.940772,1.012926,0.605082\H,0,-1.254752,-1.323183,3.438473\H,0,-2.6668
31,0.304834,4.659601\H,0,-3.294455,2.340575,3.678406\H,0,-1.637977,-1.789762,1.05
3035\C,0,-2.659069,3.377532,1.312865\C,0,-3.30266,4.006259,0.231815\C,0,-1.803474,
4.148117,2.115946\C,0,-3.097183,5.357651,-0.030105\H,0,-3.959407,3.419943,-0.4005
54\C,0,-1.596503,5.501663,1.850786\H,0,-1.25976,3.666777,2.919498\C,0,-2.245542,6.
115452,0.779354\H,0,-3.599216,5.821608,-0.875163\H,0,-0.906035,6.06682,2.471293\H,
0,-2.078452,7.168086,0.565066\O,0,-2.593228,1.363525,-0.642649\C,0,-2.340552,0.36
5346,-1.598291\C,0,-3.8565,-1.118251,-0.373319\C,0,-3.390813,-0.390175,0.900178\C,
0,-2.837727,-0.878472,-1.472469\H,0,-2.476086,-1.666888,-2.165337\C,0,-4.086696,-
2.621166,-0.120218\C,0,-5.130248,-0.473414,-0.917107\C,0,-1.47056,2.139664,-3.050
659\C,0,-1.549075,0.843802,-2.720329\H,0,-1.010027,0.04296,-3.244361\H,0,-1.94388
3,2.921562,-2.465087\H,0,-0.867877,2.45745,-3.893595\O,0,-3.43076,-3.484848,-0.63
3148\O,0,-5.09341,-3.020927,0.723472\O,0,-5.550052,0.634412,-0.634104\O,0,-5.7348
86,-1.291051,-1.809821\C,0,-5.976308,-2.172537,1.463244\C,0,-6.871236,-0.750079,-
2.502655\H,0,-5.958803,-1.141723,1.110454\H,0,-6.985041,-2.560969,1.277513\H,0,-6.
678259,0.299892,-2.736425\H,0,-6.927129,-1.324731,-3.430004\C,0,-8.138986,-0.8999
09,-1.673299\C,0,-5.635623,-2.244151,2.945428\H,0,-6.353696,-1.656745,3.529085\H,
0,-5.668452,-3.282298,3.289049\H,0,-4.632462,-1.852937,3.132306\H,0,-9.010946,-0.
565533,-2.246994\H,0,-8.289544,-1.946352,-1.390577\H,0,-8.066891,-0.294675,-0.766
742\H,0,-4.205206,-0.284836,1.622593\O,0,4.119152,-0.230647,0.444809\C,0,4.592851,
1.000603,0.050511\C,0,3.510187,-0.42517,1.664084\C,0,3.732495,1.965803,-0.512172\
C,0,5.971942,1.194195,0.133153\C,0,6.544007,2.378042,-0.329228\C,0,4.341825,3.149
884,-0.962462\C,0,5.718571,3.36007,-0.877007\H,0,3.718274,3.921504,-1.398389\H,0,
6.142614,4.292907,-1.241304\H,0,7.618916,2.526402,-0.264534\H,0,6.572652,0.392484,
0.55085\C,0,2.543012,-1.45241,1.695487\C,0,3.86096,0.308555,2.792182\C,0,3.243481,

0.022889,4.012206\C,0,1.940851,-1.708945,2.936351\C,0,2.285419,-0.987195,4.082018
\H,0,1.185672,-2.484658,2.998665\H,0,1.791438,-1.207491,5.025357\H,0,4.593277,1.1
03814,2.70466\H,0,3.499316,0.605189,4.892922\P,0,1.885145,1.63357,-0.621056\P,0,1.
984428,-2.150827,0.072236\C,0,1.500737,2.199372,1.120952\C,0,1.389439,3.15232,-1.
568661\C,0,3.535629,-2.987289,-0.509177\C,0,1.027768,-3.634921,0.634851\C,0,4.653
006,-3.204456,0.308024\C,0,3.563055,-3.41023,-1.848354\C,0,4.707595,-4.039002,-2.
343794\C,0,5.793596,-3.829695,-0.198978\C,0,5.824944,-4.24731,-1.530852\H,0,4.638
696,-2.87708,1.343479\H,0,2.649847,-3.278407,-2.451782\H,0,4.722955,-4.368996,-3.
380513\H,0,6.656066,-3.988038,0.446412\H,0,6.713841,-4.733697,-1.929724\C,0,2.078
306,3.346496,1.688662\C,0,0.691429,1.389011,1.920566\C,0,0.493692,1.696175,3.2681
33\C,0,1.081893,2.829729,3.826435\C,0,1.867205,3.666063,3.027893\H,0,0.255557,0.4
97205,1.482952\H,0,-0.11351,1.041263,3.878929\H,0,0.933432,3.059689,4.879334\H,0,
2.327253,4.555801,3.453149\H,0,2.710733,3.98338,1.078185\C,0,0.521548,4.141991,-1.
08425\C,0,1.786787,3.222911,-2.917594\C,0,1.391166,4.27947,-3.730311\C,0,0.098317,
5.187186,-1.909686\C,0,0.540724,5.272818,-3.228829\H,0,0.161695,4.098339,-0.06293
6\H,0,2.398635,2.421412,-3.324211\H,0,1.724349,4.316893,-4.765149\H,0,-0.584249,5.
930757,-1.508688\H,0,0.211194,6.088266,-3.86866\C,0,-0.235216,-3.869614,0.074017\
C,0,1.550523,-4.556092,1.55662\C,0,0.80908,-5.66708,1.954309\C,0,-0.978489,-4.985
302,0.476416\C,0,-0.464776,-5.8766,1.417112\H,0,-0.630665,-3.220478,-0.70099\H,0,
2.546328,-4.399803,1.961772\H,0,1.224923,-6.36911,2.674985\H,0,-1.957858,-5.13879
2,0.039316\H,0,-1.049027,-6.742176,1.724285\Pd,0,1.28373,-0.382408,-1.286143\O,0,
0.415011,-1.25434,-3.055536\C,0,-0.03196,-2.519625,-2.952734\O,0,0.80713,-3.46032
7,-2.738202\O,0,-1.291052,-2.719693,-3.063911\\Version=ES64L-G16RevA.03\State=1-A
\HF=-3845.3066209\RMSD=3.576e-09\Dipole=-1.5912623,6.9190794,7.9502259\Quadrupole
=28.8091343,-29.9854896,1.1763553,12.0069555,2.8784801,-37.5904481\PG=C01 [X(C61H
52O9P2Pd1)]\

5a

\0,1\C,0,0.111053,3.057626,1.286662\C,0,0.892052,3.343141,0.110832\C,0,1.652877,2.
440381,-0.587658\C,0,-0.487026,1.867037,1.525775\C,0,1.859101,1.042113,-0.287214\
C,0,0.877037,0.310886,0.321328\H,0,0.026117,3.847778,2.02982\H,0,0.988605,4.38867
3,-0.173408\H,0,2.291748,2.842136,-1.370909\H,0,-0.945969,1.636914,2.482652\C,0,3.
182818,0.450124,-0.604044\C,0,3.292426,-0.847444,-1.132466\C,0,4.356433,1.194834,
-0.412129\C,0,4.538335,-1.382856,-1.446285\H,0,2.391364,-1.424843,-1.305321\C,0,5.
603443,0.659684,-0.731953\H,0,4.28611,2.19311,0.008339\C,0,5.699541,-0.632304,-1.
247598\H,0,4.603173,-2.385825,-1.85768\H,0,6.500257,1.250502,-0.570748\H,0,6.6700
92,-1.051653,-1.494723\O,0,1.203941,-0.894409,0.870225\C,0,0.285082,-1.590046,1.6
16436\C,0,-1.572363,-0.275795,0.637064\C,0,-0.525155,0.840114,0.427496\C,0,-1.027
331,-1.304071,1.595029\H,0,-1.720737,-1.851221,2.216699\C,0,-2.893406,0.297675,1.
202081\C,0,-1.781666,-1.051012,-0.671144\C,0,2.200534,-2.922111,2.430811\C,0,0.89
2207,-2.643266,2.41893\H,0,0.193512,-3.217129,3.021508\H,0,2.903151,-2.357295,1.8
28171\H,0,2.592698,-3.726484,3.043164\O,0,-3.241175,0.06811,2.336072\O,0,-3.66872
5,1.094916,0.441692\O,0,-0.997779,-1.069214,-1.596947\O,0,-2.925672,-1.756132,-0.
620519\C,0,-3.401384,1.51557,-0.910983\C,0,-3.217816,-2.601289,-1.763171\H,0,-2.5

80536,0.956581,-1.358279\H,0,-4.308779,1.275501,-1.472319\H,0,-2.287973,-3.065775,
-2.098007\H,0,-3.887321,-3.365633,-1.365102\C,0,-3.871914,-1.80335,-2.877473\C,0,
-3.116944,3.007183,-0.931596\H,0,-2.996261,3.347951,-1.96478\H,0,-3.944742,3.5576
8,-0.477372\H,0,-2.202986,3.237742,-0.379132\H,0,-4.158454,-2.473024,-3.694324\H,
0,-4.77198,-1.299132,-2.514507\H,0,-3.178171,-1.05848,-3.273704\H,0,-0.715257,1.3
58796,-0.516611\\Version=ES64L-G16RevC.01\State=1-A\HF=-1305.7072243\RMSD=4.748e-
09\Dipole=-0.2184192,-0.1133031,-1.4071667\Quadrupole=-1.7488976,10.6540141,-8.90
51165,3.2467482,15.0956769,-1.0595048\PG=C01 [X(C24H24O5)]\

CO_3^{2-}

\-2,1\C,0,0.,0.,0.000026\O,0,0.,0.,1.313191\O,0,0.,-1.137088,-0.656605\O,0,0.,1.1
37088,-0.656605\\Version=ES64L-G16RevC.01\State=1-A1\HF=-264.1058228\RMSD=2.778e-
09\Dipole=0.,0.,-0.0028584\Quadrupole=6.8622895,-3.4336168,-3.4286727,0.,0.,0.\PG
=C02V [C2(C1O1),SGV(O2)]\

3a+CO_3^{2-}

\-2,1\C,0,-5.376087,2.502912,2.430959\C,0,-5.832196,2.82451,1.091992\C,0,-5.54085
8,2.244025,-0.08653\C,0,-4.697671,1.400129,2.819768\C,0,-4.591772,1.129967,-0.450
494\C,0,-4.218702,0.308407,2.002383\C,0,-4.159076,0.193506,0.660096\H,0,-5.643934,
3.222973,3.201917\H,0,-6.530835,3.660369,1.044299\H,0,-6.042464,2.653805,-0.95840
8\H,0,-4.45973,1.310256,3.877673\H,0,-5.120143,0.490498,-1.167172\H,0,-3.766607,-
0.518172,2.542562\N,0,-3.429344,-0.933541,0.145435\S,0,-4.265411,-2.278337,-0.384
75\O,0,-5.666721,-1.963105,-0.730818\O,0,-3.423848,-2.99197,-1.354965\C,0,-3.2324
42,-4.190035,3.036327\C,0,-3.186076,-3.394253,1.897097\C,0,-4.335101,-3.26747,1.1
13148\C,0,-5.514719,-3.919011,1.456466\C,0,-5.543857,-4.714688,2.603534\C,0,-4.40
8479,-4.861337,3.40662\H,0,-2.342473,-4.285146,3.653747\H,0,-2.283403,-2.852694,1.
625757\H,0,-6.393602,-3.78307,0.83608\H,0,-6.464576,-5.222198,2.882336\C,0,-4.442
616,-5.704858,4.658066\H,0,-4.314026,-5.088802,5.556312\H,0,-3.63542,-6.446198,4.
661116\H,0,-5.392485,-6.239528,4.753773\O,0,4.564493,0.154538,-0.661615\C,0,4.756
195,-1.140404,-0.254435\C,0,5.004268,1.232957,0.095655\C,0,3.839221,-2.070748,-0.
792654\C,0,5.773438,-1.528005,0.612824\C,0,5.900399,-2.87459,0.957878\C,0,3.99567
9,-3.414227,-0.42743\C,0,5.018623,-3.818736,0.434219\H,0,3.292693,-4.147461,-0.80
8554\H,0,5.111247,-4.866704,0.708241\H,0,6.677447,-3.1749,1.655525\H,0,6.425265,-
0.775771,1.041036\C,0,4.100911,1.872137,0.952787\C,0,6.299543,1.704268,-0.110695\
C,0,6.73679,2.840329,0.570539\C,0,4.557409,3.023694,1.608138\C,0,5.856963,3.49951
6,1.430829\H,0,3.87224,3.556564,2.258855\H,0,6.178626,4.395177,1.957583\H,0,6.939
605,1.178899,-0.813129\H,0,7.746946,3.212082,0.417681\P,0,2.346794,-1.369343,-1.6
11436\P,0,2.386494,1.182624,1.186712\Pd,0,0.848806,-0.126612,-0.139908\C,0,-3.327
334,1.649672,-1.252436\C,0,-2.388069,0.447678,-1.63214\C,0,-2.187933,-0.50284,-0.
493942\H,0,-2.859061,-0.077149,-2.477561\H,0,-1.423594,0.86111,-1.941074\C,0,-1.0
1864,-0.788809,0.069122\C,0,-0.334331,-1.432237,1.112429\H,0,-0.43143,-1.065706,2.
134165\H,0,-0.063163,-2.485523,1.022113\C,0,-3.691729,2.263861,-2.600193\C,0,-2.5
74152,2.633235,-0.349142\O,0,-2.792655,3.910177,-0.702808\O,0,-4.977025,1.971464,
-2.979227\O,0,-2.93562,2.842458,-3.341915\O,0,-1.949343,2.284171,0.624666\C,0,-2.

025215,4.902894,0.0261\C,0,-2.602183,5.146457,1.413375\H,0,-2.106079,5.79562,-0.6
00634\H,0,-0.979942,4.58024,0.030612\H,0,-3.672483,5.378435,1.357151\H,0,-2.08750
7,5.9937,1.882436\H,0,-2.462582,4.266865,2.042858\C,0,-5.323089,2.372693,-4.30964
1\C,0,-6.743038,1.899907,-4.563076\H,0,-4.611812,1.931378,-5.015574\H,0,-5.22951,
3.460854,-4.397515\H,0,-6.810574,0.813682,-4.453593\H,0,-7.058379,2.169742,-5.577
048\H,0,-7.437555,2.356322,-3.850909\O,0,2.067587,2.708771,-0.116205\C,0,1.016601,
2.779478,-0.938614\O,0,0.575251,3.902191,-1.270693\O,0,0.505673,1.660218,-1.38441
5\C,0,3.151215,-0.071092,2.519531\C,0,4.054074,0.29984,3.531269\C,0,2.841029,-1.4
32146,2.414074\C,0,4.622127,-0.64057,4.391512\H,0,4.325304,1.345997,3.64738\C,0,3.
399553,-2.385123,3.270285\H,0,2.152086,-1.735265,1.631811\C,0,4.296589,-1.994282,
4.263653\H,0,5.321545,-0.319523,5.162659\H,0,3.149974,-3.43609,3.142366\H,0,4.743
747,-2.732908,4.926538\C,0,3.072216,-0.546321,-3.098031\C,0,2.398126,0.586818,-3.
577764\C,0,4.247859,-0.970342,-3.732951\C,0,2.884448,1.257095,-4.703195\H,0,1.536
952,0.970492,-3.027313\C,0,4.729629,-0.295625,-4.853867\H,0,4.799863,-1.815441,-3.
330521\C,0,4.04175,0.816771,-5.347118\H,0,2.362836,2.141645,-5.058686\H,0,5.64475
9,-0.631443,-5.337929\H,0,4.41969,1.347861,-6.218348\C,0,1.502417,2.192418,2.4828
86\C,0,1.189495,3.551669,2.298779\C,0,0.987947,1.553953,3.626017\C,0,0.425563,4.2
44288,3.23894\H,0,1.519016,4.027279,1.382674\C,0,0.199027,2.240624,4.547669\H,0,1.
198543,0.504085,3.790816\C,0,-0.079713,3.596617,4.366504\H,0,0.203089,5.295566,3.
070517\H,0,-0.197662,1.711381,5.411906\H,0,-0.693556,4.135105,5.085646\C,0,1.5825
07,-2.875104,-2.393564\C,0,2.274474,-3.788275,-3.205265\C,0,0.211316,-3.07397,-2.
182502\C,0,1.614389,-4.876755,-3.77452\H,0,3.333617,-3.644194,-3.393915\C,0,-0.45
6037,-4.160482,-2.754554\H,0,-0.339437,-2.358248,-1.580749\C,0,0.246825,-5.066464,
-3.548279\H,0,2.165209,-5.575675,-4.400866\H,0,-1.520401,-4.274811,-2.571661\H,0,
-0.265647,-5.914701,-3.997088\\Version=ES64L-G16RevA.03\State=1-A\HF=-4413.462739
9\RMSD=2.256e-09\Dipole=-2.0740572,-4.5115454,2.6274475\Quadrupole=-12.0130984,-3.
6684361,15.6815346,-14.9269591,-11.3574378,-4.4280605\PG=C01 [X(C62H55N1O10P2Pd1S
1)]\

TS-IV’

\-2,1\C,0,-6.188992,-2.187149,-1.726044\C,0,-6.382092,-2.437039,-0.308806\C,0,-5.
746837,-1.897961,0.748361\C,0,-5.463847,-1.196701,-2.29251\C,0,-4.581507,-0.95367
7,0.843898\C,0,-4.661827,-0.181043,-1.643009\C,0,-4.289027,-0.076956,-0.351067\H,
0,-6.719875,-2.86128,-2.396669\H,0,-7.168629,-3.156463,-0.078951\H,0,-6.097236,-2.
212285,1.729499\H,0,-5.45623,-1.148977,-3.380085\H,0,-4.805708,-0.260485,1.660401
\H,0,-4.233023,0.569038,-2.300587\N,0,-3.345817,0.958027,-0.031211\S,0,-3.955488,
2.41747,0.495066\O,0,-5.259118,2.28515,1.1782\O,0,-2.872555,3.16579,1.146496\C,0,
-3.496094,4.026733,-3.189854\C,0,-3.240686,3.421033,-1.966008\C,0,-4.297061,3.227
329,-1.071182\C,0,-5.590386,3.62122,-1.392516\C,0,-5.830661,4.228346,-2.627769\C,
0,-4.793084,4.439156,-3.539911\H,0,-2.679701,4.173444,-3.893242\H,0,-2.247257,3.0
64933,-1.707989\H,0,-6.386444,3.440549,-0.67906\H,0,-6.841366,4.534438,-2.888614\
C,0,-5.052869,5.076259,-4.883607\H,0,-4.859982,4.371972,-5.702067\H,0,-4.401974,5.
943047,-5.048382\H,0,-6.090352,5.412803,-4.971676\O,0,4.54301,-0.3846,0.625987\C,
0,4.835444,0.874927,0.165517\C,0,4.832247,-1.520295,-0.112458\C,0,4.000714,1.8985

84,0.665793\C,0,5.878994,1.137898,-0.71615\C,0,6.12086,2.454854,-1.113538\C,0,4.2
72757,3.209007,0.251557\C,0,5.324168,3.489599,-0.62516\H,0,3.63781,4.014084,0.606
404\H,0,5.506079,4.514412,-0.939021\H,0,6.919626,2.661611,-1.820355\H,0,6.467836,
0.314744,-1.10487\C,0,3.918196,-1.991583,-1.067098\C,0,5.997664,-2.214451,0.20253
3\C,0,6.288191,-3.412399,-0.450079\C,0,4.227886,-3.202005,-1.699238\C,0,5.396532,
-3.904483,-1.402848\H,0,3.526304,-3.605732,-2.420393\H,0,5.601909,-4.844027,-1.91
0037\H,0,6.648105,-1.808818,0.971051\H,0,7.195575,-3.958703,-0.205374\P,0,2.46982
1,1.344947,1.525218\P,0,2.400037,-0.996659,-1.44276\Pd,0,0.959702,0.162779,0.0583
05\C,0,-3.225,-1.65267,1.322168\C,0,-2.098572,-0.605774,1.558261\C,0,-2.047972,0.
421071,0.488677\H,0,-2.285806,-0.124415,2.530767\H,0,-0.810506,-1.24486,1.521312\
C,0,-0.95582,0.855253,-0.121481\C,0,-0.291852,1.52639,-1.136699\H,0,-0.39398,1.18
8851,-2.167668\H,0,0.030789,2.56053,-1.007934\C,0,-3.419172,-2.332771,2.671531\C,
0,-2.803448,-2.615705,0.211534\O,0,-3.219546,-3.881382,0.418337\O,0,-4.317835,-1.
625095,3.443869\O,0,-2.842717,-3.291743,3.119617\O,0,-2.267915,-2.240941,-0.80906
9\C,0,-2.636383,-4.86038,-0.463247\C,0,-3.333295,-4.895818,-1.817636\H,0,-2.76230
7,-5.807604,0.070934\H,0,-1.569531,-4.63555,-0.534466\H,0,-4.412998,-5.041759,-1.
697281\H,0,-2.937313,-5.721549,-2.423116\H,0,-3.162547,-3.957534,-2.346678\C,0,-4.
420737,-2.0582,4.800169\C,0,-5.37991,-1.110672,5.499002\H,0,-3.427472,-2.046896,5.
261891\H,0,-4.775652,-3.095505,4.831613\H,0,-5.007871,-0.08358,5.445378\H,0,-5.49
2958,-1.388541,6.553171\H,0,-6.365944,-1.137369,5.024342\O,0,1.817509,-2.977323,0.
534539\C,0,0.632265,-2.798351,0.970323\O,0,-0.290568,-3.642767,1.001723\O,0,0.352
948,-1.52677,1.445125\C,0,3.233146,0.159733,-2.664768\C,0,4.173315,-0.27133,-3.61
368\C,0,2.932637,1.524963,-2.598292\C,0,4.795102,0.636371,-4.46965\H,0,4.426468,-
1.325431,-3.672489\C,0,3.552344,2.437228,-3.454488\H,0,2.219917,1.852596,-1.84795
2\C,0,4.485158,1.997082,-4.392684\H,0,5.525019,0.283786,-5.195606\H,0,3.320312,3.
495551,-3.369819\H,0,4.975972,2.707978,-5.053902\C,0,3.167747,0.530238,3.030686\C,
0,2.449059,-0.559202,3.543866\C,0,4.365406,0.919524,3.646432\C,0,2.914442,-1.2201
38,4.683435\H,0,1.564458,-0.912321,3.010991\C,0,4.826919,0.253179,4.7809\H,0,4.95
121,1.729171,3.219619\C,0,4.095218,-0.815096,5.307437\H,0,2.357533,-2.07087,5.067
088\H,0,5.759445,0.561359,5.2499\H,0,4.456408,-1.33952,6.18975\C,0,1.458705,-2.06
7084,-2.620559\C,0,0.945995,-3.306246,-2.192447\C,0,1.088319,-1.584043,-3.886942\
C,0,0.104409,-4.038529,-3.02748\H,0,1.20157,-3.653996,-1.192493\C,0,0.233709,-2.3
19495,-4.709317\H,0,1.46351,-0.627097,-4.232653\C,0,-0.260359,-3.553182,-4.28472\
H,0,-0.290492,-4.987433,-2.675404\H,0,-0.045997,-1.922856,-5.683267\H,0,-0.934061,
-4.123562,-4.920368\C,0,1.814768,2.924081,2.250854\C,0,2.575177,3.836466,3.00009\
C,0,0.44986,3.183218,2.061897\C,0,1.988971,4.986261,3.527625\H,0,3.629396,3.64410
3,3.172761\C,0,-0.142639,4.331516,2.594589\H,0,-0.158436,2.470637,1.51347\C,0,0.6
27876,5.237152,3.323061\H,0,2.591817,5.684899,4.104443\H,0,-1.203571,4.491366,2.4
30901\H,0,0.172393,6.133239,3.739189\\Version=ES64L-G16RevA.03\State=1-A\HF=-4413.
4347443\RMSD=3.176e-09\Dipole=0.8996044,4.8025067,-3.9037465\Quadrupole=-9.585050
1,-14.1055467,23.6905968,8.1238702,10.8738993,-5.5200615\PG=C01 [X(C62H55N1O10P2P
d1S1)]\

int-V’

\-2,1\C,0,-1.6483,4.13498,-3.80063\C,0,-2.863022,3.350655,-3.899131\C,0,-3.358763,
2.418921,-3.063549\C,0,-0.817551,4.229913,-2.735732\C,0,-2.830628,1.849181,-1.776
994\C,0,-0.912782,3.577097,-1.45303\C,0,-1.760034,2.617473,-1.031629\H,0,-1.40192
1,4.726994,-4.680719\H,0,-3.465002,3.557536,-4.784576\H,0,-4.316562,1.981673,-3.3
36032\H,0,0.045764,4.884558,-2.832807\H,0,-3.691248,1.793646,-1.102281\H,0,-0.177
016,3.887764,-0.722042\N,0,-1.611571,2.149195,0.306336\S,0,-2.068811,3.199148,1.5
84368\O,0,-0.940332,3.477607,2.482548\O,0,-2.723098,4.343234,0.928647\C,0,-5.1167
61,0.707072,2.529666\C,0,-4.253985,1.525977,1.81377\C,0,-3.270141,2.249057,2.4917
11\C,0,-3.173861,2.198273,3.880309\C,0,-4.058542,1.386215,4.588621\C,0,-5.01323,0.
60426,3.926218\H,0,-5.832761,0.073515,2.01143\H,0,-4.310689,1.545173,0.733281\H,0,
-2.377833,2.739475,4.377487\H,0,-3.965589,1.313581,5.669897\C,0,-5.86362,-0.40619
3,4.647991\H,0,-5.620105,-1.404071,4.255715\H,0,-6.92894,-0.243611,4.450478\H,0,-
5.69597,-0.374019,5.730322\O,0,3.972066,-1.483905,-0.934425\C,0,4.072923,-2.31018
9,0.166528\C,0,4.845483,-0.421709,-1.067593\C,0,2.890789,-2.974137,0.554963\C,0,5.
276038,-2.489322,0.845552\C,0,5.32697,-3.354274,1.941148\C,0,2.980221,-3.845351,1.
650995\C,0,4.179556,-4.035914,2.341413\H,0,2.082755,-4.357039,1.980415\H,0,4.2092
62,-4.707856,3.194986\H,0,6.262451,-3.481311,2.479221\H,0,6.155953,-1.940822,0.52
989\C,0,4.57799,0.813917,-0.455113\C,0,5.971905,-0.623879,-1.869689\C,0,6.893895,
0.403649,-2.049074\C,0,5.543659,1.819528,-0.633863\C,0,6.682978,1.629184,-1.41366
1\H,0,5.38581,2.781525,-0.159135\H,0,7.402137,2.436576,-1.527085\H,0,6.104222,-1.
596821,-2.332408\H,0,7.771374,0.245377,-2.67051\P,0,1.263524,-2.383388,-0.087482\
P,0,2.982307,1.077272,0.530316\C,0,1.220153,-2.893797,-1.864729\C,0,0.098301,-3.6
89867,0.537564\C,0,3.810378,1.102578,2.193265\C,0,2.685394,2.870643,0.205483\C,0,
4.523744,2.19293,2.70878\C,0,3.737891,-0.075936,2.951073\C,0,4.387938,-0.170774,4.
180855\C,0,5.154909,2.106616,3.950594\C,0,5.096752,0.921172,4.686754\H,0,4.569367,
3.119372,2.144871\H,0,3.149781,-0.905157,2.569881\H,0,4.328553,-1.097014,4.746038
\H,0,5.694102,2.966034,4.343128\H,0,5.591849,0.853351,5.652686\C,0,2.312545,-3.35
0778,-2.612914\C,0,-0.025443,-2.774951,-2.505862\C,0,-0.176099,-3.095606,-3.85068
6\C,0,0.924179,-3.536473,-4.593179\C,0,2.164885,-3.663764,-3.967029\H,0,-0.896387,
-2.436592,-1.957372\H,0,-1.156258,-2.982627,-4.303654\H,0,0.814425,-3.780335,-5.6
47365\H,0,3.026101,-4.016026,-4.531373\H,0,3.280823,-3.465607,-2.141104\C,0,0.184
682,-5.037972,0.152887\C,0,-0.973407,-3.280259,1.339876\C,0,-1.961665,-4.188615,1.
740757\C,0,-0.783533,-5.9512,0.566169\C,0,-1.861739,-5.524762,1.350311\H,0,1.0015
28,-5.361016,-0.487157\H,0,-1.042665,-2.239157,1.638875\H,0,-2.818072,-3.834335,2.
316179\H,0,-0.710926,-6.99248,0.258695\H,0,-2.63187,-6.233141,1.646039\C,0,2.9772
84,3.509736,-1.009836\C,0,1.925608,3.571303,1.160056\C,0,1.538405,4.890052,0.9373
98\C,0,2.583063,4.830549,-1.23559\C,0,1.878104,5.533345,-0.257676\H,0,3.509708,2.
973977,-1.788155\H,0,1.587805,3.061981,2.055851\H,0,0.923728,5.388185,1.679565\H,
0,2.821931,5.305483,-2.184569\H,0,1.559687,6.556558,-0.439011\Pd,0,1.130765,-0.18
9294,0.61882\C,0,-2.358518,0.351401,-1.866343\C,0,-1.912915,-0.094108,-0.496496\C,
0,-1.493083,0.728469,0.5044\H,0,-1.72522,-1.151556,-0.378375\H,0,-3.970314,-2.977
29,0.597997\C,0,-0.710001,0.235021,1.636673\C,0,-1.228744,-0.18236,2.801305\H,0,-
0.584058,-0.600955,3.57361\H,0,-2.293381,-0.178522,3.030236\C,0,-3.455982,-0.6391
55,-2.260849\C,0,-1.193605,0.30574,-2.872738\O,0,-1.621314,0.082816,-4.140827\O,0,

-4.692626,-0.17708,-2.021082\O,0,-3.21917,-1.759959,-2.670369\O,0,-0.036306,0.518
706,-2.592745\C,0,-0.591904,0.057533,-5.147543\C,0,-0.250086,1.46029,-5.625057\H,
0,-1.011064,-0.551543,-5.953826\H,0,0.286168,-0.447603,-4.74057\H,0,-1.138337,1.9
65212,-6.015683\H,0,0.506605,1.411838,-6.417481\H,0,0.14233,2.054294,-4.797957\C,
0,-5.773899,-1.133861,-2.189301\C,0,-6.983242,-0.600615,-1.448064\H,0,-5.453182,-
2.086633,-1.769516\H,0,-5.947608,-1.24153,-3.26778\H,0,-6.848488,-0.733587,-0.369
031\H,0,-7.866739,-1.180974,-1.740773\H,0,-7.168055,0.450908,-1.6968\O,0,-4.83563
9,-2.789663,2.645197\C,0,-5.476411,-2.404217,1.636263\O,0,-6.593993,-1.84842,1.55
3501\O,0,-4.842015,-2.61371,0.388467\\Version=ES64L-G16RevA.03\State=1-A\HF=-4413.
4897843\RMSD=2.216e-09\Dipole=8.6637229,1.2407251,-4.3968301\Quadrupole=-46.27838
21,3.9857409,42.2926412,-21.7315398,26.2190634,-3.3148394\PG=C01 [X(C62H55N1O10P2
Pd1S1)]\

TS-V'

\-2,1\C,0,-0.990507,-2.441777,2.821485\C,0,-1.541185,-1.920872,4.05232\C,0,-2.140
833,-0.694408,4.181267\C,0,-1.491463,-2.16383,1.59691\C,0,-2.338214,0.286625,3.14
6758\C,0,-2.57934,-0.013402,1.842276\H,0,-0.091163,-3.050739,2.892192\H,0,-1.3290
54,-2.473119,4.967173\H,0,-2.355988,-0.358725,5.195968\H,0,-0.960936,-2.44094,0.6
92526\C,0,-2.232391,0.4693,-0.575509\C,0,-3.448312,-1.618681,0.083291\C,0,-2.8205
23,-1.460043,1.483644\C,0,-2.600795,-0.804176,-0.874855\H,0,-2.179536,-1.271265,-
1.770565\C,0,-3.479029,-3.106061,-0.350147\C,0,-4.878468,-1.090028,0.052545\C,0,-
1.23368,2.504087,-1.72475\C,0,-1.360909,1.189923,-1.478018\H,0,-0.465639,0.400689,
-2.305709\H,0,-1.79248,3.318433,-1.270625\H,0,-0.467471,2.81862,-2.433643\O,0,-2.
804069,-3.548365,-1.23961\O,0,-4.351538,-3.976634,0.25521\O,0,-5.457315,-0.518639,
0.958533\O,0,-5.446332,-1.353786,-1.145574\C,0,-5.031142,-3.782341,1.503524\C,0,-
6.822193,-0.972126,-1.302782\H,0,-5.189293,-2.72906,1.729781\H,0,-6.017944,-4.237
526,1.359987\H,0,-6.992745,-0.022144,-0.797144\H,0,-6.950313,-0.835247,-2.379155\
C,0,-7.739173,-2.059922,-0.761516\C,0,-4.274937,-4.483178,2.623882\H,0,-4.828653,
-4.396092,3.565827\H,0,-4.146403,-5.543632,2.387145\H,0,-3.285772,-4.040075,2.762
066\H,0,-8.786502,-1.817834,-0.976363\H,0,-7.50085,-3.02567,-1.21693\H,0,-7.62058
7,-2.144503,0.321779\H,0,-3.479283,-1.849236,2.265825\O,0,4.922156,0.183933,0.279
87\C,0,5.273548,1.51747,0.274031\C,0,4.380013,-0.406736,1.402409\C,0,4.311414,2.5
28368,0.066318\C,0,6.633627,1.80504,0.390759\C,0,7.081477,3.123399,0.326231\C,0,4.
79575,3.846685,0.017051\C,0,6.152296,4.147579,0.144016\H,0,4.087658,4.653399,-0.1
33223\H,0,6.479234,5.183637,0.099057\H,0,8.14186,3.343944,0.417788\H,0,7.319102,0.
974053,0.523772\C,0,3.507762,-1.488083,1.16447\C,0,4.693203,0.027464,2.686628\C,0,
4.1367,-0.628132,3.786272\C,0,2.975469,-2.129728,2.292165\C,0,3.283235,-1.711472,
3.587731\H,0,2.287041,-2.953853,2.145415\H,0,2.832186,-2.216366,4.437866\H,0,5.34
2794,0.886084,2.815763\H,0,4.356991,-0.274135,4.78928\P,0,2.508242,2.073509,-0.15
4552\P,0,2.864435,-1.727049,-0.552831\C,0,2.070773,1.965854,1.647318\C,0,1.790212,
3.731025,-0.56304\C,0,4.360814,-2.241309,-1.504798\C,0,2.039869,-3.381979,-0.4049
3\C,0,5.586345,-2.586665,-0.922314\C,0,4.219543,-2.277207,-2.902983\C,0,5.303513,
-2.667032,-3.691499\C,0,6.667288,-2.965195,-1.720542\C,0,6.527741,-3.007491,-3.10
9177\H,0,5.697145,-2.55316,0.1577\H,0,3.256559,-2.005656,-3.350928\H,0,5.18847,-2.

697146,-4.772456\H,0,7.61721,-3.226365,-1.257604\H,0,7.369696,-3.301444,-3.733156
\C,0,2.561874,2.873184,2.598085\C,0,1.246417,0.919311,2.070498\C,0,0.940456,0.763
91,3.422985\C,0,1.437407,1.666364,4.361713\C,0,2.245464,2.728272,3.946865\H,0,0.8
69611,0.220135,1.329926\H,0,0.324318,-0.067986,3.736365\H,0,1.199334,1.540242,5.4
15605\H,0,2.634503,3.437514,4.674638\H,0,3.201762,3.68998,2.278332\C,0,0.698227,4.
25639,0.143244\C,0,2.179315,4.371754,-1.754262\C,0,1.538965,5.528214,-2.188896\C,
0,0.039638,5.402061,-0.305061\C,0,0.463168,6.052412,-1.462964\H,0,0.329828,3.7498
06,1.026963\H,0,2.982038,3.943706,-2.349431\H,0,1.864059,6.010463,-3.108219\H,0,-
0.827703,5.757953,0.243128\H,0,-0.054111,6.941585,-1.815355\C,0,0.74225,-3.529552,
-0.910856\C,0,2.694223,-4.499554,0.140215\C,0,2.049872,-5.731798,0.21979\C,0,0.09
9369,-4.771408,-0.838484\C,0,0.744359,-5.865814,-0.265685\H,0,0.225153,-2.705891,
-1.398952\H,0,3.713637,-4.398141,0.502363\H,0,2.565192,-6.587488,0.652277\H,0,-0.
906475,-4.855412,-1.2338\H,0,0.238788,-6.827598,-0.206108\Pd,0,1.957542,0.238511,
-1.291752\O,0,0.449413,-0.005613,-2.9167\C,0,0.310051,-1.32184,-3.349676\O,0,1.33
1803,-1.839143,-3.8727\O,0,-0.812923,-1.864369,-3.154355\S,0,-3.488697,2.383242,1.
023515\N,0,-2.621516,0.935757,0.792486\O,0,-4.139223,2.327875,2.339771\O,0,-2.641
104,3.543288,0.735377\C,0,-4.811992,2.350686,-0.199576\C,0,-6.110215,2.506501,0.2
88268\C,0,-4.568246,2.256464,-1.571176\C,0,-7.173378,2.585224,-0.607446\H,0,-6.26
6266,2.555032,1.358625\C,0,-5.650719,2.309891,-2.44888\H,0,-3.55827,2.153879,-1.9
53284\H,0,-8.184784,2.714645,-0.228458\H,0,-5.462948,2.220735,-3.515622\C,0,-8.12
2833,2.49105,-2.950632\H,0,-8.497871,1.474182,-3.127014\H,0,-7.832664,2.900934,-3.
922903\H,0,-8.958727,3.083439,-2.564163\C,0,-6.960955,2.478792,-1.988034\H,0,-2.2
89271,1.325627,3.44591\\Version=ES64L-G16RevA.03\State=1-A\HF=-4413.4540607\RMSD=
6.031e-09\Dipole=-2.5043837,1.5066008,7.1408665\Quadrupole=32.2781015,-14.4623885,
-17.815713,14.4033622,24.6271999,-38.6330516\PG=C01 [X(C62H55N1O10P2Pd1S1)]\

3a’+PdL$_n$+CO$_3^{2-}$

\-2,1\C,0,-1.242694,-2.141828,3.026297\C,0,-1.838501,-1.639305,4.244015\C,0,-2.48
9081,-0.439652,4.372915\C,0,-1.712717,-1.889392,1.783688\C,0,-2.689316,0.540816,3.
337478\C,0,-2.84886,0.236611,2.024624\H,0,-0.33176,-2.726549,3.128546\H,0,-1.6250
31,-2.189707,5.159304\H,0,-2.741267,-0.122906,5.384575\H,0,-1.147907,-2.170115,0.
898892\C,0,-2.334079,0.713327,-0.307414\C,0,-3.627634,-1.329809,0.198811\C,0,-3.0
51434,-1.206947,1.627919\C,0,-2.724658,-0.513866,-0.707852\H,0,-2.24856,-0.943042,
-1.608814\C,0,-3.710808,-2.796167,-0.314517\C,0,-5.069068,-0.813516,0.137413\C,0,
-0.700795,2.575785,-0.680682\C,0,-1.314046,1.44205,-1.051393\H,0,-0.963292,0.9272
29,-1.955909\H,0,-0.918315,3.107497,0.236625\H,0,0.085458,2.966596,-1.31444\O,0,-
3.123926,-3.177673,-1.288406\O,0,-4.583249,-3.679968,0.270369\O,0,-5.739528,-0.43
3611,1.077992\O,0,-5.530489,-0.906655,-1.127067\C,0,-5.165897,-3.597915,1.581376\
C,0,-6.950693,-0.794902,-1.316676\H,0,-5.354673,-2.568272,1.881377\H,0,-6.139628,
-4.089301,1.473152\H,0,-7.353823,-0.071376,-0.608792\H,0,-7.068231,-0.406321,-2.3
30187\C,0,-7.599491,-2.161602,-1.153126\C,0,-4.30652,-4.34029,2.594291\H,0,-4.794
781,-4.336985,3.575582\H,0,-4.159687,-5.376734,2.276715\H,0,-3.325475,-3.871236,2.
696036\H,0,-8.664833,-2.108633,-1.405279\H,0,-7.116879,-2.89653,-1.802595\H,0,-7.
507833,-2.504109,-0.118853\H,0,-3.733805,-1.621776,2.373633\O,0,4.962297,-0.37805

8,0.310752\C,0,5.530411,0.875786,0.325188\C,0,4.249263,-0.848071,1.395551\C,0,4.7
50188,2.035784,0.136673\C,0,6.919839,0.930495,0.433261\C,0,7.581305,2.156652,0.37
4163\C,0,5.44719,3.255458,0.08836\C,0,6.836366,3.323803,0.205383\H,0,4.882399,4.1
70046,-0.052939\H,0,7.333032,4.290206,0.161225\H,0,8.664414,2.195723,0.457982\H,0,
7.457578,-0.004933,0.551139\C,0,3.189855,-1.729786,1.105564\C,0,4.584385,-0.48636
3,2.69654\C,0,3.847833,-0.999824,3.765578\C,0,2.483865,-2.241256,2.205972\C,0,2.7
98082,-1.881799,3.516651\H,0,1.657864,-2.917392,2.0197\H,0,2.206714,-2.275264,4.3
3952\H,0,5.395479,0.214943,2.860365\H,0,4.085743,-0.692675,4.779888\P,0,2.902473,
1.895191,-0.124789\P,0,2.558773,-1.858566,-0.629923\C,0,2.367964,1.862834,1.65562
7\C,0,2.56243,3.681257,-0.545754\C,0,3.998019,-2.529625,-1.57718\C,0,1.575698,-3.
433265,-0.493665\C,0,5.15756,-3.051487,-0.990544\C,0,3.877283,-2.503251,-2.977452
\C,0,4.91927,-2.99995,-3.76313\C,0,6.196155,-3.538653,-1.785785\C,0,6.079773,-3.5
13059,-3.177126\H,0,5.254087,-3.069577,0.091006\H,0,2.947374,-2.114015,-3.416908\
H,0,4.821418,-2.981215,-4.846449\H,0,7.095328,-3.936532,-1.318384\H,0,6.889287,-3.
89118,-3.799264\C,0,3.040561,2.538121,2.68468\C,0,1.236562,1.101165,1.973566\C,0,
0.786293,1.014909,3.290825\C,0,1.460293,1.693683,4.306262\C,0,2.589418,2.457542,4.
00183\H,0,0.731998,0.567064,1.17359\H,0,-0.081696,0.409514,3.519265\H,0,1.111116,
1.619629,5.33382\H,0,3.123662,2.983027,4.790992\H,0,3.927437,3.120399,2.4518\C,0,
2.005689,4.633189,0.320582\C,0,2.751879,4.039899,-1.892785\C,0,2.434032,5.315521,
-2.34837\C,0,1.655086,5.90426,-0.143438\C,0,1.875968,6.256216,-1.474146\H,0,1.828
315,4.375897,1.358742\H,0,3.114779,3.282411,-2.583723\H,0,2.592728,5.569483,-3.39
3797\H,0,1.203145,6.618773,0.541075\H,0,1.597532,7.243564,-1.834825\C,0,0.28045,-
3.487771,-1.026882\C,0,2.13026,-4.586858,0.086784\C,0,1.397085,-5.767695,0.174426
\C,0,-0.449244,-4.681893,-0.942961\C,0,0.097383,-5.812855,-0.339884\H,0,-0.16556,
-2.634271,-1.540756\H,0,3.14452,-4.554877,0.474463\H,0,1.838956,-6.649798,0.63471
\H,0,-1.449834,-4.705214,-1.35817\H,0,-0.481435,-6.732783,-0.277761\Pd,0,2.020707,
0.290113,-1.350494\O,0,0.625297,0.214544,-3.00561\C,0,0.206521,-1.040552,-3.21628
6\O,0,1.025714,-1.917283,-3.650076\O,0,-1.02086,-1.317787,-2.945555\S,0,-3.77312,
2.619182,1.107097\N,0,-2.817615,1.198284,0.982114\O,0,-4.624891,2.475803,2.293656
\O,0,-2.924208,3.807439,0.992948\C,0,-4.825446,2.589998,-0.340503\C,0,-6.199533,2.
476044,-0.142573\C,0,-4.286811,2.76535,-1.617901\C,0,-7.047211,2.537281,-1.247368
\H,0,-6.579073,2.334897,0.861765\C,0,-5.14868,2.785121,-2.709324\H,0,-3.215811,2.
871362,-1.751767\H,0,-8.122245,2.460122,-1.102122\H,0,-4.732522,2.889868,-3.70740
6\C,0,-7.452214,2.669026,-3.741989\H,0,-7.414228,1.703006,-4.260906\H,0,-7.16254,
3.433742,-4.470295\H,0,-8.492169,2.849122,-3.452448\C,0,-6.536163,2.673313,-2.542
995\H,0,-2.67643,1.583109,3.634782\\Version=ES64L-G16RevA.03\State=1-A\HF=-4413.4
841341\RMSD=3.531e-09\Dipole=-3.6372819,1.8664814,7.2259924\Quadrupole=28.2214274,
-12.9556428,-15.2657846,16.3007384,30.2272119,-40.6582494\PG=C01 [X(C62H55N1O10P2
Pd1S1)]\

int-IV-inner

\-2,1\C,0,-5.897782,-2.865324,-0.842626\C,0,-4.796857,-3.163399,-1.717355\C,0,-3.
495888,-3.170317,-1.36366\C,0,-5.820344,-2.062104,0.260059\C,0,-3.004652,-3.14564
3,0.066709\C,0,-4.683221,-1.401087,0.847216\C,0,-3.372889,-1.822673,0.743027\H,0,

-6.887975,-3.20047,-1.148195\H,0,-5.037575,-3.353759,-2.764078\H,0,-2.74717,-3.26
3985,-2.139836\H,0,-6.765392,-1.817739,0.745078\H,0,-3.558767,-3.92475,0.609642\O,
0,1.235962,2.632856,-0.265638\C,0,2.306043,3.149392,0.455283\C,0,0.871404,3.13313
2,-1.482757\C,0,3.36504,2.299809,0.824797\C,0,2.208063,4.474661,0.882658\C,0,3.18
1185,5.007073,1.723685\C,0,4.304559,2.865992,1.709622\C,0,4.229296,4.187079,2.146
672\H,0,5.121537,2.246219,2.061041\H,0,4.989338,4.573585,2.821959\H,0,3.10505,6.0
36976,2.062781\H,0,1.347625,5.058222,0.57138\C,0,-0.45734,2.858905,-1.872812\C,0,
1.746465,3.824534,-2.316968\C,0,1.304961,4.234195,-3.575295\C,0,-0.870171,3.28049
4,-3.138441\C,0,0.002473,3.962433,-3.991741\H,0,-1.886457,3.069998,-3.455966\H,0,
-0.336085,4.277502,-4.975248\H,0,2.764957,3.996647,-1.9914\H,0,1.994277,4.752384,
-4.236941\P,0,3.383774,0.436405,0.423824\P,0,-1.463825,1.876447,-0.67715\Pd,0,1.7
60049,-0.628225,1.714549\C,0,-1.502831,-3.506026,0.180601\C,0,-0.938057,-2.963177,
1.512646\C,0,-1.143213,-1.492591,1.641171\H,0,-1.45222,-3.504303,2.323156\H,0,0.1
33347,-3.166197,1.589436\C,0,-0.222227,-0.636666,2.0592\C,0,0.19582,0.704134,2.21
243\H,0,0.029682,1.436643,1.431926\H,0,0.232763,1.12769,3.218337\C,0,-1.313417,-5.
022081,0.067556\C,0,-0.663742,-2.875636,-0.949781\O,0,0.316554,-3.694238,-1.30691
2\O,0,-0.285379,-5.430475,0.814671\O,0,-1.981677,-5.749677,-0.6443\O,0,-0.884349,
-1.774367,-1.421036\C,0,1.356952,-3.229473,-2.219901\C,0,0.874522,-3.292657,-3.65
6484\H,0,2.197418,-3.883644,-1.98493\H,0,1.65346,-2.228037,-1.922489\H,0,0.568783,
-4.309038,-3.931378\H,0,1.688149,-2.981525,-4.320485\H,0,0.030228,-2.616135,-3.81
622\C,0,0.376569,-6.656938,0.447609\C,0,1.764327,-6.599227,1.056971\H,0,0.411484,
-6.704617,-0.645318\H,0,-0.224362,-7.505151,0.802718\H,0,2.300102,-5.695876,0.714
908\H,0,2.328072,-7.494519,0.763848\H,0,1.700438,-6.578055,2.150583\O,0,3.563755,
-1.886691,-0.449277\C,0,3.144136,-2.88553,0.27219\O,0,3.324925,-4.088355,-0.09495
2\O,0,2.511783,-2.637929,1.412718\C,0,-1.865374,3.206286,0.549297\C,0,-1.597952,4.
568476,0.353712\C,0,-2.440947,2.797599,1.762337\C,0,-1.892324,5.503155,1.348375\H,
0,-1.151475,4.896041,-0.579698\C,0,-2.740893,3.733071,2.751346\H,0,-2.638064,1.74
4544,1.933566\C,0,-2.463968,5.086948,2.551279\H,0,-1.671909,6.555388,1.182567\H,0,
-3.185441,3.395708,3.681635\H,0,-2.68732,5.811962,3.330437\C,0,2.964222,0.546481,
-1.372682\C,0,1.669251,0.163523,-1.743635\C,0,3.836888,1.00041,-2.365147\C,0,1.24
9384,0.251335,-3.067011\H,0,0.98627,-0.198192,-0.983911\C,0,3.422059,1.075046,-3.
698948\H,0,4.85503,1.272218,-2.113919\C,0,2.125471,0.708802,-4.054778\H,0,0.23649
6,-0.049776,-3.313547\H,0,4.118201,1.42722,-4.457793\H,0,1.796992,0.783101,-5.088
57\C,0,-3.095088,1.740925,-1.537509\C,0,-3.45944,0.461347,-1.982843\C,0,-4.00509,
2.799004,-1.687458\C,0,-4.704358,0.248847,-2.578686\H,0,-2.774463,-0.368564,-1.83
5535\C,0,-5.246746,2.586729,-2.285012\H,0,-3.741511,3.785117,-1.317391\C,0,-5.597
814,1.309267,-2.732664\H,0,-4.979027,-0.754235,-2.885546\H,0,-5.945677,3.413471,-
2.39138\H,0,-6.572182,1.139585,-3.18497\C,0,5.211,0.162694,0.553226\C,0,6.193022,
1.031196,0.059326\C,0,5.619515,-0.982731,1.255647\C,0,7.552808,0.757,0.236933\H,0,
5.899974,1.949542,-0.438695\C,0,6.973963,-1.2482,1.446554\H,0,4.858162,-1.654419,
1.637417\C,0,7.946908,-0.385889,0.930463\H,0,8.298453,1.441664,-0.162309\H,0,7.27
088,-2.141275,1.990528\H,0,9.003673,-0.602702,1.073014\O,0,-2.450144,-1.029414,1.
297528\C,0,-4.996604,-0.183004,1.645334\C,0,-4.600622,-0.042897,2.986325\C,0,-5.7
5293,0.848191,1.06714\C,0,-4.974773,1.074578,3.728515\H,0,-3.992429,-0.818354,3.4

3913\C,0,-6.117385,1.974055,1.806051\H,0,-6.025851,0.77048,0.020151\C,0,-5.74001,
2.087961,3.143206\H,0,-4.661445,1.159153,4.766151\H,0,-6.682373,2.770011,1.327697
\H,0,-6.016728,2.967208,3.719028\\Version=ES64L-G16RevC.01\State=1-A\HF=-3845.294
1664\RMSD=5.304e-09\Dipole=-4.7820625,7.1211561,-1.634828\Quadrupole=-23.4981869,
-10.8729446,34.3711315,22.3794157,-1.9492956,-0.2283573\PG=C01 [X(C61H52O9P2Pd1)]
\

int-V-inner

\-2,1\C,0,-1.946419,-2.55581,2.137673\C,0,-2.355916,-1.822852,3.306159\C,0,-2.664
139,-0.483632,3.330054\C,0,-2.361064,-2.267996,0.878925\C,0,-2.664933,0.45394,2.2
36096\C,0,-2.937396,0.052475,0.947091\H,0,-1.202177,-3.338259,2.274521\H,0,-2.262
174,-2.322145,4.270418\H,0,-2.790025,-0.032331,4.313795\H,0,-1.878517,-2.679516,-
0.004897\C,0,-2.33059,1.861994,2.556611\C,0,-3.048793,2.931055,1.99679\C,0,-1.309
392,2.153529,3.475331\C,0,-2.763394,4.24422,2.35744\H,0,-3.826868,2.71764,1.27247
\C,0,-1.023287,3.469371,3.832854\H,0,-0.709065,1.342242,3.870227\C,0,-1.753689,4.
520883,3.281427\H,0,-3.316248,5.05931,1.900265\H,0,-0.205597,3.670081,4.520409\H,
0,-1.515641,5.549109,3.535903\O,0,-2.673624,0.884273,-0.0732\C,0,-2.808623,0.4737
54,-1.407396\C,0,-4.244816,-1.425792,-0.645333\C,0,-3.511607,-1.315763,0.708447\C,
0,-3.511826,-0.644762,-1.706882\H,0,-3.562021,-0.979556,-2.730976\C,0,-4.446877,-
2.895324,-1.076087\C,0,-5.579433,-0.692089,-0.57861\C,0,-2.784028,1.918848,-3.309
614\C,0,-2.076519,1.319867,-2.329505\H,0,-3.876281,1.822498,-3.391517\H,0,-2.3035
68,2.511812,-4.086352\O,0,-3.9404,-3.378774,-2.057922\O,0,-5.254276,-3.715698,-0.
337643\O,0,-5.887852,0.174074,0.220249\O,0,-6.406151,-1.09533,-1.586061\C,0,-5.97
7141,-3.346662,0.842946\C,0,-7.620689,-0.347659,-1.736678\H,0,-5.986788,-2.269699,
1.003264\H,0,-7.013452,-3.659607,0.665283\H,0,-7.412702,0.710587,-1.558717\H,0,-7.
908737,-0.489271,-2.781951\C,0,-8.700458,-0.853182,-0.789143\C,0,-5.389719,-4.063
085,2.049539\H,0,-5.983576,-3.843816,2.944292\H,0,-5.390172,-5.145104,1.884763\H,
0,-4.361035,-3.738763,2.22329\H,0,-9.646776,-0.331349,-0.973735\H,0,-8.862543,-1.
926586,-0.93031\H,0,-8.399442,-0.673386,0.245165\H,0,-4.212288,-1.477834,1.533466
\O,0,3.870327,0.88613,-0.468009\C,0,4.023396,2.217958,-0.81731\C,0,3.405236,-0.00
868,-1.412264\C,0,2.93942,3.114409,-0.717584\C,0,5.294514,2.621443,-1.217498\C,0,
5.528081,3.949913,-1.576174\C,0,3.202542,4.442016,-1.114226\C,0,4.465746,4.856005,
-1.533521\H,0,2.394071,5.163433,-1.082981\H,0,4.621518,5.894212,-1.819039\H,0,6.5
18524,4.26549,-1.893891\H,0,6.081934,1.87415,-1.247699\C,0,3.376446,-1.354999,-1.
02476\C,0,2.974863,0.394126,-2.680256\C,0,2.534854,-0.566029,-3.586391\C,0,2.9623
49,-2.301936,-1.977835\C,0,2.549478,-1.91916,-3.247549\H,0,2.91377,-3.346543,-1.6
9154\H,0,2.160485,-2.656963,-3.940095\H,0,2.957953,1.445413,-2.935943\H,0,2.13354
9,-0.247465,-4.542393\P,0,1.218058,2.461937,-0.420348\P,0,3.780693,-1.76043,0.722
163\C,0,1.466654,1.571795,1.191956\C,0,0.412757,4.074452,0.101559\C,0,2.822777,-3.
330381,0.953683\C,0,5.504331,-2.443539,0.559436\C,0,1.483626,-3.381298,0.524864\C,
0,3.358286,-4.434605,1.634344\C,0,2.581591,-5.569478,1.874325\C,0,0.724143,-4.533
411,0.737217\C,0,1.2629,-5.626633,1.419281\H,0,1.026368,-2.552463,-0.018913\H,0,4.
39233,-4.419028,1.965912\H,0,3.017043,-6.416706,2.400907\H,0,-0.279637,-4.562677,
0.32602\H,0,0.664813,-6.520115,1.584322\C,0,2.301473,2.014125,2.227228\C,0,0.7970

23,0.347201,1.340147\C,0,0.944425,-0.401907,2.508628\C,0,1.759818,0.058852,3.5428
47\C,0,2.443825,1.26802,3.397316\H,0,0.192765,-0.03368,0.521608\H,0,0.423499,-1.3
48011,2.596989\H,0,1.878144,-0.530427,4.449479\H,0,3.100619,1.623662,4.188982\H,0,
2.872962,2.929337,2.106447\C,0,0.935963,4.963264,1.051741\C,0,-0.751739,4.441161,
-0.585054\C,0,-1.351451,5.682213,-0.358592\C,0,0.343594,6.206489,1.274015\C,0,-0.
799043,6.57494,0.560009\H,0,1.830206,4.697404,1.604415\H,0,-1.158759,3.728425,-1.
302285\H,0,-2.253818,5.94818,-0.904481\H,0,0.773479,6.887469,2.006047\H,0,-1.2606
39,7.545617,0.730823\C,0,6.275659,-2.55132,1.727822\C,0,6.091791,-2.818561,-0.657
577\C,0,7.400346,-3.300798,-0.701127\C,0,7.579603,-3.047678,1.690499\C,0,8.148022,
-3.423675,0.472277\H,0,5.844041,-2.233409,2.673865\H,0,5.513078,-2.725842,-1.5708
89\H,0,7.837386,-3.585593,-1.655514\H,0,8.155732,-3.128057,2.609489\H,0,9.167381,
-3.800615,0.436166\Pd,0,-0.016719,1.30868,-1.821652\O,0,0.023465,-1.302488,-1.304
979\C,0,-0.508846,-1.980469,-2.217263\O,0,-0.665899,-1.773124,-3.428794\O,0,-1.00
6618,-3.241943,-1.741748\H,0,-1.619297,-3.525031,-2.435379\\Version=ES64L-G16RevC.
01\State=1-A\HF=-3845.2783582\RMSD=4.892e-09\Dipole=1.0848637,0.3196604,5.1966963
\Quadrupole=14.3547548,-3.4765371,-10.8782177,3.0058173,-5.3398266,-11.5406203\PG
=C01 [X(C61H52O9P2Pd1)]\

(I) References

1 R. Manzano, A. Romaniega, L. Prieto, E. Díaz, E. Reyes, U. Uria, L. Carrillo and J. L. Vicario, *Org. Lett.*, 2020, **22**, 4721.

2 J. Chen, S. Gao, and M. Chen, *Org. Lett.*, 2019, **21**, 8800.

3 S. N. Ononye, M. D. E. Z. VanHeyst, Oblak, W.-D. Zhou, M. Ammar, A. C. Anderson and. D. L. Wright, *ACS Med. Chem. Lett.*, 2013, **4**, 757.

4 Long, Y.-J.; Shen, J.-H.; Wei, Y.; Shi, M. *J. Org. Chem.* **2024**, *89*, 14831.

5 Gaussian 16, Revision A.03, Frisch, M. J.; Trucks, G. W.; Schlegel, H. B.; Scuseria, G. E.; Robb, M. A.; Cheeseman, J. R.; Scalmani, G.; Barone, V.; Petersson, G. A.; Nakatsuji, H.; Li, X.; Caricato, M.; Marenich, A. V.; Bloino, J.; Janesko, B. G.; Gomperts, R.; Mennucci, B.; Hratchian, H. P.; Ortiz, J. V.; Izmaylov, A. F.; Sonnenberg, J. L.; Williams-Young, D.; Ding, F.; Lipparini, F.; Egidi, F.; Goings, J.; Peng, B.; Petrone, A.; Henderson, T.; Ranasinghe, D.; Zakrzewski, V. G.; Gao, J.; Rega, N.; Zheng, G.; Liang, W.; Hada, M.; Ehara, M.; Toyota, K.; Fukuda, R.; Hasegawa, J.; Ishida, M.; Nakajima, T.; Honda, Y.; Kitao, O.; Nakai, H.; Vreven, T.; Throssell, K.; Montgomery, J. A., Jr.; Peralta, J. E.; Ogliaro, F.; Bearpark, M. J.; Heyd, J. J.; Brothers, E. N.; Kudin, K. N.; Staroverov, V. N.; Keith, T. A.; Kobayashi, R.; Normand, J.; Raghavachari, K.; Rendell, A. P.; Burant, J. C.; Iyengar, S. S.; Tomasi, J.; Cossi, M.; Millam, J. M.; Klene, M.; Adamo, C.; Cammi, R.; Ochterski, J. W.; Martin, R. L.; Morokuma, K.; Farkas, O.; Foresman, J. B.; Fox, D. J. Gaussian, Inc., Wallingford CT, 2016.
